# Lysophosphatidic acid–mediated NF-**κ**B activation promotes FOXC2 expression essential for lymphatic valve development

**DOI:** 10.1172/JCI193364

**Published:** 2026-01-13

**Authors:** Daisuke Yasuda, Nana Sato, Keisuke Yanagida, Tomomi Hashidate-Yoshida, Tomohiro Shiiya, Hideo Shindou, Atsuki Taira, Takashi Ebihara, Takao Shimizu, Masanori Hirashima, Seiya Mizuno, Satoru Takahashi, Satoshi Ishii

**Affiliations:** 1 Department of Immunology, Akita University Graduate School of Medicine, Akita, Japan.; 2 Department of Lipid Life Science, National Institute of Global Health and Medicine, Japan Institute for Health Security, Tokyo, Japan.; 3 Department of Molecular Biology, The Jikei University School of Medicine, Tokyo, Japan.; 4 Division of Pharmacology, Graduate School of Medical and Dental Sciences, Niigata University, Niigata, Japan.; 5 Department of Medical Lipid Science, Graduate School of Medicine, The University of Tokyo, Tokyo, Japan.; 6 Department of Medical Biology, Akita University Graduate School of Medicine, Akita, Japan.; 7 Institute of Microbial Chemistry, Tokyo, Japan.; 8 Laboratory Animal Resource Center, Transborder Medical Research Center, Institute of Medicine, University of Tsukuba, Ibaraki, Japan.

**Keywords:** Cell biology, Development, Vascular biology, Endothelial cells, G protein-coupled receptors, NF-kappaB

## Abstract

The lymphatic system maintains tissue fluid balance, and *FOXC2* mutations cause lymphoedema-distichiasis syndrome, which is characterized by lymphatic valve defects. Although oscillatory shear stress regulates FOXC2 expression, other extracellular regulators remain unclear. In this study, we identified LPA4 and LPA6, two Gα12/Gα13-coupled receptors for the bioactive lipid lysophosphatidic acid (LPA), as key regulators of FOXC2 expression and lymphatic valve development. Lymphatic endothelial cell–specific (LEC-specific) *Lpa4* *Lpa6*–deficient mice exhibited impaired lymphatic valve formation and maintenance, which resembled phenotypes of LEC-specific *Foxc2*-deficient mice, including abnormal lymphatic vessel patterning. Mechanistically, lymphatic endothelial *Lpa4*/*Lpa6* ablation reduced FOXC2 expression in vitro and in vivo. NF-κB was found to be essential for LPA-induced FOXC2 expression through the LPA4/LPA6-Gα12/Gα13-Rho kinase signaling axis. Accordingly, pharmacological inhibition of NF-κB and Rho kinase impaired lymphatic valve maintenance in mice. These results suggested that lymphatic endothelial LPA4 and LPA6 synergistically regulate FOXC2 expression through NF-κB activation and play an important role in lymphatic valve formation and maintenance. Our findings provide a molecular basis for lymphatic vessel development with a therapeutic potential for targeting lymphatic system–associated diseases.

## Abstract 2

The lipid mediator lysophosphatidic acid (LPA) activates Gα12/Gα13-coupled receptors in lymphatic endothelium and drives lymphatic valve development via NF-κB activation to promote FOXC2 expression.

## Graphical abstract

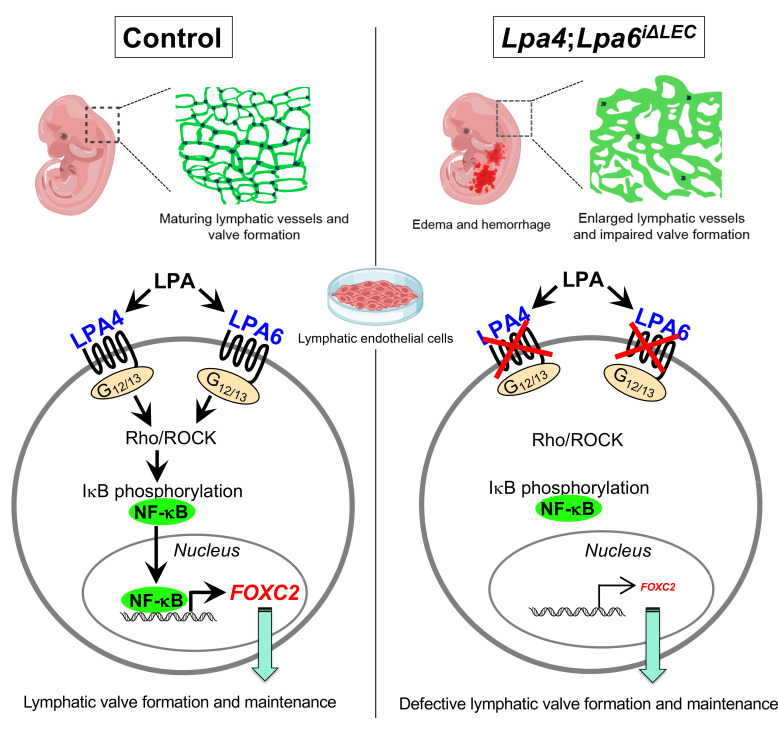

## Introduction

Lysophosphatidic acid (LPA) is a simple phospholipid consisting of a phosphate head group, glycerol moiety, and single fatty acid chain (1). It is produced extracellularly by 2 secreted enzymes, autotaxin and phosphatidic acid–selective phospholipase A1 (PA-PLA1α), which convert lysophospholipids, primarily lysophosphatidylcholine (LPC) and PA, respectively, to LPA (2). As a mixture of molecular species with various fatty acid compositions that are esterified at either the *sn*-1 or *sn*-2 position of the glycerol backbone, LPA is constitutively present in the blood and lymph (3). LPA binds to specific GPCRs, namely, LPA1–LPA6, and influences cell proliferation, migration, cytokine production, and angiogenesis (1, 4, 5). Among these receptors, LPA4 and LPA6, which we previously identified as novel LPA receptors (6, 7), efficiently couple with Gα12/Gα13 proteins and presumably share downstream intracellular signaling, including the activation of Rho and its effector Rho kinase (also known as ROCK) (7, 8). Furthermore, we revealed that LPA4 and LPA6 showcase distinct ligand selectivities for LPA species. Specifically, LPA with a fatty acid at the *sn*-1 position binds more potently to LPA4 than LPA6 (6, 7), whereas LPA6 preferentially responds to LPA with a fatty acid at the *sn*-2 position than at the *sn*-1 position (7). This coordinated sensing system by LPA4 and LPA6 plays a crucial role in blood endothelial cells (BECs) during developmental angiogenesis (9).

Lymphatic vessel formation begins after the onset of blood circulation during embryonic development (10–12). An important role of the lymphatic system is to collect excess interstitial fluid and return it to the blood stream. Its development involves a critical stage of valve formation in collecting lymphatic vessels (11, 13), which prevents backflow and ensures unidirectionality. In the absence of these valves, interstitial fluid accumulates in the tissues, resulting in severe edema (11). Oscillatory shear stress (OSS) is the frictional force exerted on lymphatic endothelial cells (LECs) by turbulent lymph flow. Such flow patterns are observed at the bifurcation of lymphatic vessels (14), where lymphatic valves are frequently formed (15). Therefore, OSS has been proposed as an upstream regulator of valvular endothelial cell (EC) differentiation (16). OSS-induced signaling induces the expression of forkhead box protein C2 (FOXC2) in LECs (13), which is essential for lymphatic valve development. Indeed, *Foxc2* deletion in mice causes loss of lymphatic valves in addition to severe morphological defects in the lymphatic vessels (17–21). However, the molecular mechanisms governing its expression in LECs remain incompletely understood.

The NF-κB family consists of RelA (p65), RelB, c-Rel, p105/p50, and p100/p52 (22). These transcription factors are involved in numerous immunological processes, including acute and chronic inflammatory responses. In the canonical NF-κB pathway, signal-induced phosphorylation of IκBα by IκB kinase (IKK) in the cytosol triggers IκBα degradation and subsequent release of RelA and p50 heterodimers, which translocate to the nucleus and induce target gene expression. Reportedly, NF-κB activation stimulates *VEGFR3* expression in LECs and promotes lymphangiogenesis during inflammation (23); however, its role in developmental lymphangiogenesis under physiological conditions remains unclear.

In this study, we revealed that LPA4 and LPA6 synergistically contribute to lymphatic valve development. LEC-specific ablation of *Lpa4* and *Lpa6* impaired lymphatic valve formation and maintenance in mouse embryos and neonates, respectively. Both ROCK and NF-κB inhibitors reduced lymphatic valve numbers in mouse neonates. In vitro, the LPA4/LPA6-Gα12/Gα13-ROCK signaling axis increased FOXC2 expression through NF-κB activation in LECs. Altogether, our findings suggested that the lymphatic endothelial LPA4/LPA6-Gα12/Gα13-ROCK-NF-κB signaling axis plays a pivotal role in lymphatic valve development by promoting FOXC2 expression.

## Results

### Mouse LECs express Lpa4 and Lpa6 mRNA at high levels.

**Figure 1:**
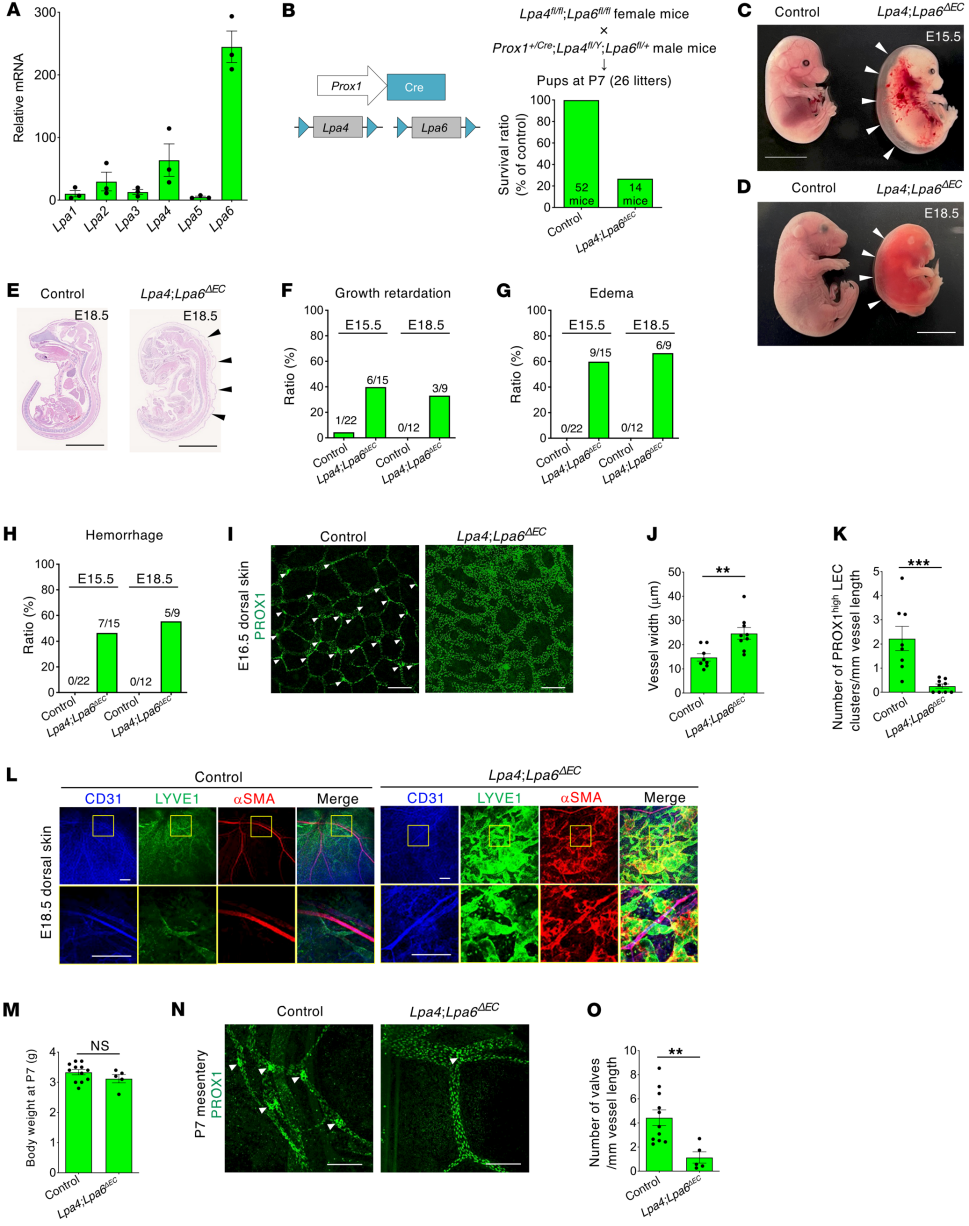
Mice deficient in lymphatic endothelial *Lpa4* and *Lpa6* exhibit severe edema, hemorrhage, and impaired lymphatic valve formation. (**A**) LPA receptor mRNA expression in mouse LECs detected via qRT-PCR (*n* = 3 independent LEC preparations from WT mice). (**B**) Impaired survival of *Lpa4* *Lpa6^ΔEC^* mice. Schematic diagram of *Lpa4* and *Lpa6* ablation in *Lpa4* *Lpa6^ΔEC^* mice is shown on the left. The numbers of mice at P7 are indicated in the bars. Detailed data are shown in Supplemental Figure 5B. Note that *Lpa4* is located on the X chromosome. (**C** and **D**) Growth retardation, severe edema (arrowheads), and hemorrhage observed in *Lpa4* *Lpa6^ΔEC^* littermate embryos at E15.5 (**C**) and E18.5 (**D**). Scale bars: 10 mm. (**E**) H&E-stained transverse sections of littermate embryos at E18.5, showing severe edema in *Lpa4* *Lpa6^ΔEC^* embryos (arrowheads). Scale bars: 10 mm. (**F**–**H**). Ratios of growth retardation (**F**), edema (**G**), and hemorrhage (**H**) in control and *Lpa4Lpa6^ΔEC^* embryos at E15.5 and E18.5. The numbers of affected embryos and total number of embryos analyzed are shown above each bar. (**I**) Representative confocal images of lymphatic vascular networks in the dorsal skin at E16.5, showing PROX1 immunostaining. White arrowheads indicate putative lymphatic valve-forming PROX1^hi^ LEC clusters. Scale bars: 200 μm. (**J** and **K**) Quantification of vessel width (**J**) and number of PROX1^hi^ LEC clusters (**K**) in control and *Lpa4* *Lpa6^ΔEC^* embryos (*n* = 8–9 embryos). (**L**) Representative confocal images of LYVE1^+^ lymphatic vessels covered with αSMA^+^ cells in the dorsal skin of control and *Lpa4* *Lpa6^ΔEC^* littermate embryos at E18.5 (*n* = 4–5 embryos). Areas in yellow boxes are magnified in the bottom panels. LECs display lower CD31 expression, while BECs express high CD31 levels. Scale bars: 200 μm. (**M**) Body weights of control and *Lpa4* *Lpa6^ΔEC^* mice at P7 (*n* = 5–12 mice). (**N**) Representative confocal images of mesenteric lymphatic vessels in control and *Lpa4* *Lpa6^ΔEC^* mice at P7, showing PROX1 immunostaining. White arrowheads indicate lymphatic valves. Scale bars: 200 μm. (**O**) Quantification of lymphatic valve numbers in control and *Lpa4* *Lpa6^ΔEC^* mice at P7 (*n* = 5–11 mice). **P < 0.01, ***P < 0.001; 2-tailed unpaired Student’s *t* test.

We previously revealed that endothelial LPA4 and LPA6 synergistically regulate blood vessel angiogenesis in mice (9). To elucidate their roles in lymphangiogenesis, we examined LPA receptor mRNA expression profiles in primary LECs isolated from mouse lungs (Supplemental Figure 1; supplemental material available online with this article; https://doi.org/10.1172/JCI193364DS1). LYVE1 and podoplanin (PDPN) immunostaining confirmed LEC identity and purity. We detected the mRNA expression of *Lpa4* and *Lpa6* in primary LECs (Figure 1A). Furthermore, although it was technically difficult to distinguish between valves and other regions within lymphatic vessels, in situ hybridization revealed the mRNA expression of *Lpa4* and *Lpa6* in VEGFR3^+^ lymphatic vessels of the dorsal skin at E18.5 (Supplemental Figure 2). These results suggested an LEC-intrinsic role of LPA4 and LPA6 in lymphatic vessel development.

### Lpa4/Lpa6 ablation in LECs impairs lymphatic plexus patterning and valve formation during embryonic development.

The mouse lymphatic system develops at E9.5, when PROX1^+^ LEC precursors (initial LECs) arise from a committed subpopulation of ECs in the cardinal vein (24). Until the lymph sac forms, initial LECs are continuously supplied by the cardinal veins (25). To study LPA4 and LPA6 function in lymphatic development, we generated a mouse line expressing constitutively active Cre recombinase in initial LECs. Using the CRISPR/Cas9 system, we inserted a P2A-Cre cDNA in-frame before the *Prox1* stop codon (26) (Supplemental Figure 3A). This modified *Prox1* allele (*Prox1-Cre*) enables bicistronic expression of PROX1 and Cre, thereby maintaining near-normal PROX1 levels (Supplemental Figure 4). Indeed, mice heterozygous for the modified *Prox1* allele (*Prox1^+/Cre^* mice) did not show any haploinsufficiency-related phenotypes observed in *Prox1^+/lacZ^* mice (Supplemental Figure 4) (24, 27).

We then generated *Prox1^+/Cre^* *Lpa4^fl/fl(Y)^* *Lpa6^fl/fl^* mice by crossing *Prox1^+/Cre^* *Lpa4^fl/Y^* *Lpa6^fl/+^* male mice with *Lpa4^fl/fl^* *Lpa6^fl/fl^* female mice (Supplemental Figure 5A). We found that *Lpa4*- and *Lpa6*-floxed alleles from the male mice were globally excised to convert them to KO alleles in the offspring (Supplemental Figure 5). A similar unexpected deletion pattern was observed in *Lpa4*- and *Lpa6*-floxed alleles from *Prox1^+/Cre^* *Lpa4^fl/fl^* *Lpa6^fl/+^* female mice (data not shown). These results indicated that Cre recombinase expressed by the *Prox1* promoter excised the floxed alleles in male and female germlines, which is consistent with another *Prox1* promoter-driven Cre mouse line that displayed a similar phenomenon (28). Lineage tracing using an *R26^+/tdTomato^* reporter mouse line revealed that Cre recombinase was active in LECs at E18.5 and P7 (Supplemental Figure 6, A and B). However, contrary to expectations, blood vessels were also clearly labeled with tdTomato, indicating that the *Prox1* promoter-driven Cre recombinase excised the floxed alleles in some populations of CD31^+^ BECs as well as LECs. Whether PROX1 expression is temporarily induced in EC precursors (i.e., angioblasts) before the appearance of initial LECs (25, 28, 29) remains to be elucidated.

The resulting *Prox1^+/Cre^* *Lpa4^fl/−(Y)^* *Lpa6^fl/−^* (hereafter referred to as *Lpa4* *Lpa6*^ΔEC^) mice, in which only 1 allele of each floxed *Lpa4* and *Lpa6* remained to be excised, were expected to exhibit effective depletion of expression of both genes in initial LECs by Cre. Allele-specific PCR analysis confirmed the excisions of floxed *Lpa4* and *Lpa6* alleles in the tails of *Prox1^+/Cre^* *Lpa4^fl/Y^* *Lpa6^fl/+^* mice at P7 (Supplemental Figure 3B). *Cre-*negative *Prox1^+/+^* *Lpa4^fl/−(Y)^* *Lpa6^fl/−^* littermates were used as control mice, as they did not show any apparent abnormality. Notably, the number of *Lpa4* *Lpa6*^ΔEC^ mice was approximately 75% lower than that of control mice at P7 (Figure 1B and Supplemental Figure 5B) and at 3 weeks old (Supplemental Figure 5C), suggesting that a significant proportion of *Lpa4* *Lpa6*^ΔEC^ mice were embryonically lethal. Consistent with prior observations in a subset of global *Lpa4*^−/−(Y)^ embryos (30), macroscopic inspection revealed that more than half of *Lpa4* *Lpa6*^ΔEC^ embryos exhibited various pathological manifestations, such as severe edema, hemorrhage, and growth retardation at E15.5 (Figure 1, C and F–H) and E18.5 (Figure 1, D–H). The extent of these pathologies varied greatly among individuals within the group. Furthermore, these mutant embryos displayed enlarged lymphatic vessels at E16.5 compared with controls (Figure 1, I and J), although no significant difference in branching patterns was observed (Supplemental Figure 7A). Abnormal coverage of α-smooth muscle actin–positive (αSMA^+^) cells on lymphatic vessels is a well-known phenotype observed in lymphedema in both humans and mice (17, 31). Dermal lymphatic vessels of *Lpa4* *Lpa6*^ΔEC^ embryos, especially those of edematous ones, were noticeably covered with αSMA^+^ cells at E18.5, whereas little to no αSMA^+^ cells were recruited to lymphatic vessels in control mice (Figure 1L).

The number of PROX1^hi^ LEC clusters, which are putative lymphatic valve-forming regions, was significantly reduced in the dorsal skin of *Lpa4* *Lpa6*^ΔEC^ embryos at E16.5 compared with control mice (Figure 1, I and K). Consistently, mesenteric lymph vessels of *Lpa4* *Lpa6*^ΔEC^ mice at P7, despite having normal body weights (Figure 1M), demonstrated a significant reduction in the lymphatic valve formation (Figure 1, N and O). No obvious defects in valve formation were observed in global *Lpa4^−/−^* or *Lpa6^−/−^* mice (Supplemental Figure 8). These results strongly suggested that the synergistic action of LPA4 and LPA6 is essential for lymphatic valve formation. In contrast to the relatively mild and variable pathological manifestations described above (e.g., edema, hemorrhage, and growth retardation), lymphatic valve dysplasia was observed in all *Lpa4* *Lpa6*^ΔEC^ mice to a greater extent, with low interindividual variability.

### Lymphatic endothelial LPA4/LPA6 are essential for maintaining lymphatic valves.

**Figure 2:**
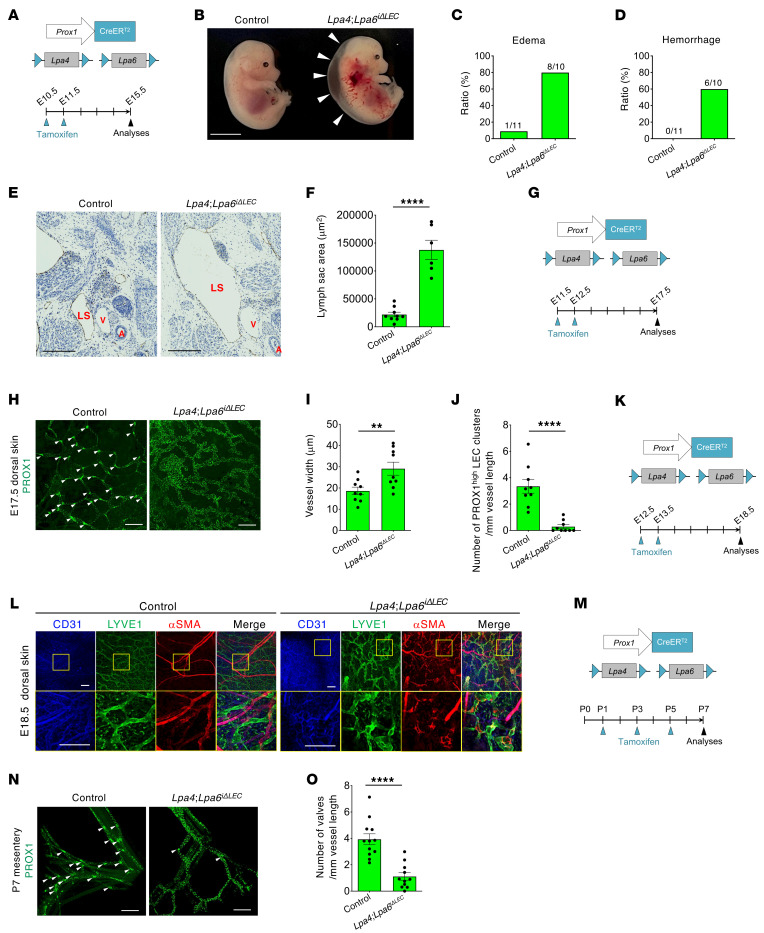
Lymphatic endothelial LPA4/LPA6 are essential for lymphatic valve formation and maintenance. (**A**) Schematic diagram of *Lpa4* and *Lpa6* ablation in *Lpa4* *Lpa6^iΔLEC^* mice and tamoxifen injection procedure for analysis at E15.5. (**B**) Gross morphology of control and *Lpa4* *Lpa6^iΔLEC^* littermate embryos, displaying severe edema (arrowheads) and hemorrhage. Scale bar: 10 mm. (**C** and **D**) Ratios of edema (**C**) and hemorrhage (**D**) in control and *Lpa4* *Lpa6^iΔLEC^* embryos, with the number of affected embryos and total numbers of embryos analyzed shown above each bar. (**E**) Representative hematoxylin-counterstained transverse sections of the jugular area in littermate embryos. LECs are immunostained for VEGFR3. Lymph sacs are remarkably enlarged in *Lpa4* *Lpa6^iΔLEC^* embryos compared with controls. LS, lymph sac; V, vein; A, aorta. Scale bars: 200 μm. (**F**) Quantification of lymph sac area (μm^2^) (*n* = 6–9 embryos). (**G**) Schematic diagram of *Lpa4* and *Lpa6* ablation in *Lpa4* *Lpa6^iΔLEC^* mice and tamoxifen injection procedure for analysis at E17.5. (**H**) Representative confocal images of dorsal skin lymphatic vascular networks, showcasing PROX1 immunostaining. White arrowheads indicate putative lymphatic valve–forming PROX1^hi^ LEC clusters. Scale bars: 200 μm. (**I** and **J**) Quantification of vessel width (**I**) and the number of PROX1^hi^ LEC clusters (**J**) in control and *Lpa4* *Lpa6^iΔLEC^* embryos (*n* = 9 embryos). (**K**) Schematic diagram of *Lpa4* and *Lpa6* ablation in *Lpa4* *Lpa6^iΔLEC^* mice and tamoxifen injection procedure for analysis at E18.5. (**L**) Representative confocal images of LYVE1^+^ lymphatic vessels covered with αSMA^+^ cells in the dorsal skin of control and *Lpa4* *Lpa6^iΔLEC^* littermate embryos (*n* = 4 embryos). Areas in yellow boxes are magnified in the bottom panels. Scale bars: 200 μm. (**M**) Schematic diagram of *Lpa4* and *Lpa6* ablation in *Lpa4* *Lpa6^iΔLEC^* mice and tamoxifen injection procedure for analysis at P7. (**N**) Representative confocal images of mesenteric lymphatic vessels in control and *Lpa4* *Lpa6^iΔLEC^* littermates, showing PROX1 immunostaining. White arrowheads indicate lymphatic valves. Scale bars: 200 μm. (**O**) Quantification of lymphatic valve number (*n* = 11–12 mice). **P < 0.01, ****P < 0.0001; 2-tailed unpaired Student’s *t* test.

To verify that the observed phenotypes were caused by LPA4/LPA6 depletion within the lymphatic vasculature, we generated a *Prox1^+/CreERT2^* mouse line, which selectively targets LECs in a time-controlled manner via tamoxifen administration, with a design similar to that of the *Prox1^+/Cre^* mouse line (Supplemental Figure 3, C and D). Lineage tracing using the *R26^+/tdTomato^* reporter mouse line confirmed Cre activity not only in LECs but also partially in BECs after tamoxifen administration to *Prox1^+/CreERT2^* embryos at E10.5 and E11.5 (Supplemental Figure 6, C and D). However, LEC-specific Cre activity was observed after tamoxifen administration at E12.5 or later (Supplemental Figure 6, E and F). We then crossed *Lpa4* *Lpa6* double-floxed mutants with *Prox1^+/CreERT2^* mice to obtain *Prox1^+/CreERT2^* *Lpa4^fl/fl(Y)^* *Lpa6^fl/fl^* (hereafter referred to as *Lpa4* *Lpa6*^i*Δ*LEC^) mice. *CreER^T2^*-negative *Prox1^+/+^* *Lpa4^fl/fl(Y)^* *Lpa6^fl/fl^* littermates were used as controls. When tamoxifen was administered to pregnant females at E10.5 and E11.5 (Figure 2A), a substantial fraction of the *Lpa4* *Lpa6*^i*Δ*LEC^ embryos displayed severe edema, hemorrhage, and enlarged lymph sacs at E15.5 (Figure 2, B–F). When administered either at E11.5 and E12.5 or E12.5 and E13.5 (Figure 2, G and K), no edema or hemorrhage was observed in *Lpa4* *Lpa6*^i*Δ*LEC^ embryos at E17.5 or E18.5, respectively. Despite this, *Lpa4* *Lpa6*^i*Δ*LEC^ embryos at E17.5 suffered from valve defects in the skin (Figure 2, H–J, and Supplemental Figure 7B), where valve formation initiates at around E15.5 (14). In addition, the dermal lymphatic vessels of *Lpa4* *Lpa6*^i*Δ*LEC^ embryos at E18.5 were heavily covered with αSMA^+^ cells compared with negative control littermates (Figure 2L). Furthermore, we examined the morphology of lymphovenous valves (LVVs), which prevent venous blood from flowing backward into the lymphatic vessels (32). The LVV is formed in 3 development stages in LECs: delamination (E12.0), aggregation (E12.5), and maturation (E14.5–E16.5). Both tamoxifen administrations starting at E10.5 (Supplemental Figure 9, A and B) and E12.5 (Supplemental Figure 9, C and D) caused LVV defects in *Lpa4* *Lpa6*^i*Δ*LEC^ embryos.

The contribution of LPA4/LPA6 in lymphangiogenesis is unclear. Therefore, we examined dermal lymphatic sprouting parameters, including filopodia number, branch point density, vessel width, and VEGFR3 expression, in capillary lymphatic vessels in embryos at E16.5. The data showed that these parameters were comparable between *Lpa4* *Lpa6*^i*Δ*LEC^ and control embryos (Supplemental Figure 10). These findings suggested that lymphatic endothelial LPA4/LPA6 are not essential for lymphangiogenesis, ruling out the possibility that the valve defect was caused secondarily by the upstream abnormalities in the capillary plexus.

To investigate the roles of lymphatic endothelial LPA4/LPA6 in postnatal valve maintenance, we administered several doses of tamoxifen to control and *Lpa4* *Lpa6*^i*Δ*LEC^ pups starting at P1 (Figure 2M). *Lpa4* *Lpa6*^i*Δ*LEC^ mice at P7 had significantly fewer lymphatic valves than control littermates (Figure 2, N and O). Furthermore, impaired valve maintenance was also observed in lymphatic vessels of the ears of *Lpa4* *Lpa6*^i*Δ*LEC^ mice at 6 weeks of age (Supplemental Figure 11). To determine whether loss of LPA4/LPA6 in LECs affects functional lymphatic drainage, we examined lymphatic transport by intradermal injection of FITC-dextran into hind limb footpads of 6-week-old mice. *Lpa4* *Lpa6*^i*Δ*LEC^ mice had decreased lymphatic draining efficiency compared with control littermates (Supplemental Figure 12). These results suggested that LPA4/LPA6 signaling in LECs is critical for valve maintenance. Nevertheless, these *Lpa4* *Lpa6*^i*Δ*LEC^ mice survived without apparent abnormalities until at least 8 weeks of age, and no chylous effusion was noted (*n* = 8 mice).

### Lymphatic endothelial LPA4/LPA6 activate the Gα12/Gα13-ROCK signaling pathway.

**Figure 3:**
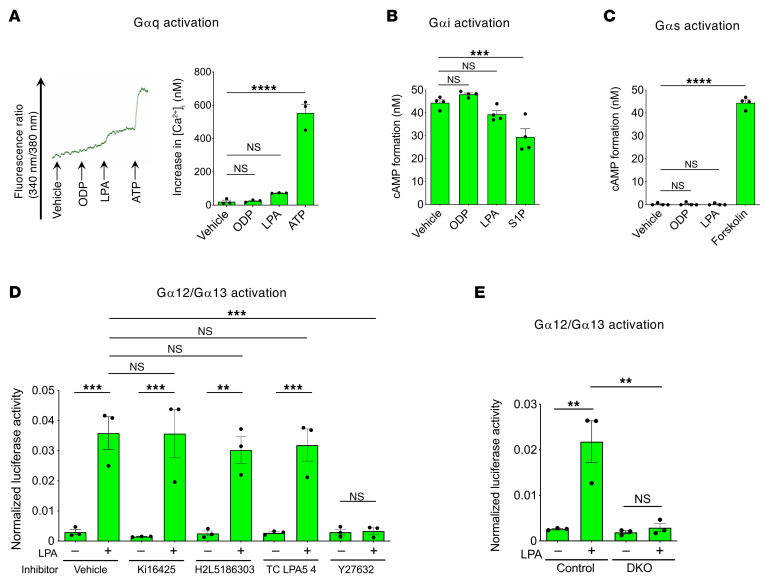
LPA4/LPA6 mediate LPA-induced Gα12/Gα13 activation in LECs. (**A**) Intracellular calcium influx assay to detect Gαq activation. Representative trace of changes in intracellular Ca^2+^ concentration ([Ca^2+^]_i_) is shown at the left. Mouse lung LECs were unresponsive to LPA (10 μM) and ODP (10 μM). ATP (10 μM) was used as a positive control. (**B**) cAMP assay to detect Gαi activation. No inhibition of forskolin-stimulated (20 μM) cAMP accumulation was observed in mouse lung LECs treated with LPA (10 μM) or ODP (10 μM). S1P (100 nM) was used as a positive control. (**C**) cAMP assay to detect Gαs activation. No significant increase in cAMP levels was observed in mouse lung LECs treated with LPA (10 μM) or ODP (10 μM). Forskolin (20 μM) was used as a positive control. (**D**) SRF-RE-Luc reporter assay to detect Gα12/Gα13-Rho activation. LPA (10 μM, 6 hours) increased reporter activity in serum-starved mouse lung LECs, which was attenuated by Y27632 (10 μM, 1-hour pretreatment) but not by the LPA1/LPA3 antagonist Ki16425, LPA2 antagonist H2L5186303, or LPA5 antagonist TC LPA5 4 (10 μM each, 1-hour pretreatment). (**E**) LPA4/LPA6-dependent SRF-RE-Luc reporter activity. The increased activity in response to LPA (10 μM, 6 hours) was attenuated in serum-starved mouse lung DKO LECs. Data are presented as mean ± SEM of triplicates. **P < 0.01, ***P < 0.001, ****P < 0.0001; 1-way ANOVA followed by Dunnett’s test (**A**–**C**) and Tukey’s multiple-comparison test (**D** and **E**).

The molecular mechanisms underlying LPA-induced lymphatic valve formation and maintenance remain largely unknown. Mouse lung LECs endogenously expressed mRNAs for *Lpa1*–*Lpa6* at variable levels (Figure 1A). To determine whether cultured mouse lung LECs respond to LPA, we assessed calcium influx (Figure 3A) and intracellular cAMP level changes (Figure 3, B and C), which are mediated by Gαq, Gαi, and Gαs proteins. Neither LPA nor octadecenyl phosphate (ODP), an agonist of LPA4, LPA5, and LPA6 (33), significantly altered cAMP levels, whereas LPA but not ODP, induced a slight nonsignificant calcium influx, presumably owing to low-level activation of LPA1–3. Therefore, it is unlikely that LPA4 and LPA6 couple to these Gα proteins in LECs. Next, we performed a serum response factor-response element (SRF-RE) luciferase reporter assay to detect Gα12/Gα13-Rho-ROCK signaling activation (9, 34). LPA significantly increased reporter activity in mouse lung LECs, which was suppressed by the ROCK inhibitor Y27632 but unaffected by the LPA1/LPA3 antagonist Ki16425, LPA2 antagonist H2L5186303, or LPA5 antagonist TC LPA5 4 (Figure 3D). Notably, *Lpa4*/*Lpa6*-deficient LECs (DKO LECs) from *Lpa4* *Lpa6*^ΔEC^ mice were unresponsive to LPA (Figure 3E). Unlike mouse lung LECs, all human LECs we examined — dermal LECs (HDLECs and HMVECs-dNeo) and lung LECs (HMVECs-L) — predominantly expressed *LPA6* mRNA (Supplemental Figure 13, A–C). Similar to mouse lung LECs, these human LECs demonstrated increased reporter activity in response to LPA, and as expected, these increases were inhibited by *LPA6* siRNA treatment (Supplemental Figure 13D). These results indicated that LPA activates the Gα12/Gα13-ROCK signaling pathway through LPA4/LPA6 in LECs.

### Lpa4/Lpa6 ablation decreases nuclear FOXC2 expression in LECs.

**Figure 4:**
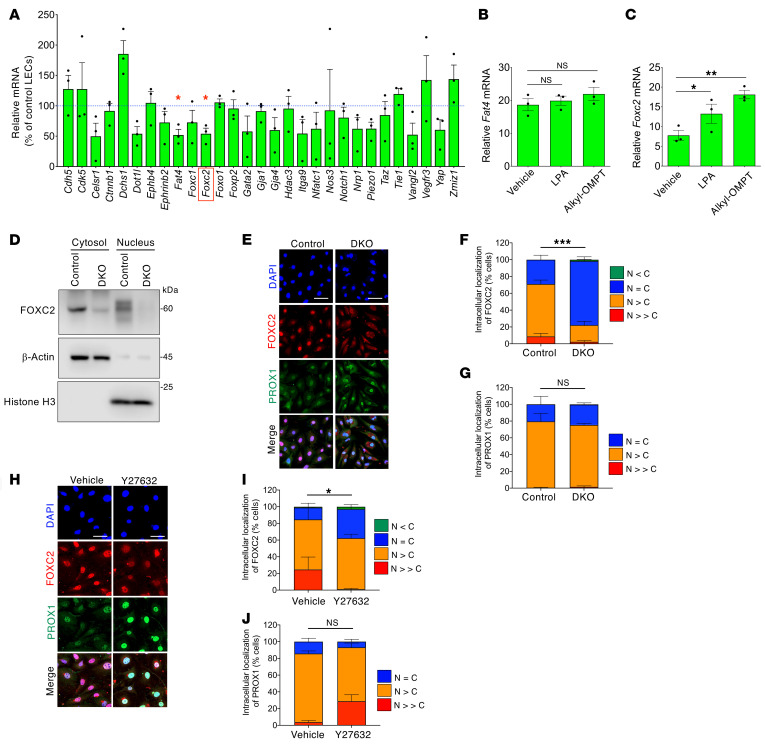
*Lpa4*/*Lpa6* deletion decreases FOXC2 expression in LECs. (**A**) Screening for genes associated with lymphatic valve formation in mouse lung LECs affected by *Lpa4*/*Lpa6* deletion. Gene expression level in LECs from *Lpa4* *Lpa6^ΔEC^* mice is normalized to that of control mice. Data are presented as mean ± SEM (*n* = 3 sets of independent LECs per group prepared in parallel). *P < 0.05; 2-tailed Welch’s *t* test. (**B**) *Fat4* mRNA expression unresponsive to LPA (10 μM, 3 hours) and alkyl-OMPT (10 μM, 3 hours) in serum-starved mouse lung LECs. One-way ANOVA followed by Dunnett’s test. (**C**) Increased *Foxc2* mRNA expression in response to LPA (10 μM, 3 hours) and alkyl-OMPT (10 μM, 3 hours) in serum-starved mouse lung LECs. Data are presented as mean ± SEM of triplicates. *P < 0.05, **P < 0.01; 1-way ANOVA followed by Dunnett’s test. (**D**) Reduced FOXC2 protein expression in cytosolic and nuclear fractions isolated from lung DKO LECs. Unprocessed Western blot scans are shown in Supplemental Figure 26. (**E**–**G**) Reduced FOXC2 nuclear localization in lung DKO LECs. Representative confocal images (**E**) and corresponding quantification of FOXC2 (**F**) and PROX1 (**G**) intracellular localization (*n* = 201–365 cells). Scale bars: 100 μm. ***P < 0.001; 2-tailed unpaired Student’s *t* test for *N = C* ratio. (**H**–**J**) Reduced FOXC2 nuclear localization by Y27632 (10 μM, 6 hours) in mouse lung LECs. Representative confocal images (**H**) and corresponding quantification of FOXC2 (**I**) and PROX1 (**J**) intracellular localization (*n* = 208–332 cells). Scale bars: 50 μm. *P < 0.05; 2-tailed unpaired Student’s *t* test for *N = C* ratio.

Next, we sought to identify the genes responsible for LPA4/LPA6-mediated lymphatic valve formation and maintenance. qRT-PCR of cultured lung LECs from control and *Lpa4* *Lpa6*^ΔEC^ mice was performed to screen genes associated with lymphatic valve formation. The results showed that *Lpa4*/*Lpa6* deletion significantly downregulated *Fat4* and *Foxc2* expression in DKO LECs compared with control LECs (Figure 4A). In WT mouse LECs, *Fat4* mRNA expression remained unchanged following treatment with LPA and alkyl-OMPT, a metabolically stabilized LPA4 and LPA6 agonist (35, 36) (Figure 4B), while *Foxc2* mRNA expression was significantly upregulated (Figure 4C). In freshly isolated LECs, mRNA expression of *Foxc2* but not other valve-related genes, including *Fat4*, was reduced by *Lpa4*/*Lpa6* ablation (Supplemental Figure 14). Among LEC-specific KO mouse models, *Foxc2*-deficient mice harbored phenotypes similar to *Lpa4* *Lpa6*^i*Δ*LEC^ mice, including dilated lymphatic vessels with excessive αSMA^+^ cell coverage (19, 20) and defects in lymphatic valve formation and maintenance (19, 37). Thus, we focused on FOXC2, a transcription factor critical for the development and function of the lymphatic system, particularly in valve-bearing collecting lymphatic vessels (16–19). Consistent with the qRT-PCR analysis, Western blot analysis confirmed that *Lpa4/Lpa6* deletion reduced FOXC2 protein expression in cultured mouse lung LECs, especially in the nucleus (Figure 4D). Immunofluorescence analysis further showed a significant decrease in nuclear FOXC2 in DKO LECs compared with that in control LECs (Figure 4, E and F). In contrast, PROX1, another transcription factor highly expressed in valve LECs, showed no change in nuclear expression levels (Figure 4, E and G), suggesting that *Lpa4*/*Lpa6* deficiency had a specific inhibitory effect on nuclear accumulation of FOXC2. Accordingly, the ROCK inhibitor Y27632 treatment reduced nuclear FOXC2 but not PROX1 expression in WT LECs (Figure 4, H–J).

**Figure 5:**
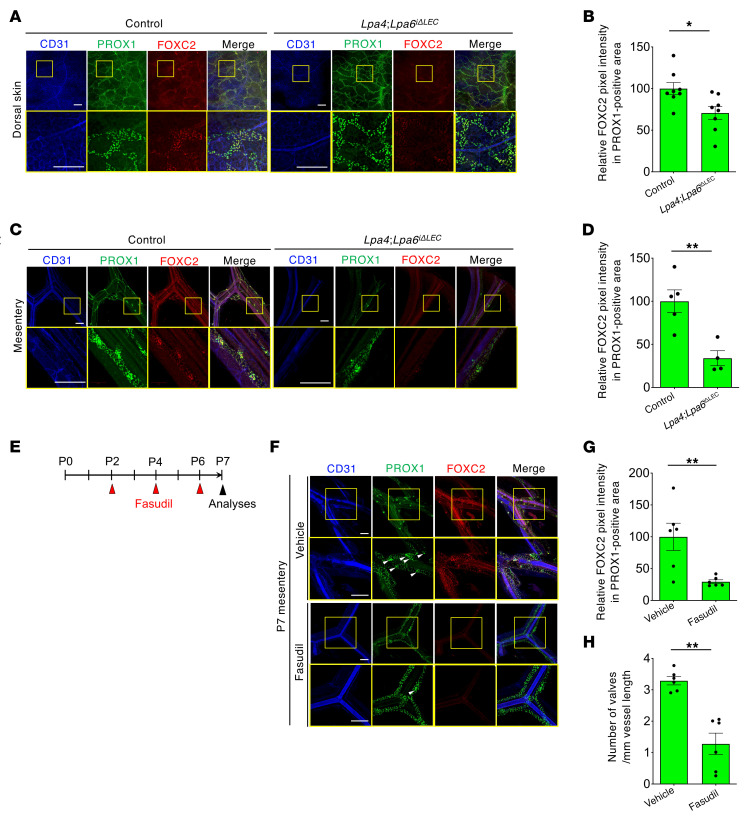
LEC-specific *Lpa4*/*Lpa6* deletion reduces FOXC2 expression in lymphatic vessels of mouse dorsal skin and mesentery. (**A**) Representative confocal images of lymphatic vascular networks of the dorsal skin at E17.5. Tamoxifen was injected as described in Figure 2G. Triple immunostaining for CD31, PROX1, and FOXC2 is shown. Areas in yellow boxes are magnified in the bottom panels. Scale bars: 200 μm. (**B**) Quantification of FOXC2 expression in the PROX1^+^ area at E17.5 (*n* = 8 embryos). *P < 0.05; 2-tailed unpaired Student’s *t* test. (**C**) Representative confocal images of mesenteric lymphatic vessels at P7. Tamoxifen was injected as described in Figure 2M. Triple immunostaining for CD31, PROX1, and FOXC2 is shown. Areas in yellow boxes are magnified in the bottom panels. Scale bars: 200 μm. (**D**) Quantification of FOXC2 expression in the PROX1^+^ area at P7 (*n* = 4–5 mice). *P* < 0.01; 2-tailed unpaired Student’s *t* test. (**E**) Schematic diagram of fasudil (50 mg/kg) oral administration to WT neonates analyzed at P7. (**F**) Representative confocal images of mesenteric lymphatic vessels in WT mice treated with fasudil. Triple immunostaining for CD31, PROX1, and FOXC2 is shown. Areas in yellow boxes are magnified in the bottom panels. White arrowheads indicate lymphatic valves. Scale bars: 200 μm. (**G** and **H**) Quantification of FOXC2 expression in the PROX1^+^ area (**G**) and lymphatic valve numbers (**H**) (*n* = 6 mice). **P < 0.01; 2-tailed unpaired Student’s *t* test. Confocal images of all samples are shown in Supplemental Figure 29.

Consistent with these in vitro data, FOXC2 protein expression levels were significantly reduced in dorsal skin at E17.5 (Figure 5, A and B) and mesenteric lymphatic vessels at P7 (Figure 5, C and D) in *Lpa4* *Lpa6*^i*Δ*LEC^ mice compared with those of control mice. *Lpa4* *Lpa6*^ΔEC^ also showed reduced FOXC2 expression in dorsal skin (Supplemental Figure 15, A and B) and mesenteric (Supplemental Figure 15, C and D) lymphatic vessels. Next, we examined the involvement of ROCK in FOXC2 expression in vivo using fasudil, a clinically used ROCK inhibitor (38) (Figure 5E). Administration of fasudil to WT neonatal mice significantly reduced FOXC2 expression (Figure 5, F and G) and the valve number (Figure 5, F and H) in the mesenteric lymphatic vessels. These results strongly suggested that LPA4/LPA6-mediated activation of ROCK in LECs is required for lymphatic valve maintenance.

### LPA increases nuclear FOXC2 expression in LECs through the LPA4/LPA6-Gα12/Gα13-ROCK signaling pathway.

**Figure 6:**
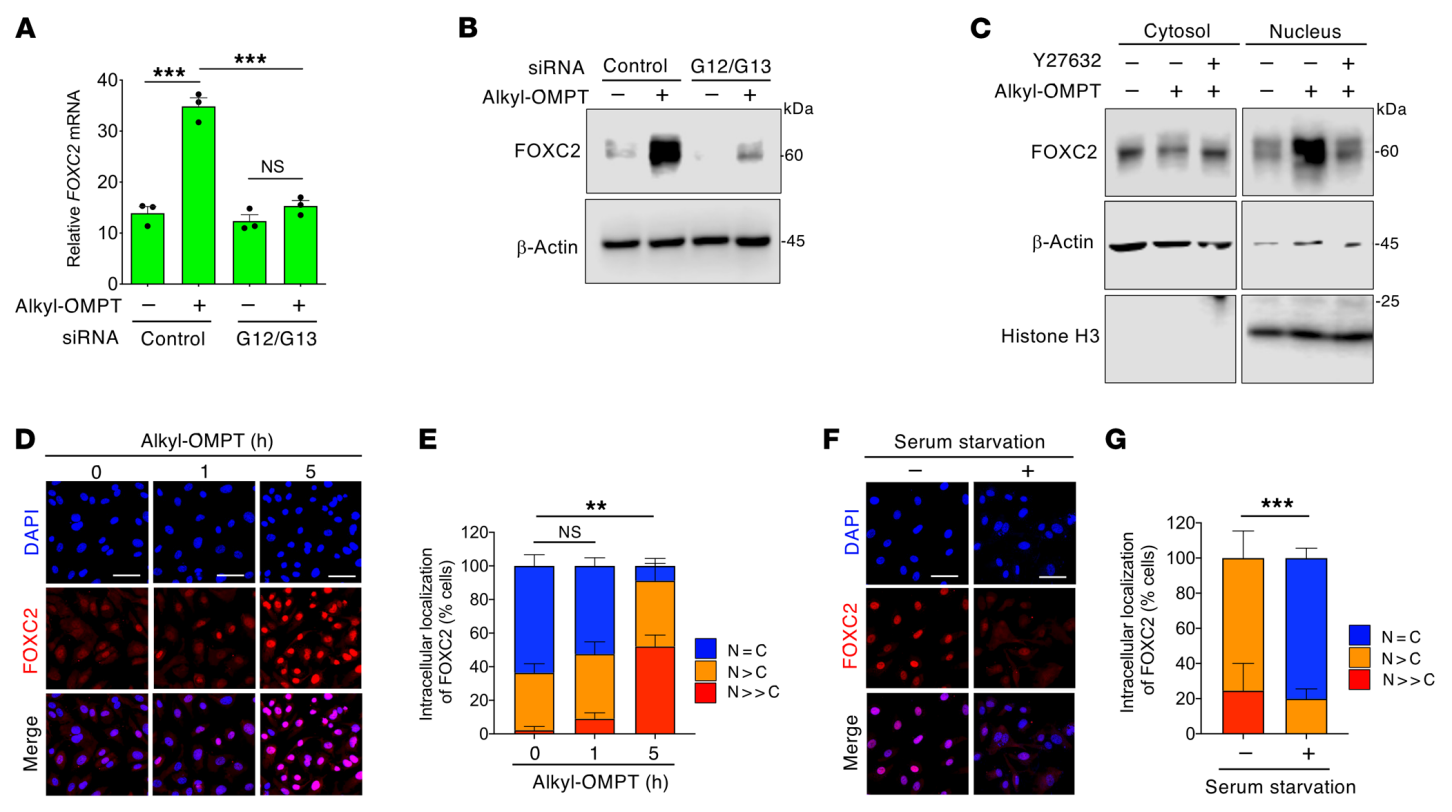
LPA4/LPA6 activation increases FOXC2 nuclear expression in LECs. (**A**) Increased *FOXC2* mRNA expression in response to alkyl-OMPT (10 μM, 3 hours) was blocked by *GNA12/GNA13* siRNAs (48-hour pretreatment) in serum-starved HMVECs-dNeo. Data are presented as mean ± SEM of triplicates. ***P < 0.001; 1-way ANOVA followed by Tukey’s multiple-comparison test. (**B**) Increased FOXC2 protein expression in response to alkyl-OMPT (10 μM, 6 hours) was blocked by *GNA12/GNA13* siRNAs (48-hour pretreatment) in serum-starved HMVECs-dNeo. (**C**) Increased FOXC2 nuclear expression in response to alkyl-OMPT (10 μM, 6 hours) was blocked by Y27632 (10 μM, 1-hour pretreatment) in serum-starved HMVECs-dNeo. (**D** and **E**) Time-dependent FOXC2 nuclear expression in serum-starved mouse lung LECs following alkyl-OMPT (10 μM) treatment. Representative confocal images (**D**) and corresponding quantification of FOXC2 intracellular localization (**E**) (*n* = 110–150 cells). Scale bars: 100 μm. **P < 0.01; 1-way ANOVA followed by Dunnett’s test for *N = C* ratio. (**F** and **G**) Reduced FOXC2 nuclear localization following 8-hour serum starvation in mouse lung LECs. Representative confocal images (**F**) and corresponding quantification of FOXC2 intracellular localization (**G**) (*n* = 62–76 cells). Scale bars: 100 μm. ***P < 0.001; 2-tailed Welch’s *t* test for *N = C* ratio. Unprocessed original scans of Western blots are shown in Supplemental Figure 26.

To confirm the ligand-dependent regulation of FOXC2 expression by LPA4/LPA6 signaling, we examined whether alkyl-OMPT positively affects FOXC2 expression in human dermal LECs. Indeed, alkyl-OMPT increased FOXC2 mRNA and protein expression levels, which were suppressed by siRNA-dependent knockdown of Gα12/Gα13 (Figure 6, A and B). Furthermore, alkyl-OMPT induced FOXC2 expression in the nucleus but not in the cytosol in a Y27632-sensitive manner (Figure 6C). Immunofluorescence analysis detected the nuclear accumulation of FOXC2 in response to alkyl-OMPT in a time-dependent manner (Figure 6, D and E). Removal of serum, which is abundant with LPA, markedly reduced nuclear FOXC2 accumulation (Figure 6, F and G). Taken together, these results supported the hypothesis that the ligand-dependent lymphatic endothelial LPA4/LPA6-Gα12/Gα13-ROCK signaling activation increases nuclear FOXC2 expression.

### LPA4/LPA6 activation has little involvement in VEGFR3 signaling.

VEGFR3 signaling was involved in FOXC2 expression in LECs (39) and lymphatic valve formation (40). Therefore, we analyzed whether LPA4/LPA6 signaling induces VEGFR3 expression. Human LECs were stimulated with alkyl-OMPT; however, this did not affect VEGFR3 mRNA expression levels (Supplemental Figure 16A). Additionally, the VEGFR3 immunohistochemical staining levels in the fetal skin lymphatic vessels of *Lpa4* *Lpa6*^i*Δ*LEC^ mice were not significantly different from those in control mice (Supplemental Figure 16, B–D); *Vegfr3* mRNA levels in DKO LECs also did not differ from those in control LECs (Figure 4A). VEGF-C/VEGFR3 signaling enhanced YAP and TAZ activity in LECs (41). Indeed, we observed that VEGF-C induced the expression of the *F3* gene, which encodes a tissue factor, in a manner sensitive to the YAP/TAZ inhibitor verteporfin but not to the ROCK inhibitor Y27632 (Supplemental Figure 16E). However, LPA4/LPA6 signaling induced *F3* gene expression in a ROCK- and YAP/TAZ-dependent manner. These results suggested that LPA signaling has little effect on VEGFR3 expression and signaling and that VEGFR3 functions independently of LPA signaling during embryonic development.

### LPA4/LPA6-induced NF-κB activation in LECs is mediated through ROCK.

**Figure 7:**
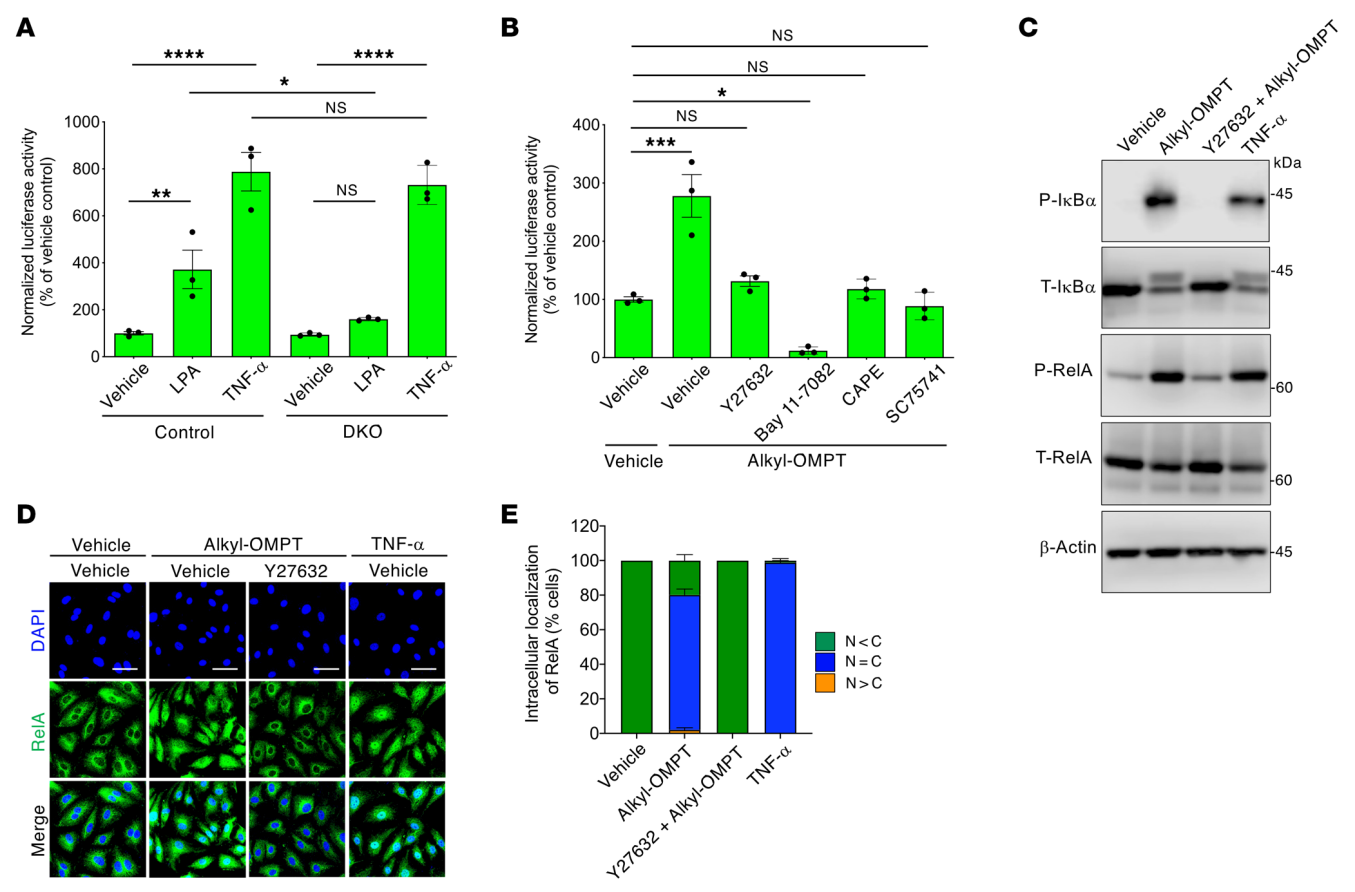
LPA4/LPA6 signaling activates NF-κB through ROCK in LECs. (**A**) Increased NF-κB reporter activity in response to LPA (10 μM, 6 hours) was attenuated by *Lpa4*/*Lpa6* deletion in serum-starved mouse lung LECs. Increase in the NF-κB reporter activity by TNF-α (50 ng/mL, 6 hours, positive control) remained unaffected by *Lpa4*/*Lpa6* deletion. Data are presented as mean ± SEM of triplicates. *P < 0.05, **P < 0.01, ****P < 0.0001; 1-way ANOVA followed by Tukey’s multiple-comparison test. (**B**) Increased NF-κB reporter activity in response to alkyl-OMPT (10 μM, 6 hours) was attenuated by Y27632 (10 μM, 1-hour pretreatment), Bay 11-7082 (5 μM, 1-hour pretreatment), CAPE (30 μM, 1-hour pretreatment), and SC75741 (10 μM, 1-hour pretreatment) in serum-starved HMVECs-dNeo. Data are presented as mean ± SEM of triplicates. *P < 0.05, ***P < 0.001; 1-way ANOVA followed by Dunnett’s test. (**C**) Phosphorylation of IκBα and RelA induced by alkyl-OMPT (10 μM, 30 minutes) was blocked by Y27632 (10 μM, 1-hour pretreatment) in serum-starved HMVECs-dNeo. Immunoblotting was performed using phosphorylated (P) and total (T) primary antibodies. TNF-α (50 ng/mL, 30 minutes) was used as a positive control. Unprocessed Western blot scans are shown in Supplemental Figure 27. (**D** and **E**) Nuclear translocation of RelA in response to alkyl-OMPT (10 μM, 1 hour) was blocked by Y27632 (10 μM, 1-hour pretreatment) in serum-starved HMVECs-dNeo. TNF-α (50 ng/mL, 1 hour) was used as a positive control. Representative confocal images (**D**) and corresponding quantification of RelA intracellular localization (**E**) (*n* = 76–101 cells). Scale bars: 100 μm.

LPA as well as thrombin, a ligand for PAR1 that couples to Gα12/Gα13 proteins (42), activates NF-κB in a Rho/ROCK-dependent manner in BECs (43–45). Thus, we investigated whether LPA induces NF-κB activation via LPA4/LPA6 in LECs. The NF-κB luciferase reporter assay revealed that, like TNF-α, LPA increased reporter activity in lung LECs of control mice, which was abolished by *Lpa4*/*Lpa6* deletion (Figure 7A). Similarly, human dermal LECs displayed increased NF-κB luciferase reporter activity in response to alkyl-OMPT. This activity was suppressed by treatment with Y27632 and the NF-κB signaling inhibitors with different chemical structures (Figure 7B), including Bay 11-7082 (46) and caffeic acid phenethyl ester (CAPE) (47), which prevent the nuclear translocation of the NF-κB component RelA (Supplemental Figure 17), and SC75741 (48), which inhibits RelA DNA binding but not nuclear translocation (Supplemental Figure 17). Western blot analysis showed that alkyl-OMPT treatment of human dermal LECs induced IκB and RelA phosphorylation to the same extent as that of TNF-α, both of which were inhibited by Y27632 (Figure 7C). Although in vivo RelA localization analysis was not possible in mice due to our technical limitations, nuclear accumulation of RelA in human dermal LECs was enhanced in vitro by alkyl-OMPT stimulation, which was also blocked by Y27632 (Figure 7, D and E). Together, these results demonstrated that LPA4/LPA6 activation induces NF-κB signaling through ROCK in LECs.

### NF-κB activation increases FOXC2 expression in LECs and is required for lymphatic valve maintenance.

**Figure 8:**
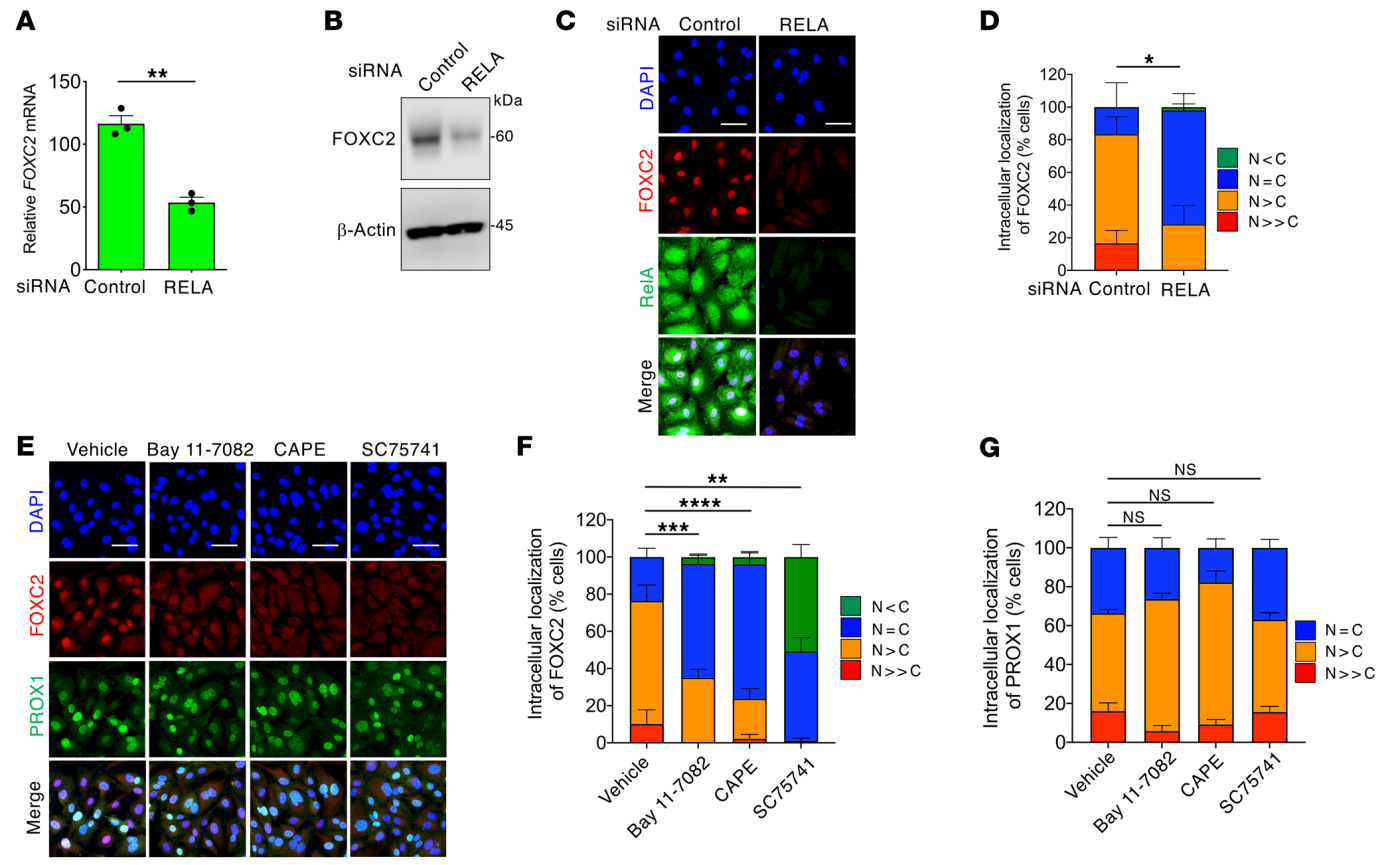
Impaired NF-κB signaling reduces nuclear FOXC2 expression in LECs. (**A** and **B**) Reduced expression of *FOXC2* mRNA (**A**) and protein (**B**) by *RELA* siRNA (48-hour pretreatment) in HMVECs-dNeo. Data are presented as mean ± SEM of triplicates. **P < 0.01; 2-tailed unpaired Student’s *t* test. Unprocessed original scans of Western blots are shown in Supplemental Figure 27. (**C** and **D**) Reduced FOXC2 nuclear localization by *RELA* siRNA (48-hour pretreatment) in HMVECs-dNeo. Representative confocal images (**C**) and corresponding quantification of FOXC2 intracellular localization (**D**) (*n* = 48–68 cells). Scale bars: 100 μm. *P < 0.05; 2-tailed unpaired Student’s *t* test for *N = C* ratio. (**E**–**G**) FOXC2 expression suppressed by NF-κB signaling inhibitors Bay 11-7082 (5 μM, 5 hours), CAPE (30 μM, 5 hours), and SC75741 (10 μM, 5 hours) in HMVECs-dNeo. Representative confocal images (**E**) and corresponding quantification of FOXC2 (**F**) and PROX1 (**G**) intracellular localization (*n* = 87–133 cells). Scale bars: 100 μm. **P < 0.01, ***P < 0.001, ****P < 0.0001; 1-way ANOVA followed by Dunnett’s multiple-comparison test for *N = C* ratio.

**Figure 9:**
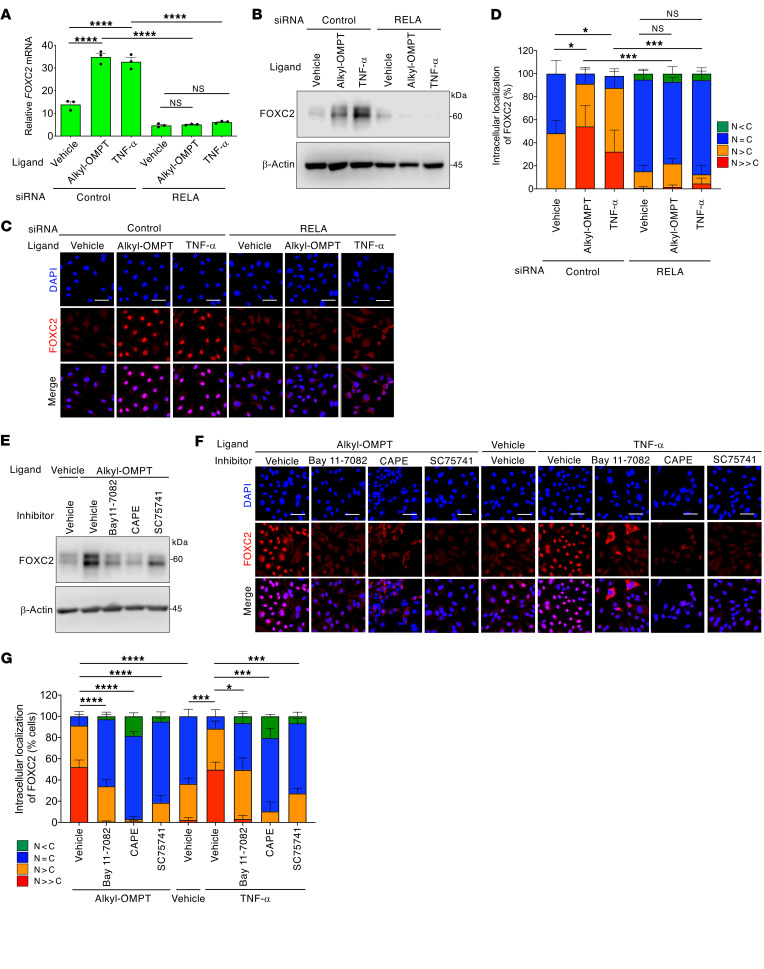
LPA4/LPA6 signaling-induced NF-κB activation promotes FOXC2 expression in LECs. (**A**) *FOXC2* mRNA induction in response to alkyl-OMPT (10 μM, 3 hours) or TNF-α (50 ng/mL, 3 hours) was suppressed by *RELA* siRNA (48-hour pretreatment) in serum-starved HMVECs-dNeo. Data are presented as mean ± SEM of triplicates. ****P < 0.0001; 1-way ANOVA followed by Tukey’s multiple-comparison test. (**B**) FOXC2 protein induction in response to alkyl-OMPT (10 μM, 6 hours) or TNF-α (50 ng/mL, 6 hours) was suppressed by *RELA* siRNA (48-hour pretreatment) in serum-starved HMVECs-dNeo. (**C** and **D**) FOXC2 nuclear expression in response to alkyl-OMPT (10 μM, 5 hours) or TNF-α (50 ng/mL, 5 hours) was suppressed by *RELA* siRNA (48-hour pretreatment) in serum-starved HMVECs-dNeo. Representative confocal images (**C**) and corresponding quantification of FOXC2 intracellular localization (**D**) (*n* = 61–92 cells). Scale bars: 100 μm. *P < 0.05, ***P < 0.001; 1-way ANOVA followed by Tukey’s multiple-comparison test for *N = C* ratio. (**E**) FOXC2 protein induction in response to alkyl-OMPT (10 μM, 6 hours) was suppressed by Bay 11-7082 (5 μM), CAPE (30 μM), and SC75741 (10 μM) pretreated for 1 hour in serum-starved HMVECs-dNeo. (**F** and **G**) FOXC2 nuclear expression in response to alkyl-OMPT (10 μM, 5 hours) or TNF-α (50 ng/mL, 5 hours) was suppressed by Bay 11-7082 (5 μM), CAPE (30 μM), and SC75741 (10 μM) pretreated for 1 hour in serum-starved HMVECs-dNeo. Representative confocal images (**F**) and corresponding quantification of FOXC2 intracellular localization (**G**) (*n* = 78–202 cells). Scale bars: 100 μm. *P < 0.05, ***P < 0.001, ****P < 0.0001; 1-way ANOVA followed by Dunnett’s test for *N = C* ratio (vs. *Alkyl-OMPT + Vehicle* or *TNF-α + Vehicle* cells). Unprocessed original scans of Western blots are shown in Supplemental Figure 27.

To confirm the involvement of NF-κB in FOXC2 expression within human dermal LECs, we utilized siRNA to knock down RelA, encoded by *RELA*. Indeed, *RELA* siRNA drastically reduced the expression levels of *FOXC2* mRNA (Figure 8A) and FOXC2 protein (Figure 8B), which predominantly localized to the nucleus (Figure 8, C and D). Consistent reduction was observed in LECs treated with 3 NF-κB signaling inhibitors (Figure 8, E and F), whereas none of which affected PROX1 nuclear localization (Figure 8, E and G), probably ruling out nonspecific inhibitory effects of these inhibitors on nuclear accumulation of transcription factors. TNF-α, unlike alkyl-OMPT, potently induced the FOXC2 expression independently of Gα12/Gα13 (Supplemental Figure 18, A and B) and ROCK (Supplemental Figure 18, C–E). RelA knockdown inhibited FOXC2 induction by alkyl-OMPT and TNF-α (Figure 9, A–D). Pharmacological inhibition of NF-κB also prevented FOXC2 induction by alkyl-OMPT and TNF-α (Figure 9, E–G). These findings supported the proposal that NF-κB, which is activated downstream of the LPA4/LPA6-Gα12/Gα13-ROCK signaling, regulates FOXC2 expression in LECs.

**Figure 10:**
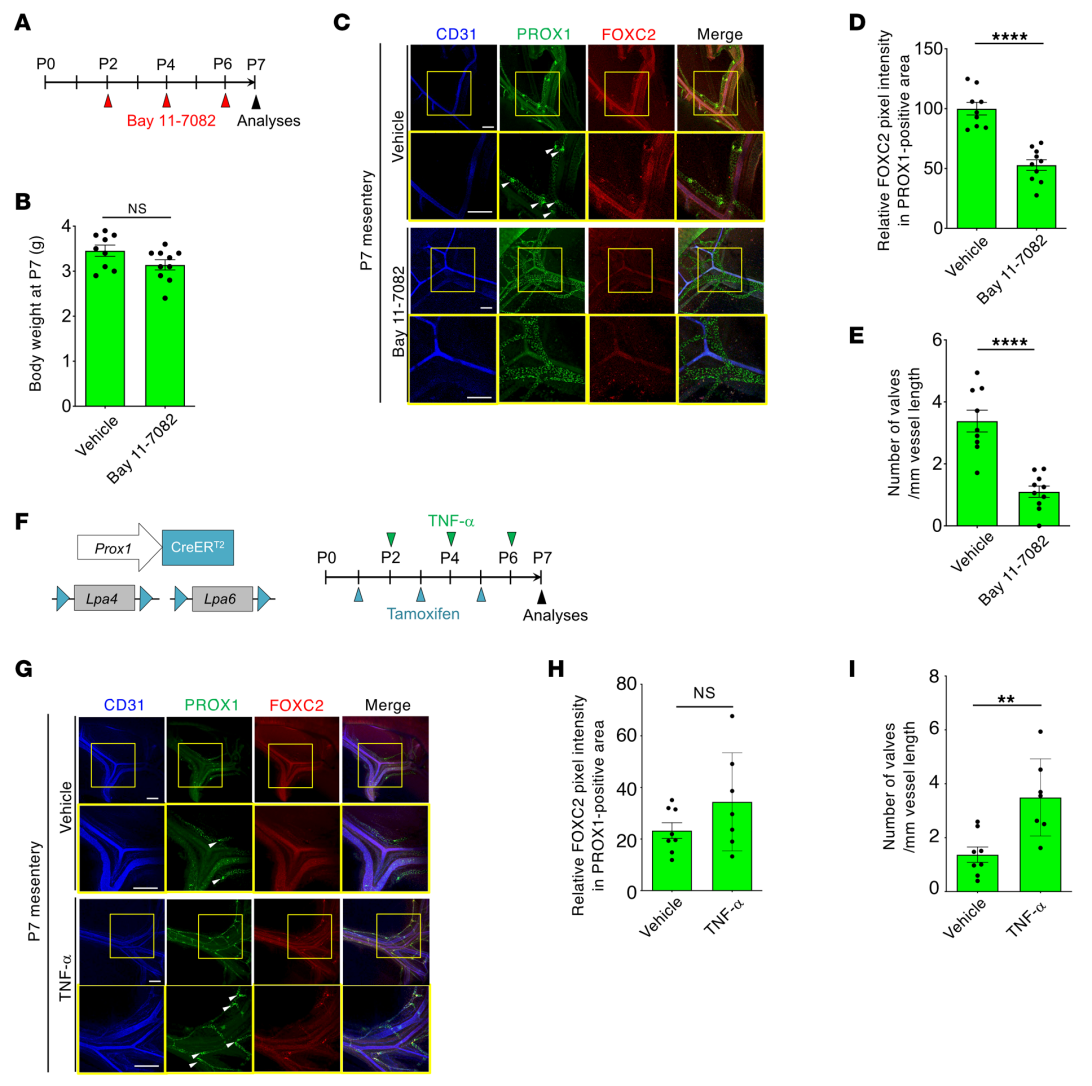
NF-κB activation increases FOXC2 expression and lymphatic valve number in neonatal mesenteric lymphatic vessels. (**A**) Schematic diagram of Bay 11-7082 (10 mg/kg) intraperitoneal administration to WT neonates analyzed at P7. (**B**) Body weights of vehicle and Bay 11-7082–treated mice at P7 (*n* = 9–10 mice). (**C**) Representative confocal images of mesenteric lymphatic vessels in WT mice treated with Bay 11-7082. Triple immunostaining for CD31, PROX1, and FOXC2 is shown. Areas in yellow boxes are magnified in the bottom panels. White arrowheads indicate lymphatic valves. Scale bars: 200 μm. (**D** and **E**) Quantification of FOXC2 expression in the PROX1^+^ area (**D**) and lymphatic valve numbers (**E**) (*n* = 9–10 mice). ****P < 0.0001; 2-tailed unpaired Student’s *t* test. (**F**) Schematic diagram of TNF-α (100 μg/kg) and tamoxifen administration to *Lpa4* *Lpa6^iΔLEC^* neonates analyzed at P7. (**G**) Representative confocal images of mesenteric lymphatic vessels in *Lpa4* *Lpa6^iΔLEC^* mice treated with TNF-α. Images of triple immunostaining for CD31, PROX1, and FOXC2 are shown. Areas in yellow boxes are magnified in the bottom panels. White arrowheads indicate lymphatic valves. Scale bars: 200 μm. (**H** and **I**) Quantification of FOXC2 expression in the PROX1^+^ area (**H**) and lymphatic valve numbers (**I**) (*n* = 7–8 mice). **P < 0.01; 2-tailed unpaired Student’s *t* test.

Following the in vitro experiments, NF-κB inhibitor Bay 11-7082 injection in neonatal mice (Figure 10A) did not affect body weight (Figure 10B), but significantly reduced FOXC2 expression (Figure 10, C and D) and the lymphatic valve number (Figure 10, C and E) in mesenteric lymphatic vessels. This effect was similar to that in *Lpa4* *Lpa6*^i*Δ*LEC^ pups (Figure 2, M–O, and Figure 5, C and D). Consistently, the valve formation defects of *Lpa4* *Lpa6*^i*Δ*LEC^ neonates could be rescued by TNF-α administration (Figure 10, F, G, and I). FOXC2 also showed a tendency to increase in a TNF-α administration–dependent manner, but no significant difference was observed (Figure 10, F–H). This may indicate that, owing to the transient TNF-α activity in vivo (49), the expression level of FOXC2 directly maintained by NF-κB began to decline more rapidly than the number of valves maintained by FOXC2. These results suggested that LPA4/LPA6-mediated NF-κB activation is required for lymphatic valve maintenance.

Previously, we reported that EC-specific *Lpa4*/*Lpa6* deletion impaired retinal angiogenesis in mice, which was caused by increased *Dll4* expression. Indeed, the Notch inhibitor DAPT exerted a significant ameliorating effect on the impaired angiogenesis (9). However, although there was an upward trend in *Dll4* mRNA levels in DKO LECs (Supplemental Figure 19A), DAPT had no rescue effect on lymphatic valve regression of *Lpa4* *Lpa6*^i*Δ*LEC^ neonates (Supplemental Figure 20). These findings suggested that Notch signaling is not responsible for lymphatic valve maintenance via LPA4/LPA6.

## Discussion

**Figure 11:**
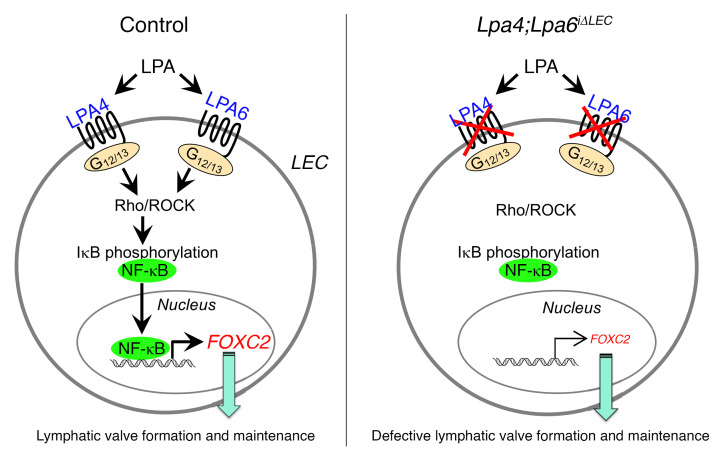
Schematic of the lymphatic endothelial LPA signaling pathway in lymphatic valve formation and maintenance. Lymphatic endothelial LPA4 and LPA6 activate Gα12/Gα13-ROCK signaling, which in turn induces NF-κB nuclear localization, subsequently increasing FOXC2 expression. LPA-induced FOXC2 expression is required for lymphatic valve development.

In this study, we revealed the LEC-intrinsic role of LPA4/LPA6 in lymphatic valve development in mice and its underlying molecular mechanism (Figure 11). Mice lacking LEC-specific LPA4 and LPA6 showed severely impaired lymphatic valve formation and maintenance, whereas those lacking either *Lpa4* or *Lpa6* alone showed normal lymphatic valves, suggesting that both LPA4 and LPA6 play essential and coordinated roles in lymphatic valve development. Mechanistically, we propose that LPA4/LPA6-Gα12/Gα13-ROCK signaling regulates FOXC2 expression through NF-κB activation in LECs. To further validate our proposal, future genetic evaluation is necessary to investigate whether LEC-specific deletions of LPA4/LPA6 signaling-related genes such as *Gna12/Gna13*, *Rock1/Rock2*, and *Rela* (encoding Gα12/Gα13, ROCK1/ROCK2, and p65, respectively) cause similar abnormalities in mouse lymphatic valves.

Our in vitro studies suggested that the role of LPA4/LPA6 in the regulation of FOXC2 is ligand dependent. Autotaxin-mediated digestion of lysophospholipids, such as LPC, is one of the mechanisms of LPA production (1). Autotaxin is secreted into the interstitial fluid by various cells, including fibroblasts and adipocytes (50, 51). As the lymph is derived from the interstitial fluid, it is reasonable that LPA is present in the mouse lymph along with lysophospholipids (3). The concentrations of LPA and LPC in mice were comparable between the lymph and blood plasma, indicating that LPA reaches sufficient concentrations to activate LECs in vivo (3). *Enpp2*, the gene encoding autotaxin, showed lower mRNA expression in DKO LECs than in control LECs (Supplemental Figure 19B), suggesting that LPA4/LPA6 signaling contributes to the regulation of *Enpp2* expression. Based on this observation, LPA may be produced in the lymph in a positive feedback loop with autotaxin secreted from LECs. The importance of autotaxin as a source of LPA required for lymphatic valve development may be revealed by analyzing *Enpp2*-deficient mice or mice treated with autotaxin inhibitors. In an autotaxin-independent mechanism, LPA is produced extracellularly through the deacylation of PA on the cell membrane, which is catalyzed by PA-PLA1α (2). The resulting LPA species have a fatty acid at the *sn*-2 position, making them the preferred ligand for LPA6 (7). In line with this, mutations in genes encoding LPA6 and PA-PLA1α both cause autosomal recessive woolly hair/hypotrichosis, an inherited hair disease (52, 53). Therefore, whether PA-PLA1α–derived LPA is important for the activation of lymphatic endothelial LPA4/LPA6 may be clarified by examining the lymphatic valves of these patients.

Selective coupling of ligand-activated GPCRs to specific Gα proteins is critical for intracellular signaling (54). Our in vitro experiments strongly supported the concept that Gα12/Gα13 activation mainly contributes to LPA4/LPA6-dependent LEC function. However, it is possible that other LPA receptors may be involved in lymphatic valve development. For example, LPA1, LPA2, and LPA5 can also couple to Gα12/Gα13 (5); however, their contributions to Gα12/Gα13 activation seemed rather limited in LECs under our experimental conditions. Accordingly, no lymphatic defects have been reported in LPA1-, LPA2-, or LPA5-deficient mice, probably because coupling selectivity between LPA receptors and Gα12/Gα13 proteins may vary depending on cell type, culture conditions, or receptor expression levels, as observed in S1P2 and S1P3, 2 Gα12/Gα13-coupled receptors for sphingosine-1-phosphates (S1Ps) (5). In murine cardiomyocytes, S1P3 but not S1P2 mediates Rho activation by S1P (55). Meanwhile, we do not rule out the possibility that other LPA receptors may participate in lymphatic development in concert with LPA4/LPA6; investigating the effects of other LEC-specific LPA receptor deficiencies on impaired lymphatic development in *Lpa4* *Lpa6*^i*Δ*LEC^ mice may reveal their cooperative contributions.

We demonstrated that LPA4/LPA6 activation induces ROCK-mediated NF-κB signaling in cultured LECs, which is concordant with previous studies showing that ROCK activates IKK and NF-κB, albeit in non-ECs (56, 57). Our results revealing that NF-κB signaling inhibitor treatment and LPA4/LPA6 deficiency caused consistent lymphatic valve regression in vivo strongly support the critical role of the GPCRs LPA4/LPA6 in maintaining lymphatic valves via NF-κB. To the best of our knowledge, this is the first study highlighting the significance of NF-κB signaling in valve homeostasis. Notably, severely immunocompromised patients with hypomorphic mutations in *IKBKG*, which encodes the γ subunit of IKK (also known as NEMO), have been reported to present with congenital lymphedema (58). Thus, this condition, called osteopetrosis, lymphedema, hypohidrotic ectodermal dysplasia, and immunodeficiency (OL-HED-ID) (59), may involve lymphatic valve defects. Our findings may improve understanding of lymphedema pathogenesis in patients with OL-HED-ID and inform the development of therapeutic strategies for lymphedema. In addition to lymphedema caused by congenital NF-κB blockade, drug-induced NF-κB blockade may also lead to lymphedema. Bortezomib, a proteasome inhibitor, is approved for the treatment of a variety of hematologic malignancies including multiple myeloma (60). This drug prevents NF-κB activation (61) and can potentially have the adverse effect, causing peripheral edema (62). In addition, dexamethasone inhibits NF-κB through the activation of the glucocorticoid receptor (63). Reportedly, dexamethasone caused edema as an adverse effect in patients with advanced cancer (64), despite having no mineralocorticoid activity that can cause edema if excessive sodium reabsorption and potassium excretion occur in the kidney (65). Collectively, lymphatic valve regression by NF-κB blockade may partially explain the mechanisms of edema induction caused by bortezomib and dexamethasone.

We showed here that LPA increases FOXC2 expression in LECs in an NF-κB–dependent manner. An NF-κB signaling inhibitor suppressed FOXC2 expression in murine prostate adenocarcinoma cells, thereby supporting our observations (66). In addition, the human FOXC2 promoter region to which NF-κB binds has been identified in non–small cell lung cancer cells (67). This finding indicates the possibility that NF-κB translocates to the nucleus through LPA4/LPA6 signaling and binds to the *FOXC2* promoter region to promote its transcription in LECs. To elucidate the underlying molecular mechanism, further investigation of NF-κB binding at the *Foxc2* promoter in mouse LECs that is affected by LPA stimulation or *Lpa4*/*Lpa6* ablation is necessary.

LEC-specific *Lpa4*/*Lpa6*-deficient mice phenocopied LEC-specific *Foxc2-*deficient mice, exhibiting impaired lymphatic valve formation (19) as well as dilation and excessive αSMA coverage of lymphatic vessels, while the number of lymphatic branches remained unaffected (19, 20). At the molecular level, PROX1, VEGFR3, and LYVE1 were overexpressed in global *Foxc2*-deficient lymphatics (18), which was also observed in DKO LECs. Collectively, we proposed a mechanism by which LPA induces FOXC2 expression via LPA4/LPA6 in LECs, which greatly contributes to valve formation. When gene ablation was induced postnatally, lymphatic valve regression was also observed in both LEC-specific *Lpa4*/*Lpa6*-deficient and *Foxc2*-deficient mice (19). Hence, LPA4/LPA6 signaling may also contribute to lymphatic valve maintenance via FOXC2. However, it remains unclear whether LPA4/LPA6 signaling modulates cell motility, as has been proposed for FOXC2 (19), and thereby contributes to the formation and maintenance of lymphatic valves in embryos and neonates, respectively.

Importantly, phenotypes of LEC-specific *Lpa4*/*Lpa6*-deficient mice were not completely identical to those of LEC-specific *Foxc2-*deficient mice. When induced postnatally, LEC-specific *Foxc2* ablation led to fully penetrant mortality in mice with chylous ascites and chylothorax (19). *Foxc2* ablation in LECs upregulates TAZ signaling that leads to unchecked proliferation followed by apoptosis, which possibly accounts for lymph leakage associated with the loss of intercellular junction integrity. In contrast, postnatal ablation of *Lpa4*/*Lpa6* in LECs did not result in chylous effusion or lethality. In vitro, DKO LECs did not affect apoptosis (Supplemental Figure 21) or proliferation (Supplemental Figure 22), suggesting that their intercellular junctions remained nearly intact. Previously, we reported that LPA4/LPA6 activate YAP/TAZ via ROCK in BECs (9). Therefore, in DKO LECs, excessive TAZ activation via downregulation of FOXC2 may be compensated for by blockade of the LPA4/LPA6-ROCK-TAZ signaling.

The lymphedema observed in LEC-specific *Lpa4*/*Lpa6*-deficient embryos is unlikely to be due to FOXC2 depletion. In contrast with patients with lymphedema distichiasis carrying *FOXC2* mutations (68) or global *Foxc2*-deficient mice (17), LEC-specific *Foxc2*-deficient embryos exhibit no symptoms of lymphedema (20, 37), suggesting a LEC-independent contribution of FOXC2 to lymphatic vessel development. In this study, LPA4/LPA6 depletion in LECs significantly reduced mRNA and protein expression of *Pdpn*, which encodes PDPN (Supplemental Figure 19, A and C–E). *Pdpn*-deficient mice developed fetal lymphedema (69), possibly because of venous blood reflux into the lymphatic system, thereby inhibiting lymph flow (70). Therefore, lymphedema in LEC-specific *Lpa4*/*Lpa6*-deficient embryos may be partially explained by decreased PDPN expression.

*Foxc2*-deficient embryos did not develop LVVs due to the absence of LVV-EC differentiation (71). Similarly, the LVVs were malformed in *Lpa4Lpa6*^i*Δ*LEC^ embryos at E15.5 that were administered tamoxifen at E10.5 and E11.5. We also observed LVV malformation in *Lpa4* *Lpa6*^i*Δ*LEC^ embryos at E17.5 that were administered tamoxifen at E12.5 and E13.5 despite the absence of edema or hemorrhage. While LVV malformation could potentially cause edema or hemorrhage in *Lpa4* *Lpa6*^i*Δ*LEC^ embryos at E15.5, this was not the case in E17.5 embryos. By E17.5, despite the presence of LVV defects, compensatory mechanisms may prevent edema or hemorrhage from occurring. For example, extensive platelet-mediated hemostasis around the lymphovenous junction may prevent retrograde blood flow into the lymphatic network, as observed in *Prox1*^+/–^ embryos at E17.5 lacking LVVs (72).

We previously demonstrated that LPA activates Rho/ROCK signaling via LPA4/LPA6 activation in BECs, similar to LECs (9). However, whether LPA4/LPA6 signaling activates NF-κB in BECs was unclear; therefore, we used HUVECs to observe the response of NF-κB signaling using a luciferase assay. The results showed that alkyl-OMPT stimulation significantly activated NF-κB, which was inhibited by pretreatment with Y27632 (Supplemental Figure 23A). Additionally, alkyl-OMPT stimulation significantly increased FOXC2 expression, which was also inhibited by pretreatment with Y27632 (Supplemental Figure 23B). These results suggest that NF-κB activation and FOXC2 expression induction by LPA4/LPA6 signaling are common phenomena in both BECs and LECs and that LPA4/LPA6 signaling in BECs may also be involved in venous valve formation.

OSS generated by lymph flow is critical for lymphatic valve development and FOXC2 expression (16, 73). Several molecules involved in sensing and translating OSS into FOXC2 expression have been identified, including VEGFR2/VEGFR3 (74), VE-cadherin (74, 75), and PIEZO1 (76, 77), along with their downstream effectors GATA2 (78), PROX1 (79), FOXO1 (77, 80), β-catenin (75, 81), and PI3K/AKT (75, 77). In contrast with OSS-induced signaling, LPA4/LPA6 signaling originates from GPCRs that respond to lymph-borne LPA (3) and increases FOXC2 expression via ROCK and NF-κB. When LPA4/LPA6 expression was depleted from LECs in newborn mice, the phenomenon of lymphatic valve regression accompanied by reduced *Foxc2* expression suggests that not only OSS signaling but also LPA4/LPA6 signaling is essential for valve maintenance. Therefore, the LPA4/LPA6-ROCK-NF-κB signaling axis appears to represent what we believe to be an unrecognized regulatory mechanism for FOXC2 expression and lymphatic valve development under OSS conditions.

In conclusion, we demonstrated that lymphatic endothelial LPA4/LPA6 play essential roles in lymphatic valve formation and maintenance during the embryonic and neonatal periods, respectively. NF-κB activation by the LPA4/LPA6-Gα12/Gα13-ROCK signaling axis is possibly indispensable for valve development. As an NF-κB target gene, *FOXC2*, which has been identified as an OSS-regulated gene (13), plays a dominant role. Our findings provide a molecular basis for lymphatic vessel development with a therapeutic potential for targeting tumor lymphatic metastasis, lymphatic malformation, and lymphedema.

## Methods

All procedures are described in detail in Supplemental Methods.

### Sex as a biological variable.

Both male and female mice were examined in this study, and similar findings were reported for both sexes.

### Statistics.

All data are presented as mean ± SEM and were analyzed using GraphPad Prism 8 software (GraphPad Software). Statistical significance between 2 groups was determined using a 2-tailed unpaired Student’s *t* test, Welch’s *t* test (for parametric analysis), or Mann-Whitney *U* test (for nonparametric analysis). One-way ANOVA followed by Tukey’s or Dunnett’s multiple-comparison test was used to compare 3 or more groups. Two-way ANOVA followed by Bonferroni’s multiple-comparison test was used for the proliferation assay. Differences were considered statistically significant at *P* < 0.05. No sample outliers were excluded from the analysis. Unless otherwise stated, individual in vitro experiments were performed at least twice with consistent results. In vitro experiments with mouse LECs were repeated using different batches of cells prepared on separate days. For in vitro experiments with LECs from control and *Lpa4 Lpa6^ΔEC^* mice, LECs from the same batch were compared. For in vivo experiments, data were collected from multiple independent experiments performed on different days. For histological analysis, *n* indicates the number of mice analyzed per genotype or treatment.

### Study approval.

All animal experimental procedures used in this study were approved by the Institutional Animal Care and Use Committee of Akita University (a-1-0465 and b-1-0446).

### Data availability.

The values underlying the data presented in each graph are included in the Supporting Data Values file.

## Author contributions

DY, KY, THY, T Shiiya, SM, and SI designed the experiments and wrote the manuscript. DY, NS, THY, T Shiiya, AT, and MH performed experiments and analyzed the data. KY, HS, and T Shimizu supported gene expression analyses and contributed to scientific discussion. SM and ST established *Prox1^+/Cre^* and *Prox1^+/CreERT2^* mouse lines. TE provided the *R26^+/tdTomato^* mouse line and contributed to scientific discussion. All authors reviewed and approved the manuscript.

## Funding support

- Japan Society for the Promotion of Science KAKENHI 19K07472 and 22K06877 (to DY), 23K18103 (to KY), and 19H03411 (to SI).
- The Japan Agency for Medical Research and Development (AMED)-PRIME 23gm6710020 (to KY).
- The Japan AMED Program for Basic and Clinical Research on Hepatitis 24fk0210150 (to HS).
- The National Center for Global Health and Medicine, Intramural Research Fund 22A1012 (to KY) and 22T001, 21A2006, and 24A2011 (to HS).
- The Takeda Science Foundation (to DY).
- The Ichiro Kanehara Foundation for the Promotion of Medical Sciences and Medical Care (to DY).
- The Mochida Memorial Foundation for Medical and Pharmaceutical Research (to DY).
- The Suzuken Memorial Foundation (to DY).
- The SENSHIN Medical Research Foundation (to DY).
- The Association for Research on Lactic Acid Bacteria (to SI).
- ONO Medical Research Foundation (to DY and HS).

## Supplementary Material

Supplemental data

Unedited blot and gel images

Supporting data values

## Version 1 01/13/2026

In-Press Preview

## Version 2 03/02/2026

Electronic publication
