# Supplementary material for: Lysophosphatidic acid–mediated NF-κB activation promotes FOXC2 expression essential for lymphatic valve development
Source: J Clin Invest. 2026 Jan 13;136(5):e193364. doi: 10.1172/JCI193364 (PMC12948439; doi:10.1172/JCI193364)
Supplement: Supplemental data [file jci-136-193364-s308.pdf]

Supplemental material

Lysophosphatidic acid-mediated NF-κB activation promotes FOXC2 expression essential for lymphatic valve development

Daisuke Yasuda, Nana Sato, Keisuke Yanagida, Tomomi Hashidate-Yoshida, Tomohiro Shiiya, Hideo Shindou, Atsuki Taira, Takashi Ebihara, Takao Shimizu, Masanori Hirashima, Seiya Mizuno, Satoru Takahashi, and Satoshi Ishii

It includes;

1. Supplemental Methods and their references
2. Supplemental Figures 1-29 and their legends
3. Supplemental Tables 1-7

SUPPLEMENTAL METHODS

Reagents.

LPA (1-oleoyl-LPA) was purchased from Avanti Polar Lipids (Alabaster, AL, USA) and stored at -80°C (10 mM stock in 50% ethanol). S1P (1 mM stock in methanol, stored at -80°C) and verteporfin (1 mM stock in dimethyl sulfoxide [DMSO], stored at -30°C) were bought from Cayman Chemical (Ann Arbor, MI, USA). ODP (10 mM stock in 50% ethanol, stored at -80°C) and alkyl-OMPT (10 mM stock in DMSO, stored at -30°C) were acquired from Echelon Biosciences (Salt Lake City, UT, USA). TC LPA5 4 (10 mM stock in DMSO, stored at -30°C) was purchased from R&D Systems (Minneapolis, MN, USA). Recombinant human TNF-α from R&D Systems (20 μg/mL) was prepared in phosphate-buffered saline (PBS) containing 1% bovine serum albumin (BSA; Sigma-Aldrich, St. Louis, MO, USA). SC75741 (10 mM stock in DMSO, stored at -30°C) and cycloheximide (10 mg/mL stock in DMSO, stored at -30°C) were obtained from Sigma-Aldrich. Adenosine 5'-triphosphate (ATP; 10 mM stock in water, stored at -30°C) was obtained from Wako (Osaka, Japan). Ki16425, H2L5186303, Y27632, and Bay 11-7082 were bought from Wako (all prepared as 10 mM stock in DMSO, stored at -30°C). CAPE (30 mM stock in DMSO, stored at -30°C) and DAPT (100 mg/mL stock in EtOH, stored at -30°C) were purchased from Tokyo Chemical Industry (Tokyo, Japan). Fasudil hydrochloride (100 mg/mL stock in water, stored at -30°C) was acquired from Selleck Chemicals (Houston, TX, USA).

Mice.

Lpa4- and *Lpa6*-floxed mice were generated as previously described (1, 2). *Prox1*$^{+/Cre}$ (Supplemental Table 1) and *Prox1*$^{+/CreERT2}$ (Supplemental Table 2) mouse lines were created using a modified CRISPR/Cas9 gene editing system (3). We selected a sequence (5′-GTT GCA ATC TCT ACT CGT GA-3′) containing the termination codon of *Prox1* as the single-guide RNA target. This sequence was inserted into the *pX330-mC* plasmid, which carries both guide RNA and Cas9-mC expression units (4). The donor DNA vectors *pCre-Prox1* and *pCreER*^{T2}-Prox1 included a P2A-Cre-rabbit globin polyadenylation sequence between the 5′ and 3′ homology arms. The 5′ arm contained the sequence from just before the termination codon to 1409 bp upstream, while the 3′ arm contained the sequence from just after the termination codon to 1340 bp downstream. The DNA vectors were isolated using a FastGene Plasmid Mini Kit (Nippon Genetics, Tokyo, Japan) and filtered with a MILLEX-GV 0.22-μm filter unit (Merck Millipore, Darmstadt, Germany) for microinjection. Pregnant mare serum gonadotropin (5 units; ASKA Animal Health, Tokyo, Japan) and human chorionic gonadotropin (5 units; ASKA Animal Health) were intraperitoneally injected into female C57BL/6 mice with a 48-hour interval. After mating with

male C57BL/6 mice, zygotes were collected from the oviducts of mated females and microinjected with a mixture of *pX330-mC* and either *pCre-Prox1* or *pCreER^{T2}-Prox1*. Subsequently, the surviving zygotes were transferred into the oviducts of pseudopregnant ICR females to obtain newborns. The B6.Cg-*Gt(ROSA)26Sor*$^{tm14(CAG\text{-}tdTomato)Hze}$/J mouse line, purchased from Jackson Laboratory (Farmington, CT, USA), was referred to as *R26*$^{+/tdTomato}$ reporter mouse line in this study. All mice shared a C57BL/6 genetic background.

Mouse breeding and genotyping.

Mice were housed under specific pathogen-free conditions in an air-conditioned room and fed standard laboratory chow (CE-2; CLEA Japan, Tokyo, Japan) *ad libitum* according to institutional guidelines. During mating (female mice >8 weeks old), vaginal plugs were checked every morning as an indication of copulation. E0.5 was defined as noon on the day the vaginal plug was detected. Pups were weaned at 3–4 weeks of age. Mouse genotypes were determined via PCR using genomic DNA extracted from tails and Quick Taq HS Dye Mix (Toyobo, Osaka, Japan). Primer sequences are listed in Supplemental Table 3.

Histological analysis.

To induce CreER^{T2}-mediated recombination in embryos, 2 mg tamoxifen (Sigma-Aldrich) dissolved in 20 mg/mL corn oil was intraperitoneally administered to pregnant mice on the indicated embryonic days. In neonates, CreER^{T2}-mediated recombination was induced by administering 50 μg of tamoxifen dissolved in corn oil at 20 mg/mL via oral gavage at P1, P3, and P5. Embryonic dorsal skins and neonatal mesenteries were collected from both male and female mice after euthanasia and fixed with 4% paraformaldehyde in PBS for 2 hours at 4°C. After three washes with PBS, specimens were permeabilized with PBS containing 0.3% Triton X-100 at room temperature for 10 minutes and blocked with PBS containing 1% BSA and 0.3% Triton X-100 at room temperature for another 2 hours. Samples were then incubated overnight at 4°C with primary antibodies (Supplemental Table 4) diluted in the blocking buffer. After three PBS washes, specimens were incubated for 2 hours at room temperature with the indicated fluorochrome-conjugated secondary antibodies (Supplemental Table 5) diluted in the blocking buffer. After four more PBS washes, flat-mounted specimens were imaged using a laser scanning confocal microscope (LSM 780 META, Carl Zeiss, Jena, Germany). To determine the effects of inhibitors on lymphatic valve maintenance, newborn pups were orally or intraperitoneally administrated with 50 mg/kg fasudil hydrochloride (dissolved in PBS) or 10 mg/kg Bay 11-7082 (dissolved in 1% DMSO/PBS), respectively, at P2, P4, and P6. Control mice were administered vehicle only. To examine the rescue effects of NF-κB activation and Notch inhibition on lymphatic valve regression, the tamoxifen-administered newborn *Lpa4 Lpa6*^{iΔLEC} pups were used.

They were administered 100 μg/kg mouse TNF-α (dissolved in 0.1% BSA/PBS) or 50 mg/kg DAPT (dissolved in ethanol/corn oil, 1:9 vol/vol) intraperitoneally or subcutaneously, respectively, at P2, P4, and P6. Control *Lpa4 Lpa6*$^{i\Delta LEC}$ pups were administered vehicle only. The width, branch point number, and valve number of lymphatic vessels were analyzed using ImageJ software (NIH). Briefly, vessel width was quantified by measuring the lymphatic vessel diameter at four randomly selected lymphangion segments. The number of vessel branch points and Prox1^{hi} clusters in embryonic dermal lymphatic vessels were counted and normalized to lymphatic vessel length. The number of valves in mesenchymal lymphatic vessels was also counted and normalized to lymphatic vessel length. For the analysis of filopodia, high-resolution confocal images were used. The number of filopodia per single sprout was measured. FOXC2 fluorescence intensity was measured in the Prox1^{+} area. VEGFR3 fluorescence intensity was measured in a 1,750 μm × 1,750 μm field. Fluorescence intensity of *Lpa4 Lpa6*$^{\Delta EC}$ or *Lpa4 Lpa6*$^{i\Delta LEC}$ mice was normalized to the mean fluorescence intensity of control mice analyzed in parallel. The investigators were blinded to genotype and animal treatment during data analysis.

In situ *hybridization.*

In situ hybridization was performed using RNAscope according to the instruction. Briefly, 10 μm-thick frozen sections were washed with PBS (Nacalai Tesque, Kyoto, Japan), baked using a HybEZ II Oven (Advanced Cell Diagnostics, Newark, CA, USA) for 30 minutes at 60°C, fixed with 4% PFA (Nacalai Tesque), and dehydrated. Samples were treated with hydrogen peroxide (Advanced Cell Diagnostics) for 10 minutes at room temperature, and then immersed in 1× target retrieval solution (Advanced Cell Diagnostics) for 5 minutes at 98-100°C. After drying overnight, samples were treated with proteinase plus (Advanced Cell Diagnostics) for 30 minutes at 40°C and hybridized with negative (Advanced Cell Diagnostics; Negative Control Probe-DapB, #310043), *Lpar4* (Advanced Cell Diagnostics; #318341), or *Lpar6* (Advanced Cell Diagnostics; #318351) specific probe for 120 minutes at 40°C. Probes were amplified using RNAscope 2.5 HD Detection Kit – RED (Advanced Cell Diagnostics, #322360). Samples were stained with 50% hematoxylin solution (Muto Pure Chemicals, Tokyo, Japan). After taking images using a NanoZoomer S60v2 slide scanner (Hamamatsu Photonics, Shizuoka, Japan), sections were stained with an anti-VEGFR3 antibody (Supplemental Table 4) overnight at 4°C. After washing with PBS, immune complexes were detected by Histofine Simple Stain MAX PO (G) (Nichirei Biosciences, Tokyo, Japan) at room temperature for 30 minutes with 3,3′-diaminobenzidine (Tokyo Chemical Industry) as a peroxidase substrate.

Lymphovenous valve observation.

For whole-mount preparation, E15.5 and E17.5 embryos were dissected and fixed with 4% PFA/PBS for 1 hour at 4°C. Fixed embryos were embedded in 2% agarose gel/PBS, cut frontally, and further fixed with 4% PFA/PBS overnight at 4°C. The embryos were washed three times with PBS containing 0.2% Triton X-100 (PBT) for 30 minutes at 4°C, blocked overnight at 4°C in PBT containing 1% BSA, and stained with primary antibodies (Supplemental Table 4) in blocking solution for a week at 4°C, followed by two vigorous overnight washes in PBT. Samples were incubated with secondary antibodies (diluted 1:1000; Jackson ImmunoResearch, West Grove, PA, USA, Supplemental Table 5) in blocking solution for another week, followed by two overnight washes in PBT. Stained embryos were dehydrated in methanol and cleared in a mixture of benzyl alcohol and benzyl benzoate (1:2). Confocal microscopy was carried out on an FV1200 (Olympus, Tokyo, Japan).

Lymphatic drainage analysis.

To evaluate lymphatic drainage function, 20 μl of 1 mg/mL FITC-dextran (MW 3000-5000, Sigma-Aldrich) diluted in PBS was injected into the footpads of the hind limbs of 6-week-old mice anaesthetized by intraperitoneal injection of 0.75 mg/kg medetomidine hydrochloride (Wako), 4.0 mg/kg midazolam (Wako), and 5.0 mg/kg butorphanol tartrate (Wako). Thirty minutes after injection, the draining lumbar aortic lymph nodes were harvested from the anesthetized mice. Fluorescent and bright-field images of the lymph nodes were acquired using a microscope (IX71; Olympus). FITC-dextran intensity in lymph node was measured using ImageJ software.

Cell culture and siRNA transfection.

HDLECs (#C-12216) were purchased from PromoCell (Heidelberg, Germany). HMVECs-L (#CC-2527, Lot: 18TL073809), HMVECs-dNeo (#CC-2505, Lot: 22TL152191), and HUVECs (#C2517A, Lot: 0000468310) were obtained from Lonza (Walkersville, MD, USA). These cells were cultured in collagen-coated 100-mm dishes in Endothelial Cell Growth Medium MV2 Kit (PromoCell) containing 20% fetal bovine serum (FBS) at 37°C in 5% CO_2. Cells between passages 4 and 7 were used for assays. Unless otherwise stated, cells were serum-starved for 8 hours in basal medium containing 0.1% BSA before ligand stimulation. For RNAi experiments, Silencer Select siRNAs targeting human *LPA4* (#s6043), *LPA6* (#s19797), *Gα12* (#s5865), *Gα13* (#s20991), and *RELA* (#s11914 and #s11915), as well as Negative Control No.1 were purchased from Thermo Fisher Scientific (Waltham, MA, USA); each used at 5 nM. siRNA transfection was performed using Lipofectamine RNAiMAX (Thermo Fisher Scientific), according to the manufacturer's instructions. The reduction in mRNA and protein expression by these siRNAs was confirmed (Supplemental Figure 24).

qRT-PCR analysis.

To prepare cDNA templates, total RNA was isolated using QIAzol lysis reagent (Qiagen, Valencia, CA, USA) and the Direct-zol RNA MicroPrep Kit (Zymo Research, Irvine, CA, USA). cDNA was synthesized from 500 ng of total RNA using the PrimeScript RT Reagent Kit (Takara Bio, Shiga, Japan) with random hexamers. Quantitative PCR was performed using a LightCycler 480 instrument (Roche Diagnostics, Mannheim, Germany) with either the KAPA SYBR Fast qPCR Kit (Kapa Biosystems, Wilmington, MA, USA) or GeneAce SYBR qPCR Mix II (Nippon Gene, Tokyo, Japan). The cycling conditions were as follows: initial denaturation at 95°C for 10 minutes, followed by 50 cycles of 95°C for 20 seconds and 60°C for 30 seconds. mRNA levels of target genes were normalized to those of the standard housekeeping gene *Gapdh*/*GAPDH* for mouse and human LECs, respectively. Primer sequences used to detect mouse and human mRNA expression are listed in Supplemental Tables 6 and 7, respectively.

Luciferase reporter assay.

LECs (2×10^4) and HUVECs (2×10^4) were seeded into collagen-coated 24-well plates and cultured for 24 hours. To detect Gα12/Gα13-ROCK activation, cells were transfected with a firefly luciferase reporter gene in the pGL4.34-SRF-RE vector (Promega, Madison, WI, USA) and *Renilla* luciferase reporter gene in the pRL-SV40 vector (Promega) using Lipofectamine 2000. After a 4-hour incubation at 37°C, cells were starved in 0.1% BSA for 1 hour, treated with 10 μM LPA or alkyl-OMPT for 6 hours, and lysed using Passive Lysis Buffer for the Dual Luciferase assay (Promega). To assess NF-κB activation, LECs were transfected with firefly luciferase reporter gene in the pGL4.32-NF-κB-RE vector (Promega) and *Renilla* luciferase reporter gene in the pRL-CMV vector (Promega) using PEI MAX (Polysciences, Warrington, PA, USA). After a 24-hour incubation at 37°C, cells were starved in 0.1% BSA for 4 hours, treated with 10 μM LPA or alkyl-OMPT for 6 hours, and lysed using Passive Lysis Buffer for the Dual Luciferase assay. In some experiments, cells were pre-treated with inhibitors for 1 hour before ligand stimulation. Firefly and *Renilla* luciferase activities in cell extracts were determined using a MiniLumat LB9506 luminometer (Berthold, Bad Wildbad, Germany). Firefly luciferase values were standardized to that of *Renilla*.

Intracellular calcium influx measurements.

LECs (3×10^6) were seeded into 10-cm collagen-coated dishes and cultured for 24 hours. Cells were detached with PBS containing 2 mM EDTA, washed with buffer A (Hanks' balanced salt solution containing 25 mM HEPES-NaOH [pH 7.4], 1 mM $CaCl_2$, 1 mM $MgCl_2$, and 0.1% BSA), and loaded with 3 μM Fura-2 AM (Dojindo, Kumamoto, Japan) in buffer A at 37°C for 1 hour.

Cells were washed with and resuspended in buffer A at a density of 1 × 10^6 cells/mL. A 0.5-mL cell suspension was analyzed using a CAF-110 spectrofluorometer (Jasco, Tokyo, Japan). Upon adding 5 μL of 100× ligand solution, the increase in intracellular Ca^{2+} concentration was detected by determining emission ratio at 500 nm after excitation at 340 and 380 nm (1). Adenosine 5'-triphosphate (ATP; 10 μM) was used as a positive control because ATP evokes calcium influx via P2Y receptors that predominantly couple with Gαq.

Measurement of cAMP production.

LECs (1 × 10^4) were seeded into collagen-coated 96-well plates and cultured for 24 hours. Cells were washed with buffer A and incubated in 50 μL of buffer A with 0.5 mM 3-isobutyl-1-methylxanthine (Sigma-Aldrich) for 15 minutes at room temperature. Reactions were initiated by adding 50 μL of 2× ligand solution with or without 40 μM forskolin (Sigma-Aldrich; from a 10 mM stock in DMSO stored at -30°C), to detect activation of Gαi or Gαs protein, respectively. After a 30-minute incubation at room temperature, reactions were terminated by adding 10 μL of 10% Tween 20, followed by overnight storage at 4°C. The cAMP concentration in cell lysate was determined using the AlphaScreen cAMP assay kit (PerkinElmer, Waltham, MA, USA) according to the manufacturer's instructions. Fluorescence was detected using an EnSpire multilabel plate reader (PerkinElmer).

Protein extraction and western blotting.

LECs were lysed in lysis buffer (50 mM Tris-HCl [pH 7.4], 150 mM NaCl, 1% Triton X-100, 1% sodium dodecyl sulfate [SDS], and 1% sodium deoxycholate) containing phosphatase inhibitors (1 mM Na_3VO_4 and 1 mM NaF) and a protease inhibitor cocktail (cOmplete; Roche Diagnostics). Lysates were centrifuged at 10,000 × *g* for 5 minutes, and the supernatants were collected. Protein concentrations were determined using a BCA assay (Thermo Fisher Scientific). The resulting protein samples were diluted in sample buffer (25 mM Tris-HCl [pH 6.5], 1% SDS, 5% glycerol, 0.05% bromophenol blue, and 5% 2-mercaptoethanol). Nuclear and cytosolic fractionations were performed using the LysoPure Nuclear and Cytoplasmic Extractor Kit (295-73901; Wako) according to the manufacturer's instructions. Briefly, LECs (1 × 10^6) were seeded into collagen-coated 60-mm dishes and cultured for 24 hours. Cells were washed with PBS and incubated with 250 μL of Nuclear Fractionation Buffer on ice for 10 minutes. Cells were harvested, vortexed, and centrifuged at 20000 × *g* for 10 minutes at 4°C. The supernatant was then transferred to a new tube as the cytosolic fraction. The pellet was suspended with 100 μL of SDS Lysis Buffer and sonicated using a Bioruptor 2 (Diagenode, Denville, NJ, USA) to prepare the nuclear fraction. Equal amounts of total proteins were electrophoresed on 8% SDS-polyacrylamide gels and transferred onto polyvinylidene difluoride membranes (Wako). After blocking with 5% skim milk

in TBS-T (20 mM Tris-buffered saline [pH 7.6] and 0.1% [v/v] Tween 20), the blots were incubated overnight at 4°C with one of the primary antibodies (Supplemental Table 4). The blots were then washed with TBS-T and incubated with horseradish peroxidase-conjugated secondary antibodies (Supplemental Table 5) for 1 hour at room temperature. Proteins were visualized using ImmunoStar LD (Wako) and a C-DiGit blot scanner (LI-COR Biotechnology, Lincoln, NE, USA).

Isolation of mouse lung LECs.

Lungs were excised from 3-week-old male and female mice, thoroughly minced with a razor blade, and digested with 50 U/mL DNase I (Sigma-Aldrich) and 0.26 U/mL Liberase (Roche Diagnostics) in Dulbecco's Modified Eagle Medium (DMEM) for 45 minutes at 37°C. Digested tissue was filtered through a 40-μm cell strainer (BD Biosciences, San Jose, CA, USA). Lung ECs were purified from the cell suspension by incubating at room temperature with anti-rat IgG Dynabeads (Thermo Fisher Scientific) preconjugated to a rat anti-mouse PECAM-1 antibody (#553370; BD Pharmingen, San Jose, CA, USA). Purified lung ECs were cultured on 2% gelatin-coated tissue culture dishes in DMEM supplemented with 20% FBS, 50 μg/mL Endothelial Cell Growth Supplement (Corning, Corning, NY, USA), 0.1 mg/mL heparin (Sigma-Aldrich), and 50 ng/mL recombinant human VEGF-C (Wako) until confluence. The cells were further purified after trypsinization using Dynabeads coupled with a rat anti-mouse Lyve1 antibody (#14-0443-82; Invitrogen). After cell expansion, the purity of LECs was evaluated. Briefly, cells were washed with PBS and detached using 0.25% trypsin/EDTA solution (Wako). Cells were incubated with 2 μg/mL rabbit anti-mouse LYVE-1 antibody (AngioBio, San Diego, CA, USA) and 1 μg/mL rat anti-mouse PDPN antibody (MBL International, Woburn, MA, USA) in PBS containing 1% BSA for 30 minutes at room temperature. After washing with PBS, the cells were incubated with PE-conjugated anti-rat and FITC-conjugated anti-rabbit IgG antibodies in PBS containing 1% BSA for 30 minutes at room temperature. Cells were then washed with PBS and analyzed using a BD Accuri C6 flow cytometer (BD Biosciences). In other experiments, cells were incubated with 1 μg/mL FITC-conjugated anti-mouse PDPN antibody (#127415; BioLegend, San Diego, CA, USA) and 1 μg/mL PE-conjugated anti-mouse CD31 antibody (#102507; BioLegend) in PBS containing 1% BSA for 30 minutes at room temperature. Cells were then washed with PBS and analyzed using a flow cytometer (BD Accuri C6). Mouse LEC passages were limited to two, taking care not to excessively reduce the density.

Sorting of mouse dermal LECs and BECs.

Dorsal skin was excised from neonatal mice at P1, carefully minced with a razor blade, and digested with 50 U/mL DNase I (Sigma-Aldrich), 1 mg/mL dispase II (Wako), and 0.26 U/mL Liberase (Roche Diagnostics) in DMEM for 45 minutes at 37°C. Digested tissue was filtered

through a 40-μm cell strainer (BD Biosciences). Cells were incubated with FITC-conjugated anti-mouse CD45 antibody (#103108; BioLegend), PE-conjugated anti-mouse CD31 antibody (#102507; BioLegend), PE-Cyanine7-conjugated anti-mouse LYVE-1 antibody (#25-0443-82; Thermo Fisher Scientific), and APC-conjugated anti-mouse PDPN antibody (#127415; BioLegend) for 30 minutes at room temperature. After washing with PBS, dermal LECs ($CD45^-CD31^+LYVE\text{-}1^+$ or $CD45^-CD31^+PDPN^+$) and BECs ($CD45^-CD31^+LYVE\text{-}1^-$ or $CD45^-CD31^+PDPN^-$) were sorted using a FACSMelody Cell Sorter (BD Biosciences).

Immunocytochemistry.

LECs were grown to confluence in collagen-coated glass-bottom dishes (Mat Tek Corporation, Ashland, MA, USA), washed with PBS, and fixed with 100% EtOH at room temperature for 5 minutes. Cells were permeabilized with 0.1% Tween 20/PBS at room temperature for 5 minutes, washed with PBS, and incubated with primary antibodies (Supplemental Table 4) overnight at 4°C. After washing with PBS, cells were then incubated with Alexa Fluor-conjugated secondary antibodies (diluted 1:200; Jackson ImmunoResearch; Supplemental Table 5) at room temperature for 2 hours. Nuclei were stained with 4′,6-diamidino-2-phenylindole (DAPI; diluted 1:100,000; BioLegend). Fluorescent images of labeled cells were acquired using a laser scanning confocal microscope (LSM 780 META; Carl Zeiss). Images of four randomly selected microscopic fields were taken, and cells were categorized based on the nuclear and cytoplasmic localization of the stained molecules; cells with much higher localization in the nucleus compared with the cytoplasm ($N >> C$), higher localization in the nucleus ($N > C$), equal localization in the nucleus and cytoplasm ($N = C$), or higher localization in the cytoplasm compared with the nucleus ($N < C$) were counted. The investigators were blinded to cell genotypes and treatments during counting.

Proliferation assay.

LECs were seeded into 96-well plates at 5.0×10^3 cells per well. Cell proliferation was analyzed using thiazolyl blue tetrazolium bromide (Sigma-Aldrich) according to the manufacturer's instructions. The absorbance of each well was measured at 570 nm using a 96-well microplate reader (Multiskan JX; Thermo Fisher Scientific).

Apoptosis assay.

LECs were seeded into 6-well plates at 2.0×10^5 cells per well and cultured for 24 hours. As a positive control, cells were treated with cycloheximide (10 μg/ml) and TNF-α (50 ng/ml) for 6 hours to induce apoptosis. Apoptosis was analyzed using the Annexin V-FITC Apoptosis Detection Kit (Nacalai Tesque) according to the manufacturer's instructions and a BD Accuri C6 flow cytometer (BD Biosciences).

References for supplemental methods

1. Yasuda D, Kobayashi D, Akahoshi N, Ohto-Nakanishi T, Yoshioka K, Takuwa Y, et al. Lysophosphatidic acid-induced YAP/TAZ activation promotes developmental angiogenesis by repressing Notch ligand Dll4. *J Clin Invest.* 2019;129(10):4332-4349.
2. Hata E, Sasaki N, Takeda A, Tohya K, Umemoto E, Akahoshi N, et al. Lysophosphatidic acid receptors LPA4 and LPA6 differentially promote lymphocyte transmigration across high endothelial venules in lymph nodes. *Int Immunol.* 2016;28(6):283-292.
3. Nakagawa Y, Oikawa F, Mizuno S, Ohno H, Yagishita Y, Satoh A, et al. Hyperlipidemia and hepatitis in liver-specific CREB3L3 knockout mice generated using a one-step CRISPR/Cas9 system. *Sci Rep.* 2016;6:27857.
4. Mizuno-Iijima S, Ayabe S, Kato K, Matoba S, Ikeda Y, Dinh TTH, et al. Efficient production of large deletion and gene fragment knock-in mice mediated by genome editing with Cas9-mouse Cdt1 in mouse zygotes. *Methods.* 2021;191:23-31.

SUPPLEMENTAL FIGURE 1

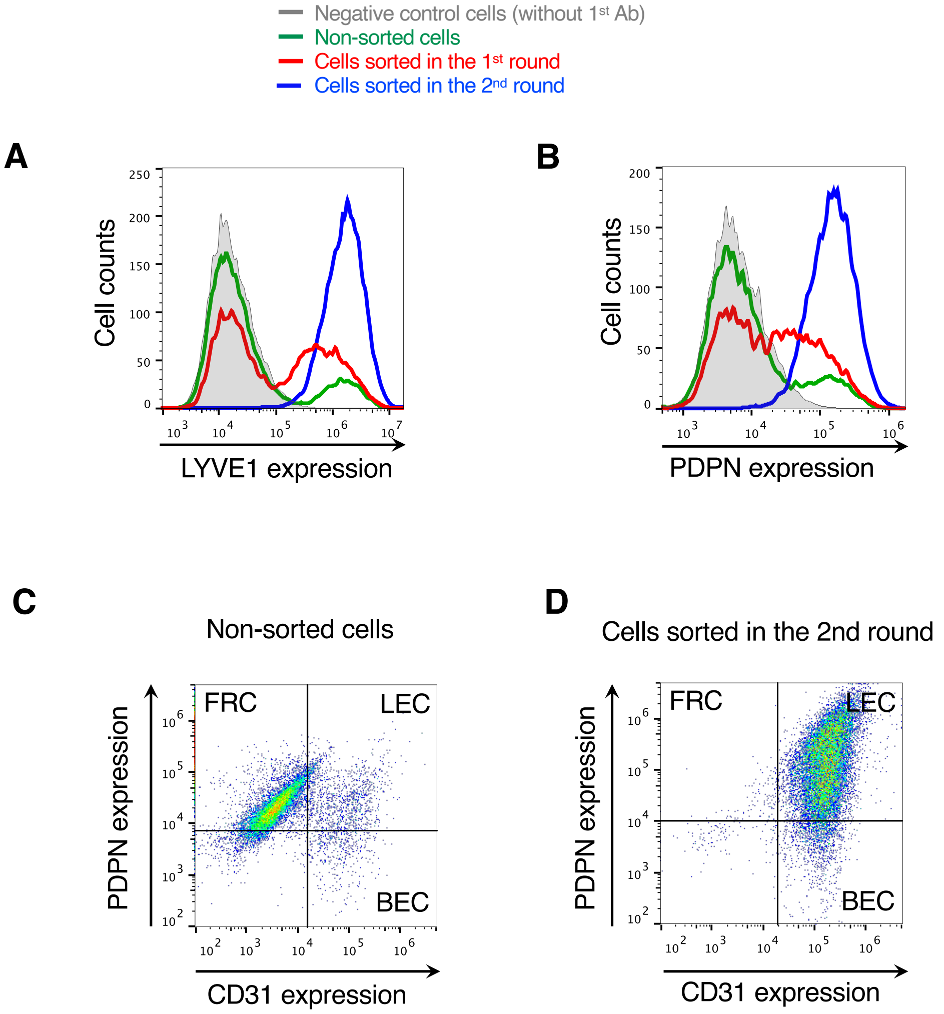

Supplemental Figure 1. LECs are isolated from mouse lungs using Dynabeads conjugated with anti-CD31 and anti-LYVE1 antibodies. (**A** and **B**) Overlay histograms of LYVE1 (**A**) and PDPN (**B**) expression in non-sorted cells (*green*), cells sorted using anti-CD31 antibody-conjugated Dynabeads in the 1st round (*red*), and cells sorted using anti-LYVE1 antibody-conjugated Dynabeads in the 2nd round (*blue*). For negative control cells (*gray*), histograms of non-sorted cells without 1st antibody for flow cytometry are shown. (**C** and **D**) Representative flow cytometry plots of PDPN and CD31 expression in non-sorted cells (**C**) and cells sorted in the 2nd round (**D**) (n = 3). Non-sorted cells contained a high proportion of fibroblastic reticular cells (FRCs; $PDPN^{+}CD31^{-}$), while cells sorted in the 2nd round contained only a small proportion of FRCs (2.9 ± 2.7%) and were primarily composed of LECs ($PDPN^{+}CD31^{+}$) (91.8 ± 1.1%). BECs ($PDPN^{-}CD31^{+}$) accounted for 4.7 ± 2.0 %.

SUPPLEMENTAL FIGURE 2

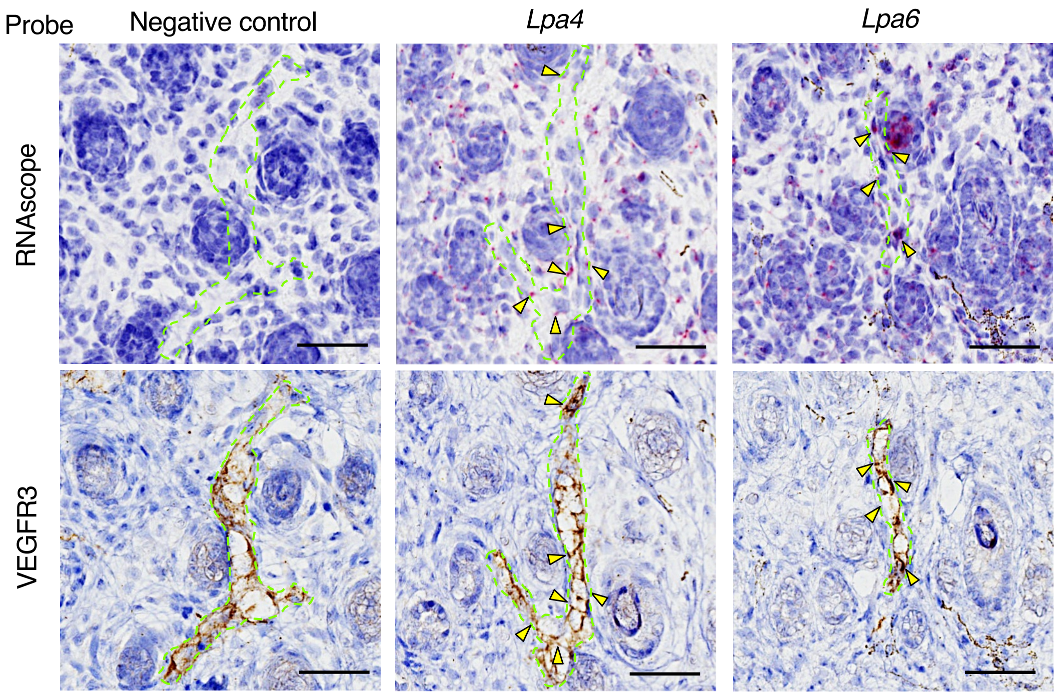

Supplemental Figure 2. RNAscope *in situ* hybridization shows the mRNA expression of *Lpa4* and *Lpa6* in lymphatic vessels of the dorsal skin at E18.5. Representative RNAscope images for indicated probes with hematoxylin counter stain (upper panels) and the same sections immunostained with VEGFR3 antibody to visualize the lymphatic vessels (lower panels, *green dashed line*). In RNAscope assays, red signals indicate the presence of specific mRNA molecules in lymphatic vessels (*yellow arrowheads*) by *Lpa4* (center upper panel) and *Lpa6* (right upper panel) probes but not negative control probe (left upper panel). Scale bars, 50 μm. All images are representatives of three embryos.

SUPPLEMENTAL FIGURE 3

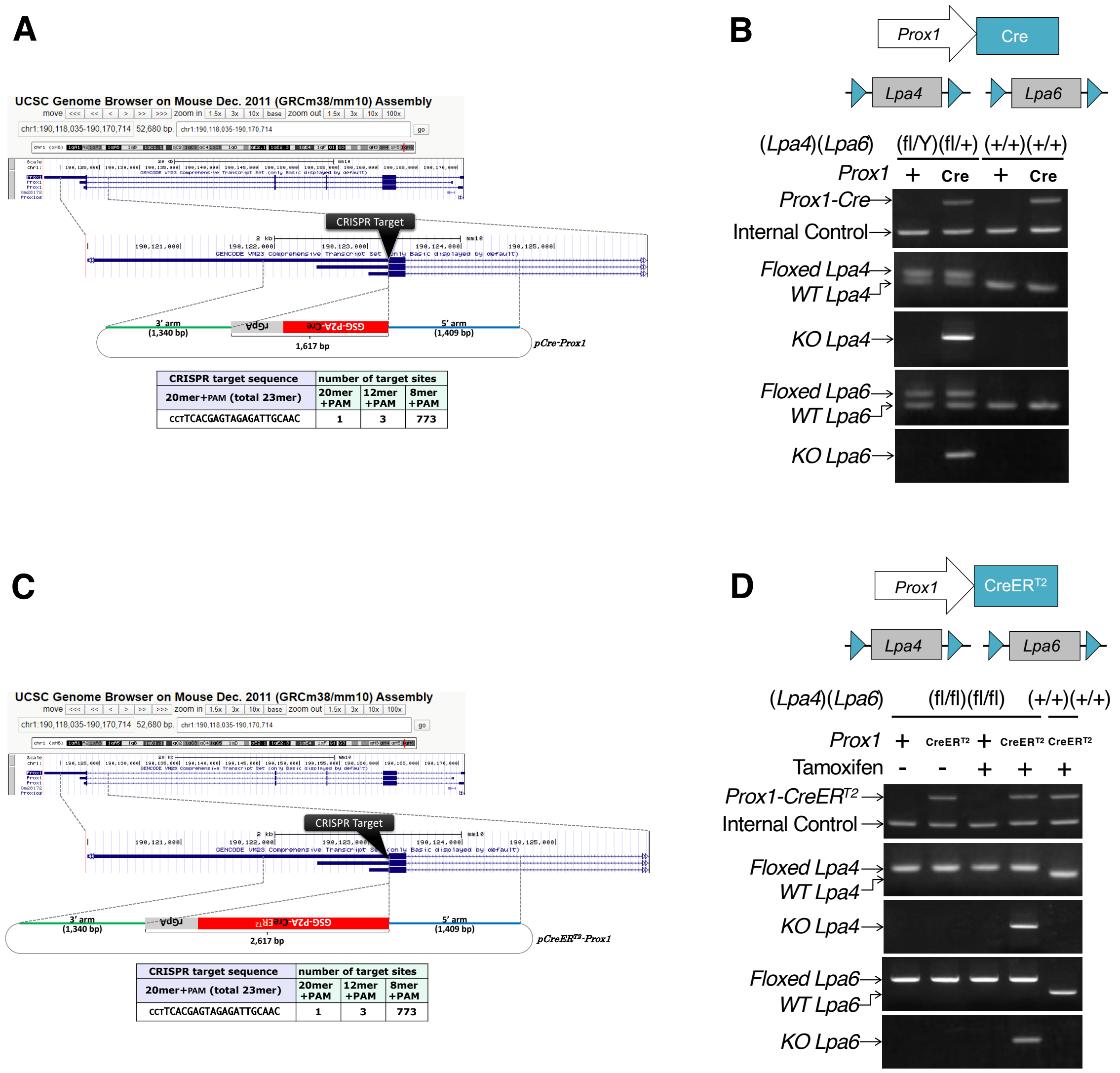

Supplemental Figure 3. *Prox1-Cre* and *Prox1-CreER^{T2}* alleles are established using CRISPR/Cas9-mediated homologous recombination. (**A**) Schematic diagram of *Prox1-Cre* allele construction. (**B**) PCR genotyping of neonatal tail DNA was performed to detect WT, floxed, and KO alleles of *Lpa4* and *Lpa6*, as well as *Prox1-Cre* allele. *Internal Control* sequence present in both *WT Prox1* and *Prox1-Cre* alleles was used as a positive control. Nucleotide sequences of *Prox1-Cre* allele are listed in Supplemental Table 1. (**C**) Schematic diagram of *Prox1-CreER^{T2}* allele construction. (**D**) Generation of *Lpa4;Lpa6$^{i\Delta LEC}$* mice. PCR genotyping of neonatal tail DNA was performed to detect WT, floxed, and KO alleles of *Lpa4* and *Lpa6*, as well as *Prox1-CreER^{T2}* allele. *Internal Control* sequence present in both *WT Prox1* and *Prox1-CreER^{T2}* alleles was used as a positive control. Nucleotide sequences of *Prox1-CreER^{T2}* allele are presented in Supplemental Table 2. PCR primers used to detect these alleles are listed in Supplemental Table 3. Unprocessed original PCR genotyping scans are shown in Supplemental Figure 25.

SUPPLEMENTAL FIGURE 4

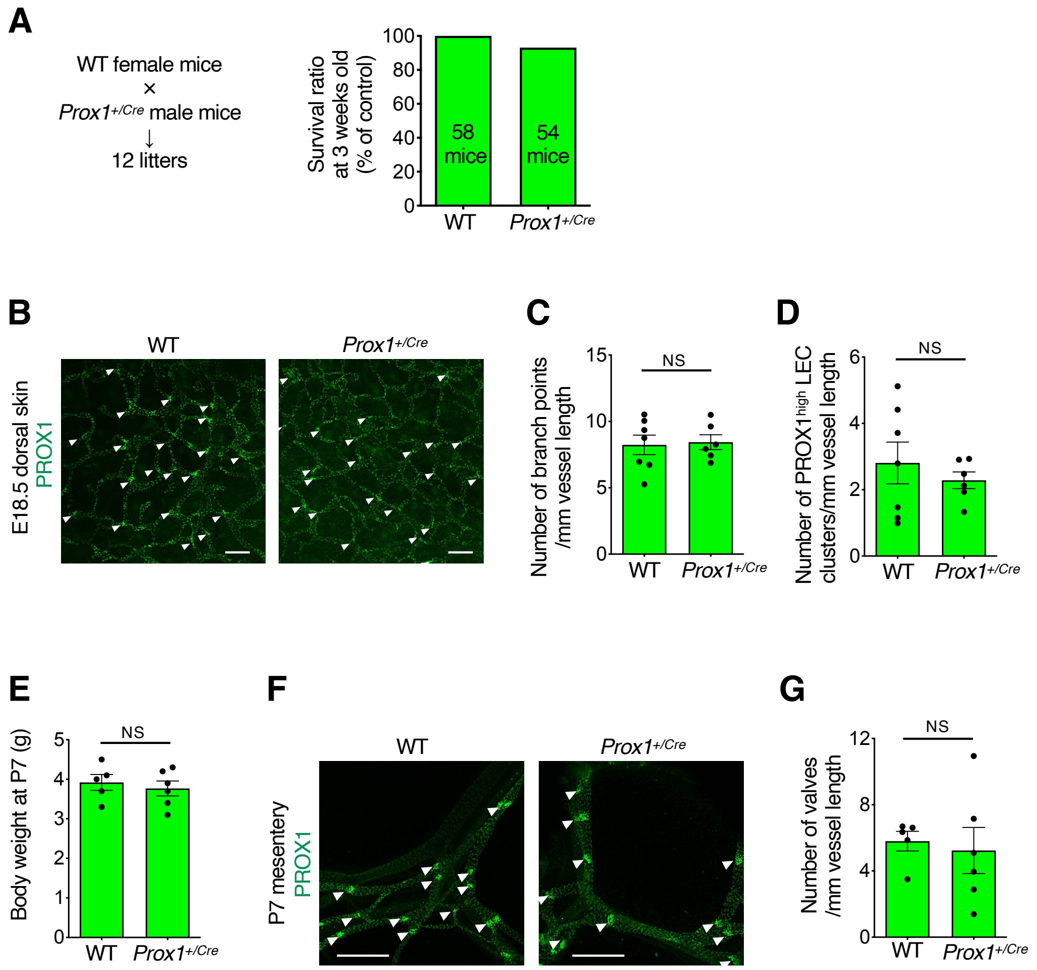

Supplemental Figure 4. *Prox1-Cre* allele does not affect fertility, body weight, or lymphatic valve formation. (**A**) Generation of *Prox1*$^{+/Cre}$ mice with the expected Mendelian ratio. Data obtained at 3 weeks of age are shown. The numbers of mice are indicated in the bars. (**B**) Representative confocal images of lymphatic vascular networks of the dorsal skin at E18.5. The lymphatic vessels were stained with an anti-PROX1 antibody. White arrowheads indicate putative valve-forming PROX1^{high} LEC clusters. Scale bars, 200 µm. (**C** and **D**) Quantification of vessel branch number (**C**) and the number of PROX1^{high} LEC clusters (**D**) in WT and *Prox1*$^{+/Cre}$ mice. (**E**) Body weights of WT and *Prox1*$^{+/Cre}$ mice at P7. (**F**) Representative confocal images of mesenteric lymphatic vessels in WT and *Prox1*$^{+/Cre}$ mice at P7, showing PROX1 immunostaining. White arrowheads indicate lymphatic valves. Scale bars, 200 µm. (**G**) Quantification of lymphatic valve number. Two-tailed unpaired Student's *t*-test. NS, not significant.

SUPPLEMENTAL FIGURE 5

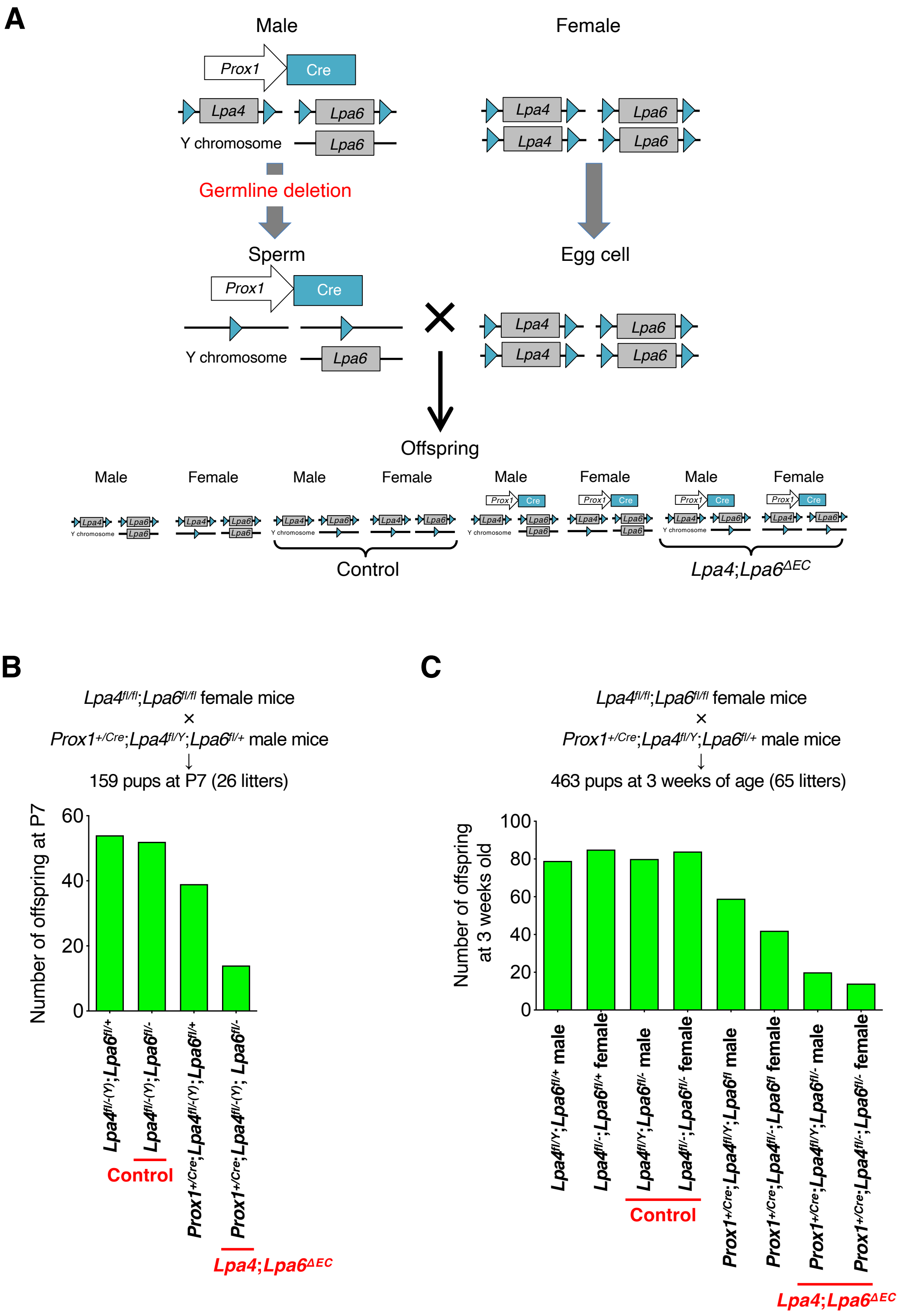

Supplemental Figure 5. *Lpa4*;*Lpa6*$^{\Delta EC}$ mice birth rate is approximately 75% lower than expected from the Mendelian ratio. (**A**) Schematic diagram of *Prox1* promoter-driven Cre-mediated germline deletion and genotypes of the resulting offspring. (**B**) Number of offspring of each genotype at P7. Sex was not determined during this stage. (**C**) Number of offspring of each genotype at 3 weeks of age.

SUPPLEMENTAL FIGURE 6

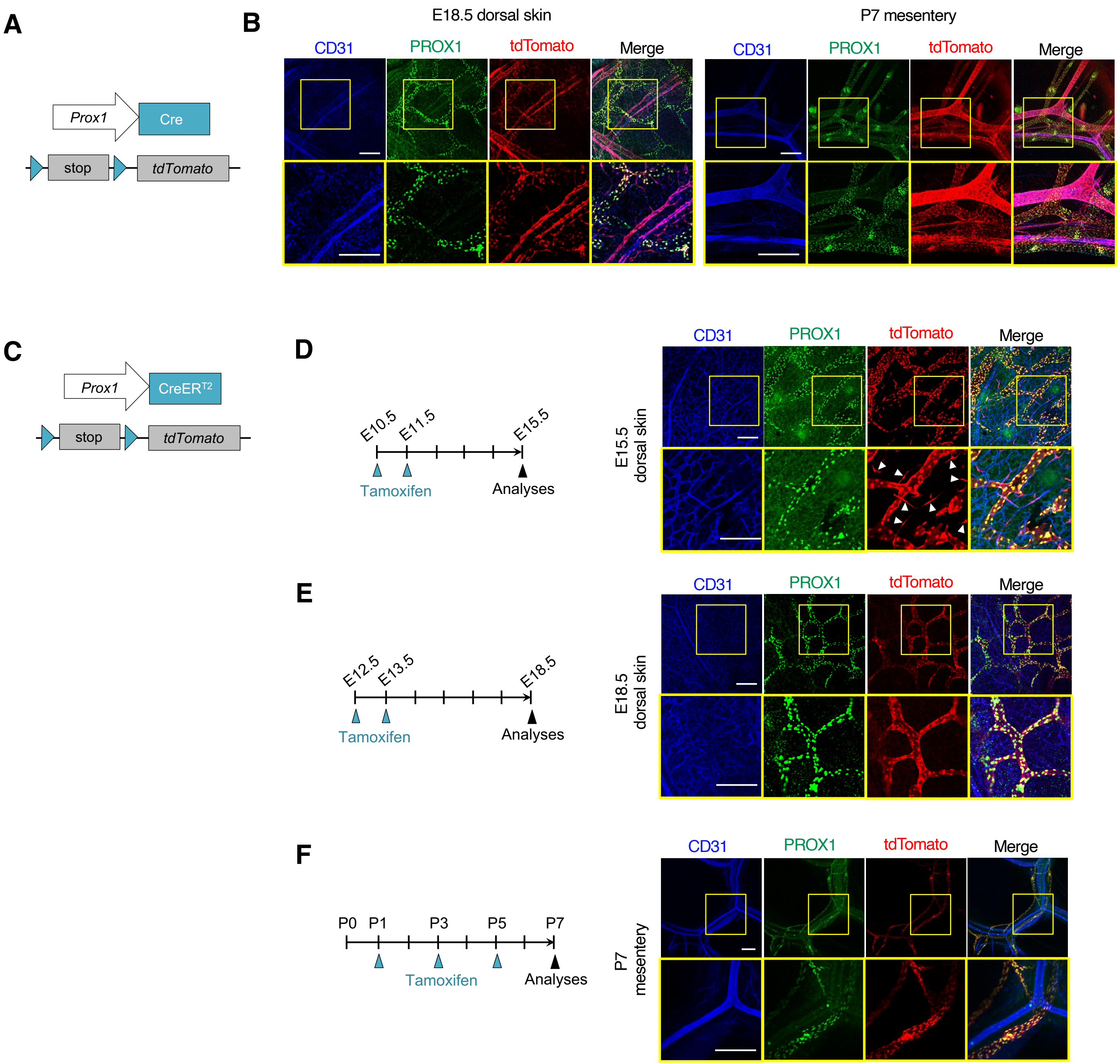

Supplemental Figure 6. Cre-mediated tdTomato expression in ECs and LECs is detected in *Prox1*$^{+/Cre}$ and *Prox1*$^{+/CreERT2}$ mice, respectively. (**A**) Schematic diagram of *R26-tdTomato* reporter activation by *Prox1-Cre* allele. (**B**) Representative confocal images of lymphatic vessels in the dorsal skin at E18.5 and mesenteric vessels at P7. Intrinsic tdTomato fluorescence and PROX1 immunostaining are shown (*n* = 4 and 7 mice at E18.5 and P7, respectively). Areas marked by yellow line boxes are magnified at the bottom. Scale bars, 200 μm. (**C**) Schematic diagram of tamoxifen-induced *R26-tdTomato* reporter activation by *Prox1-CreER*^{T2} allele. (**D**–**F**) Tamoxifen injection procedures for analysis at E15.5 (**D**), E18.5 (**E**) and P7 (**F**). Representative confocal images of lymphatic vessels in the dorsal skin at E15.5 (**D**) and E18.5 (**E**) and mesenteric vessels at P7 (**F**). Intrinsic tdTomato fluorescence and CD31 and PROX1 immunostaining are displayed (*n* = 3 mice). Areas marked by yellow line boxes are magnified at the bottom. White arrowheads indicate tamoxifen-induced tdTomato fluorescence in blood vessels (**D**). Scale bars, 200 μm.

SUPPLEMENTAL FIGURE 7

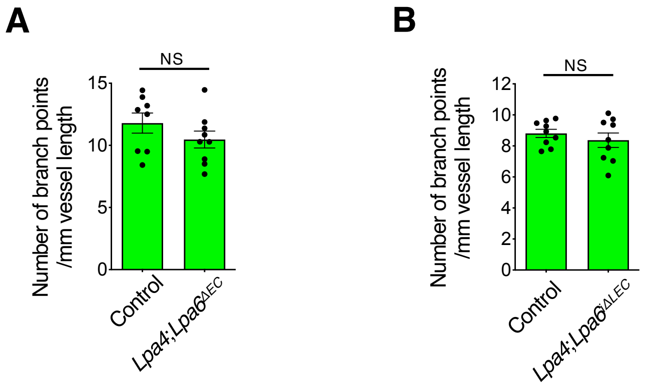

Supplemental Figure 7. Lymphatic endothelial *Lpa4*/*Lpa6* ablation does not affect lymphatic vessel branching in mice. (**A**) Quantification of branch numbers in lymphatic vascular networks of the dorsal skin at E16.5 in control and *Lpa4*;*Lpa6*$^{\Delta EC}$ embryos (related to Figure 1I). Data are presented as mean ± SEM (*n* = 8–9 embryos). Two-tailed unpaired Student's *t*-test. NS, not significant. (**B**) Quantification of branch numbers in lymphatic vascular networks of the dorsal skin at E17.5 in control and *Lpa4*;*Lpa6*$^{i\Delta LEC}$ embryos (related to Figure 2H). Data are presented as mean ± SEM (*n* = 9 embryos). Two-tailed unpaired Student's *t*-test. NS, not significant.

SUPPLEMENTAL FIGURE 8

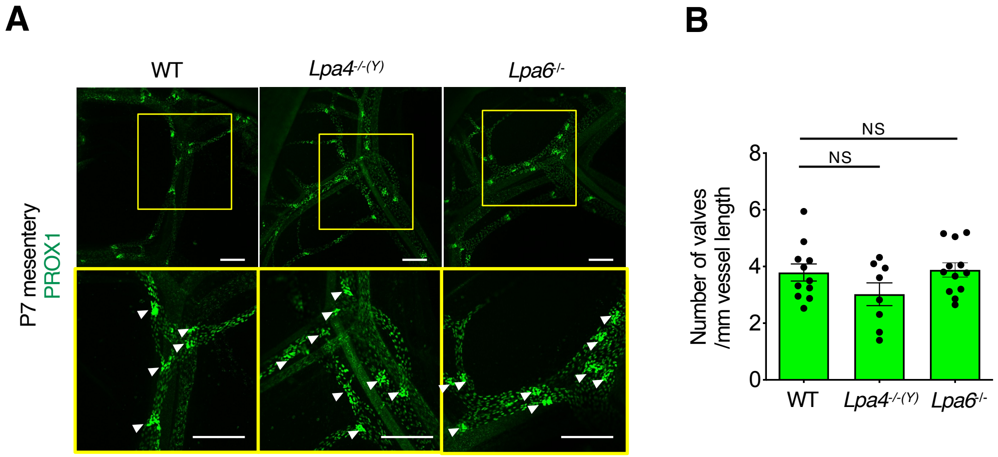

Supplemental Figure 8. Lymphatic valve formation in global *Lpa4*$^{-/-(Y)}$ and *Lpa6*$^{-/-}$ mice is normal. (**A**) Representative confocal images of mesenteric lymphatic vessels at P7, showcasing PROX1 immunostaining. Areas marked with yellow line boxes are magnified at the bottom. White arrowheads indicate lymphatic valves. Scale bars, 200 μm. (**B**) Quantification of lymphatic valve number. Data are presented as mean ± SEM (*n* = 8–12 mice). One-way ANOVA followed by Dunnett's test. NS, not significant.

SUPPLEMENTAL FIGURE 9

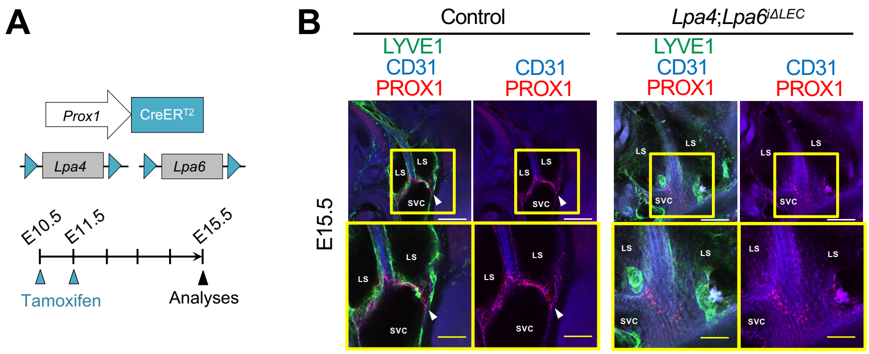

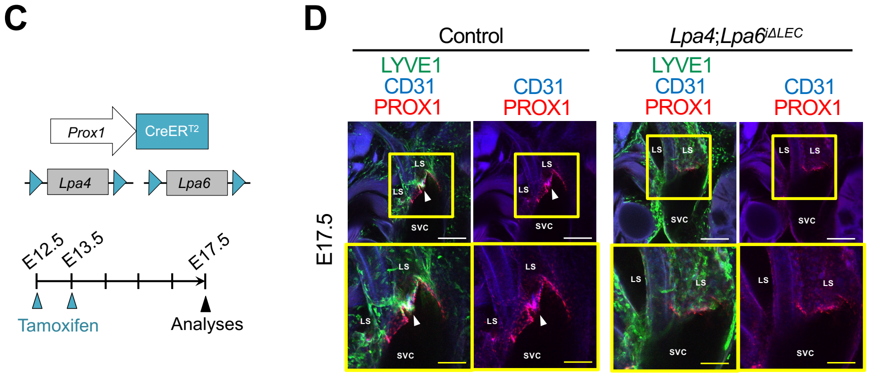

Supplemental Figure 9. Lymphatic endothelial LPA4/LPA6 are required for LVV development. (**A**) Schematic diagram of *Lpa4* and *Lpa6* ablation in *Lpa4*;*Lpa6*$^{i\Delta LEC}$ mice and tamoxifen injection procedure for analysis at E15.5. (**B**) Representative confocal images of LVV at E15.5. Images of triple (left panels) and double (right panels) immunostaining for CD31, PROX1, and/or LYVE1 are shown. White arrowheads indicate LVV. LS, lymph sac; SVC, superior vena cava. Areas marked by yellow line boxes are magnified at the bottom. White scale bars, 200 µm. Yellow scale bars, 100 µm. (*n* = 4–5 embryos). (**C**) Schematic diagram of *Lpa4* and *Lpa6* ablation in *Lpa4*;*Lpa6*$^{i\Delta LEC}$ mice and tamoxifen injection procedure for analysis at E17.5. (**D**) Representative confocal images of LVV at E17.5. Images of triple (left panels) and double (right panels) immunostaining for CD31, PROX1, and/or LYVE1 are shown. White arrowheads indicate LVV. LS, lymph sac; SVC, superior vena cava. Areas marked by yellow line boxes are magnified at the bottom. White scale bars, 200 µm. Yellow scale bars, 100 µm. (*n* = 8-9 embryos).

SUPPLEMENTAL FIGURE 10

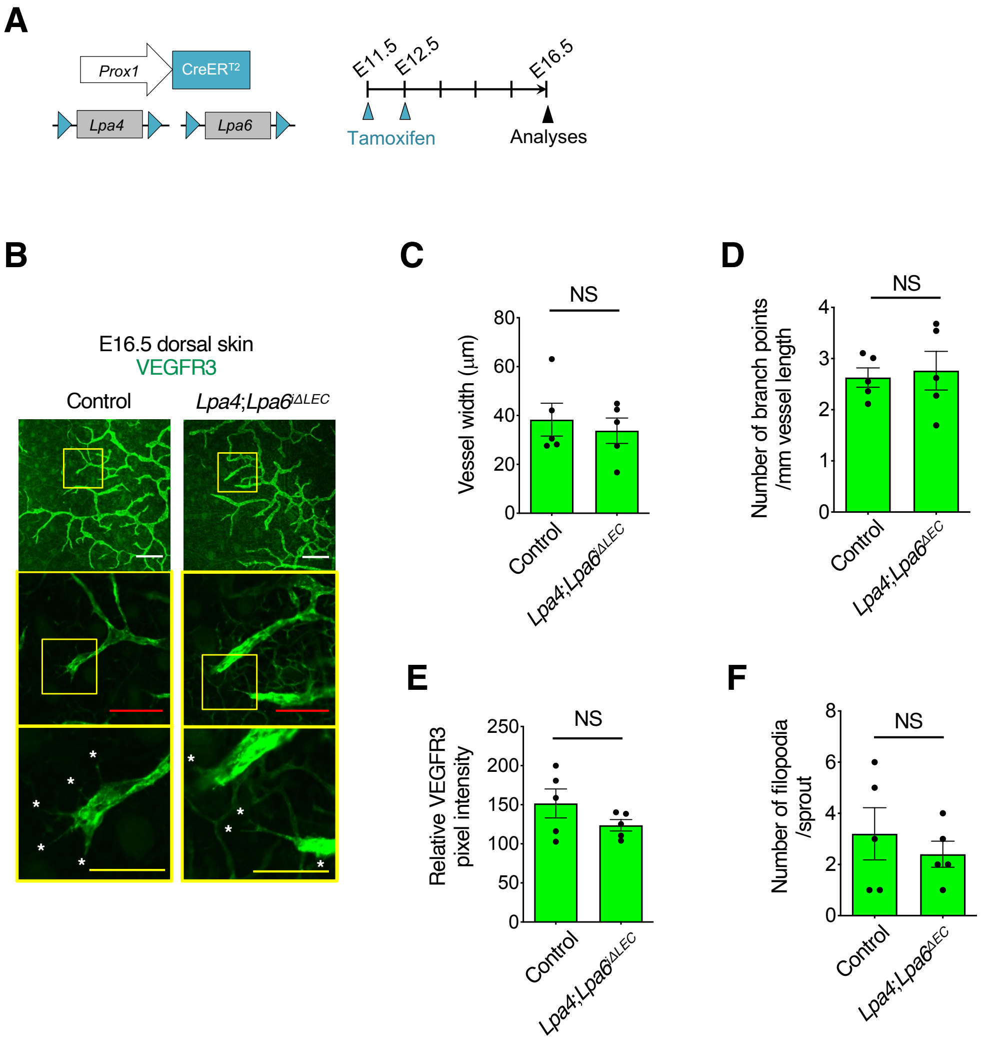

Supplemental Figure 10. Lymphatic endothelial LPA4/LPA6 are not essential for development of capillary lymphatic vessels. (**A**) Schematic diagram of *Lpa4* and *Lpa6* ablation in *Lpa4*;*Lpa6*$^{i\Delta LEC}$ mice and tamoxifen injection procedure for analysis at E16.5. (**B**) Representative confocal images of capillary lymphatic vessels in the dorsal skin, showcasing VEGFR3 immunostaining. Areas marked by yellow line boxes are magnified at the bottom. Asterisk indicates filopodia. White scale bars, 200 µm. Red scale bars, 100 µm. Yellow scale bars, 50 µm. (**C**–**F**) Quantification of vessel width (**C**), number of branch points (**D**), mean fluorescence intensity of VEGFR3 (**E**), and filopodia number (**F**) in control and *Lpa4*;*Lpa6*$^{i\Delta LEC}$ embryos (n = 5 embryos). Two-tailed unpaired Student's t-test. NS, not significant.

SUPPLEMENTAL FIGURE 11

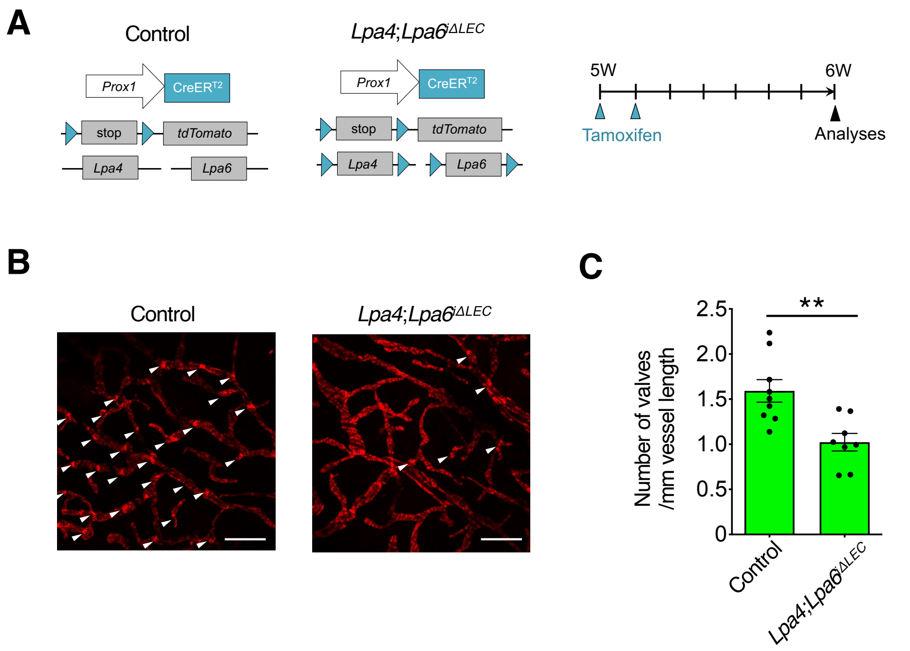

Supplemental Figure 11. Lymphatic endothelial LPA4/LPA6 are essential for lymphatic valve maintenance in the adult ear. (**A**) Schematic diagram of *R26-tdTomato* reporter activation, *Lpa4*/*Lpa6* ablation in *Lpa4*;*Lpa6*$^{i\Delta LEC}$ mice, and tamoxifen injection procedure for analysis in 6-week-old mice. (**B**) Representative confocal images of lymphatic vessels in the ear. Intrinsic tdTomato fluorescence is shown. White arrowheads indicate lymphatic valves. Scale bars, 200 µm. (**C**) Quantification of lymphatic valve number (n = 8–9 mice). **P < 0.01, two-tailed unpaired Student's t-test.

SUPPLEMENTAL FIGURE 12

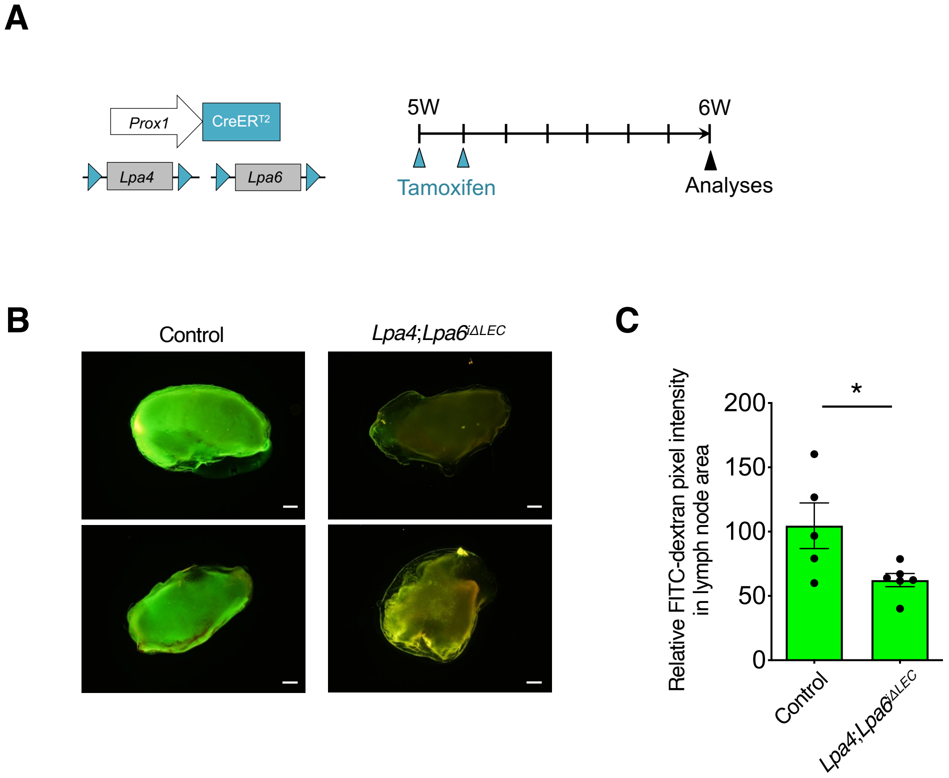

Supplemental Figure 12. *Lpa4*;*Lpa6*$^{i\Delta LEC}$ mice exhibit decreased lymphatic draining efficiency. (**A**) Schematic diagram of *Lpa4*/*Lpa6* ablation in *Lpa4*;*Lpa6*$^{i\Delta LEC}$ mice, and tamoxifen injection procedure for analysis in 6-week-old mice. (**B**) Representative FITC-dextran fluorescence images of the draining lumbar aortic lymph nodes. Scale bars, 200 µm. (**C**) Quantification of FITC-dextran fluorescence in lymph node area (n = 5–6 mice). *P < 0.05, two-tailed unpaired Student's t-test.

SUPPLEMENTAL FIGURE 13

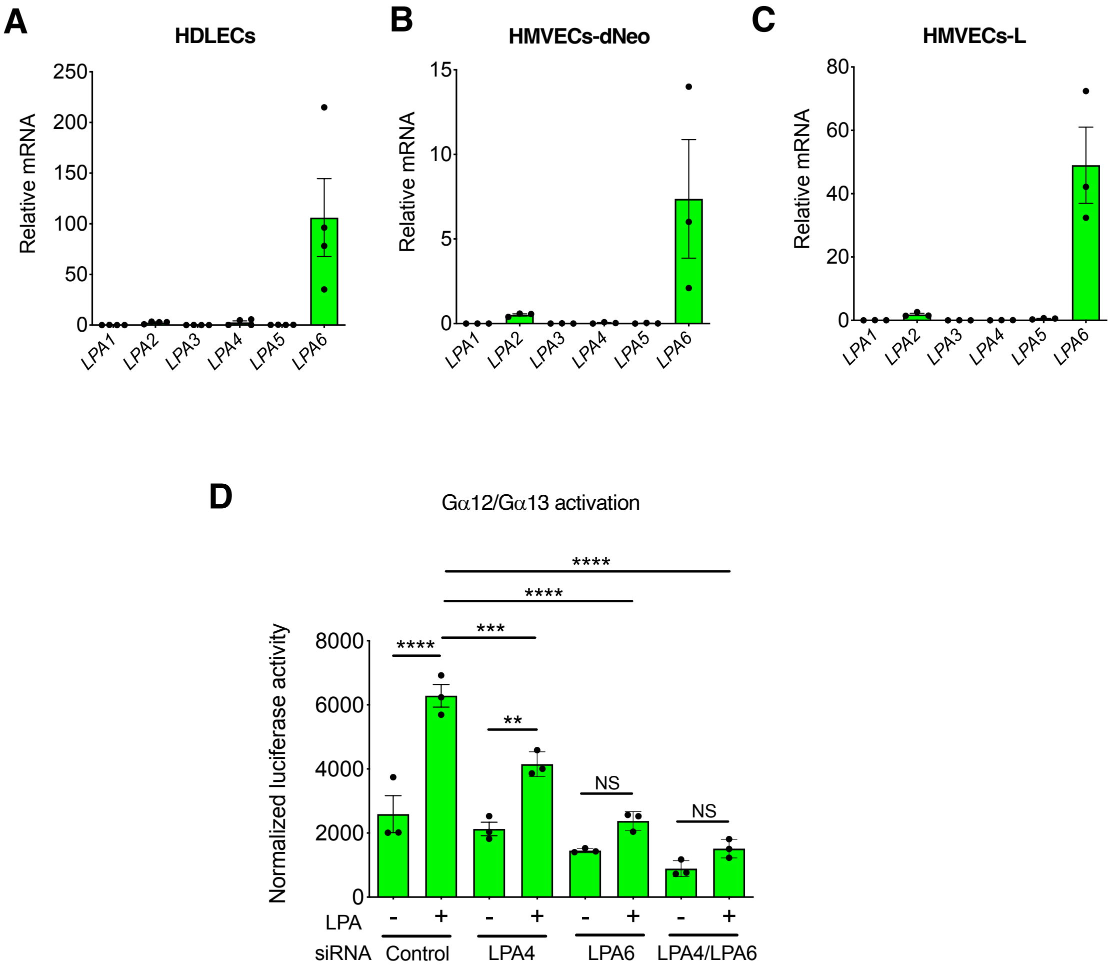

Supplemental Figure 13. LPA induces SRF-RE luciferase reporter activity in human LECs, which predominantly express *LPA6* mRNA. (**A**–**C**) Expression of LPA receptor mRNA in three human LECs, namely HDLECs (**A**), HMVECs-dNeo (**B**), and HMVECs-L (**C**), as detected by qRT-PCR. (**D**) SRF-RE-Luc reporter assay to detect Gα12/Gα13-Rho activation. LPA (10 μM, 6 hours) increased reporter activity, which was attenuated by *LPA6* siRNA treatment (48-hour pretreatment) in serum-starved HDLECs. Data are presented as mean ± SEM of triplicates. $^{**}P < 0.01$, $^{***}P < 0.001$, $^{****}P < 0.0001$, one-way ANOVA followed by Tukey's multiple comparisons test. NS, not significant.

SUPPLEMENTAL FIGURE 14

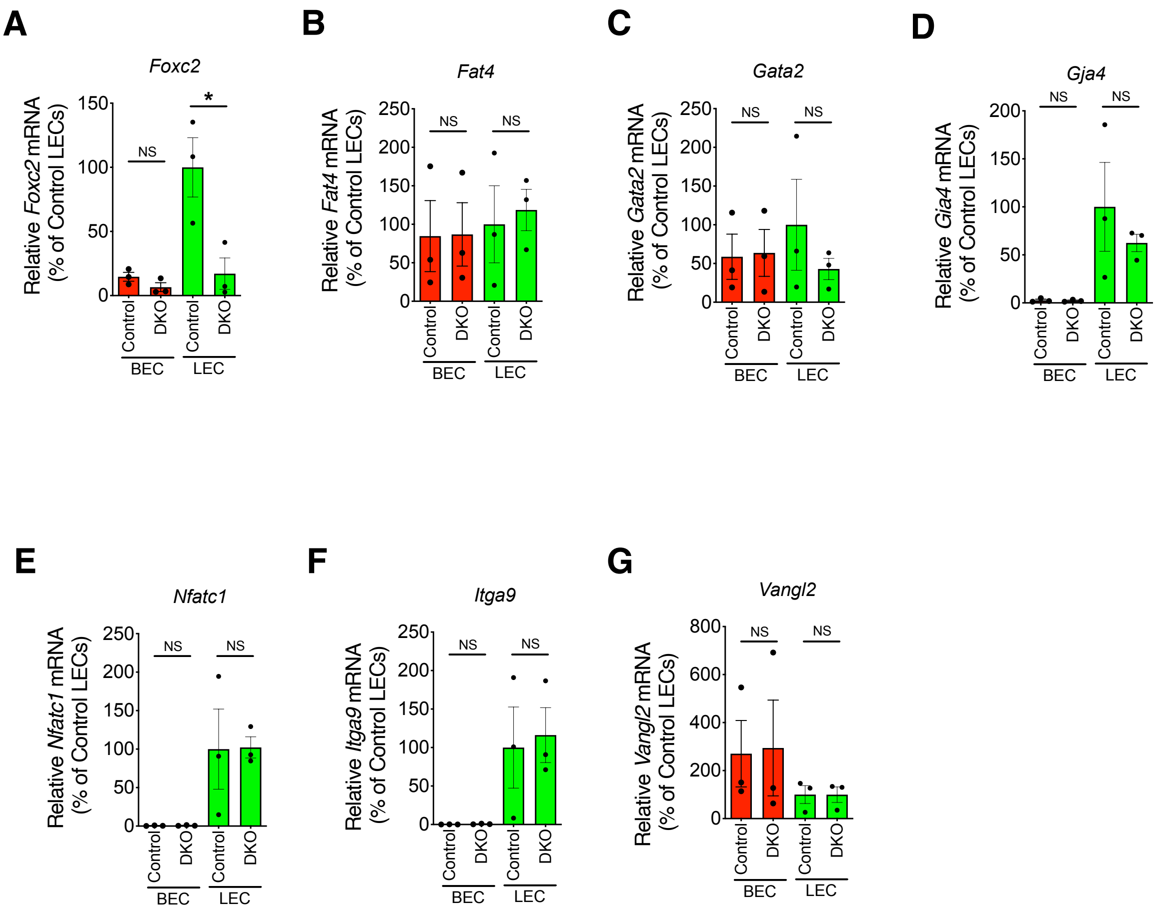

Supplemental Figure 14. *Lpa4*/*Lpa6* deletion reduces *Foxc2* expression in dorsal LECs from neonatal mice. (**A**–**G**) mRNA expression of lymphatic valve-related genes in FACS-sorted dorsal blood ECs (BECs; $CD31^+PDPN^-$ cells) and LECs ($CD31^+PDPN^+$ cells) from control and *Lpa4*;$Lpa6^{\Delta EC}$ mice at P1 detected via qRT-PCR. Data are presented as mean ± SEM (n = 3 mice). *P < 0.05, two-tailed unpaired Student's t-test. NS, not significant.

SUPPLEMENTAL FIGURE 15

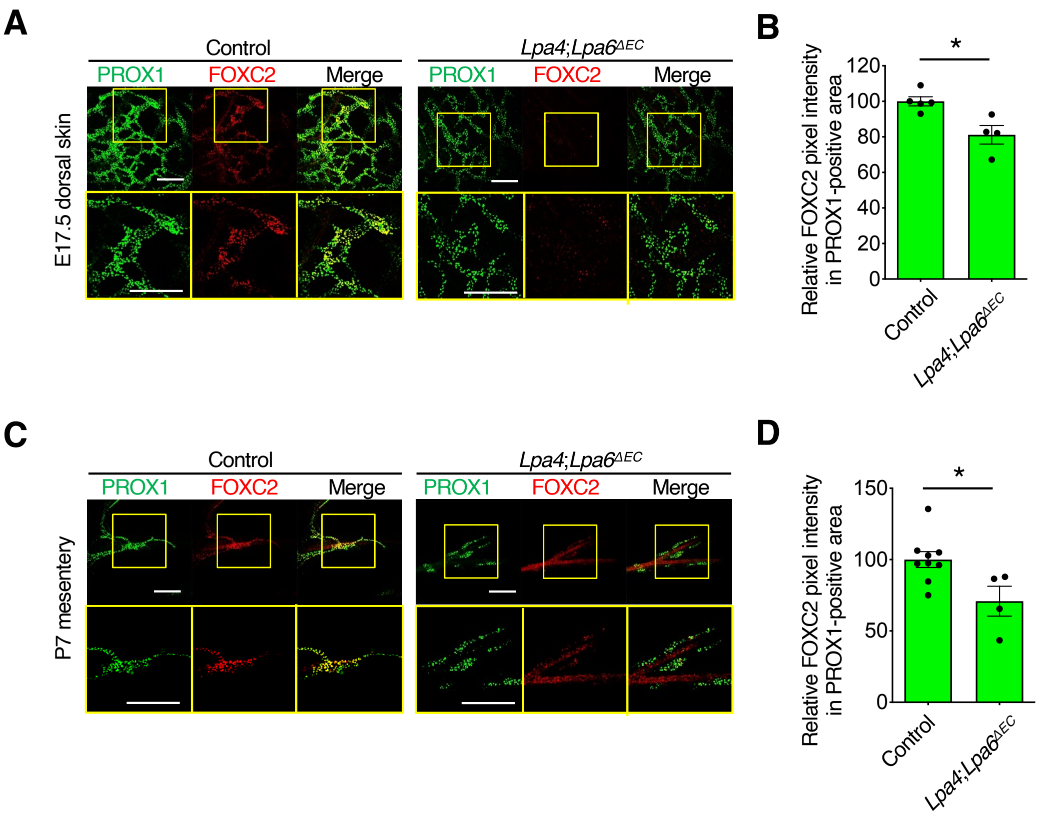

Supplemental Figure 15. ***Lpa4*;*Lpa6*$^{\Delta EC}$ mice decrease FOXC2 expression in lymphatic vessels.** (**A**) Representative confocal images of lymphatic vascular networks in the dorsal skin at E17.5. Triple immunostaining for CD31, PROX1, and FOXC2 is shown. Areas marked with yellow line boxes are magnified at the bottom. Scale bars, 200 μm. (**B**) Quantification of FOXC2 expression in the PROX1-positive area at E17.5 (n = 4–5 embryos). *P < 0.05, two-tailed unpaired Student's t-test. (**C**) Representative confocal images of mesenteric lymphatic vessels at P7. Triple immunostaining for CD31, PROX1, and FOXC2 is shown. Areas marked with yellow line boxes are magnified at the bottom. Scale bars, 200 μm. (**D**) Quantification of FOXC2 expression in the PROX1-positive area at P7 (n = 4–9 mice). *P < 0.05, two-tailed unpaired Student's t-test.

SUPPLEMENTAL FIGURE 16

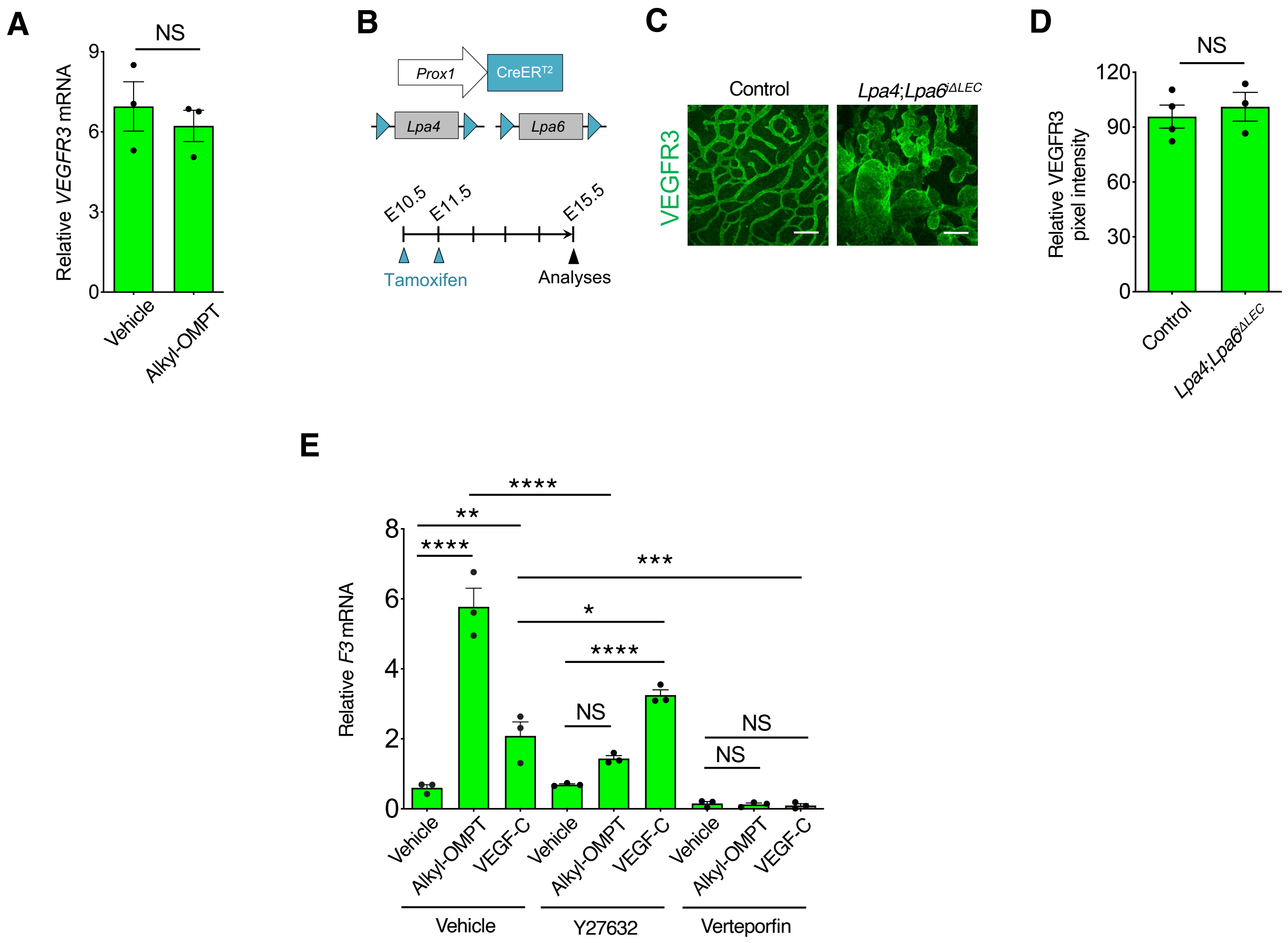

Supplemental Figure 16. Lymphatic endothelial *Lpa4/Lpa6* ablation does not affect VEGFR3 expression in mice. (**A**) *VEGFR3* mRNA expression unresponsive to alkyl-OMPT (10 μM, 3 hours) in serum-starved HMVECs-dNeo. Data are presented as mean ± SEM of triplicates. Two-tailed unpaired Student's *t*-test. NS, not significant. (**B**) Schematic diagram of *Lpa4* and *Lpa6* ablation in *Lpa4*;*Lpa6*$^{i\Delta LEC}$ mice and tamoxifen injection procedure for analysis at E15.5. (**C**) Representative confocal images of collective lymphatic vessels in the dorsal skin, showcasing VEGFR3 immunostaining. Scale bars, 200 μm. (**D**) Quantification of VEGFR3 mean fluorescence intensity in control and *Lpa4*;*Lpa6*$^{i\Delta LEC}$ embryos (n = 3 embryos). Two-tailed unpaired Student's *t*-test. NS, not significant. (**E**) *F3* mRNA induction in response to alkyl-OMPT (10 μM, 3 hours) or VEGF-C (100 ng/ml, 3 hours) was suppressed by Y27632 (10 μM, 1-hour pretreatment) and verteporfin (1 μM, 1-hour pretreatment) in serum-starved HMVECs-dNeo. Data are presented as mean ± SEM of triplicates. *P < 0.05, **P < 0.01, ***P < 0.001, ****P < 0.0001, one-way ANOVA followed by Tukey's multiple comparisons test. NS, not significant.

SUPPLEMENTAL FIGURE 17

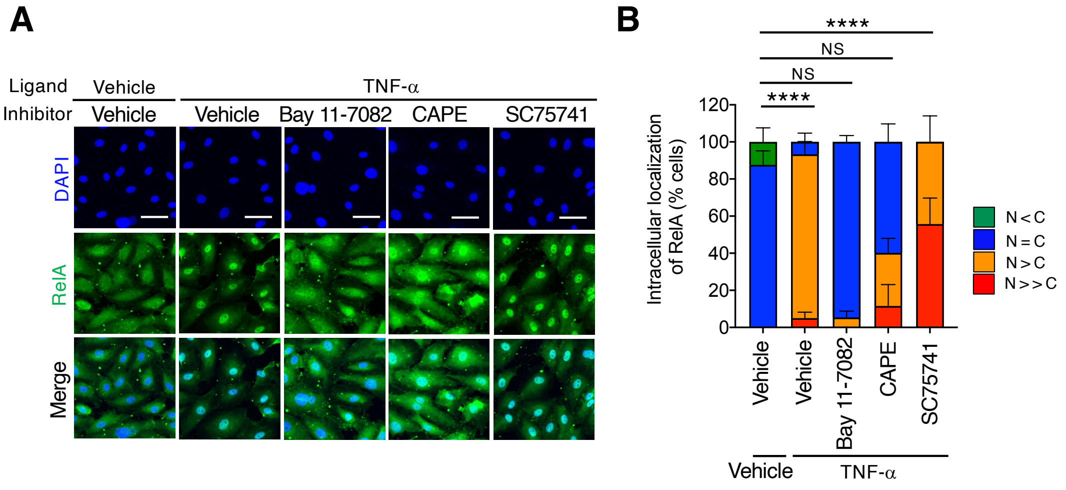

Supplemental Figure 17. IKK inhibitors reduce TNF-α-induced nuclear transport of RelA in LECs. (**A** and **B**) TNF-α (50 ng/ml, 1 hour)-induced RelA nuclear transport suppressed by IKK inhibitors Bay 11-7082 (5 μM, 1-hour pretreatment) and CAPE (30 μM, 1-hour pretreatment) but not by NF-κB-DNA binding inhibitor SC75741 (10 μM, 1 hour pretreatment) in serum-starved HMVECs-dNeo. Representative confocal images (**A**) and corresponding quantification of RelA intracellular localization (**B**) (*n* = 53–65 cells). Scale bars, 100 μm. ****$P < 0.0001$, one-way ANOVA followed by Dunnett's multiple comparisons test for $N = C$ ratio. NS, not significant.

SUPPLEMENTAL FIGURE 18

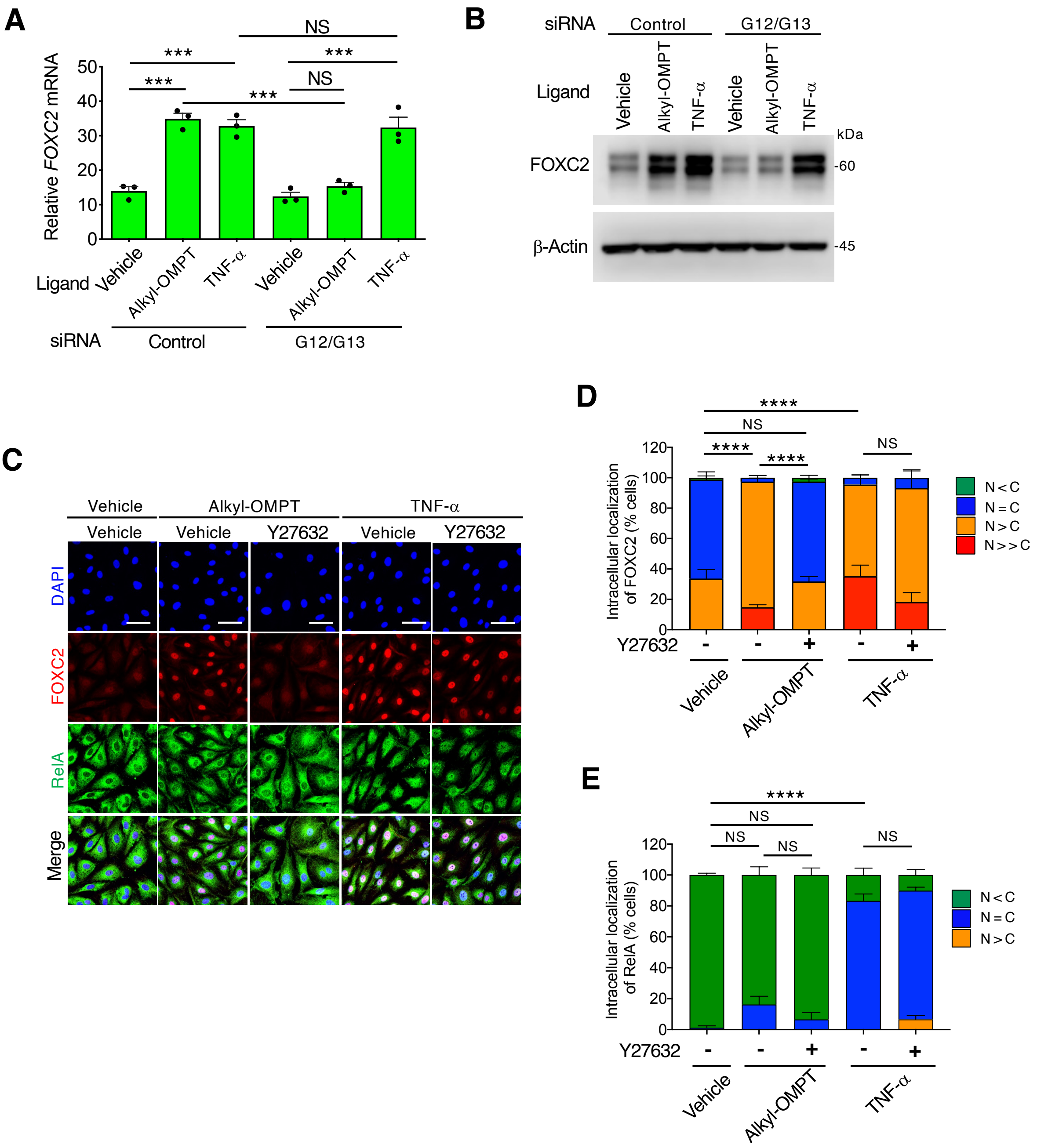

Supplemental Figure 18. TNF-α increases nuclear FOXC2 expression independent of Gα12/Gα13-ROCK signaling in LECs. (**A**) *GNA12/GNA13* siRNAs (48-hour pretreatment) suppressed *FOXC2* mRNA induction in response to alkyl-OMPT (10 μM, 3 hours) but not TNF-α (50 ng/ml, 3 hours) in serum-starved HMVECs-dNeo. Data are presented as mean ± SEM of triplicates. ***$P < 0.001$, one-way ANOVA followed by Tukey's multiple comparisons test. NS, not significant. (**B**) *GNA12/GNA13* siRNAs (48-hour pretreatment) suppressed FOXC2 protein induction in response to alkyl-OMPT (10 μM, 6 hours) but not TNF-α (50 ng/ml, 6 hours) in serum-starved HMVECs-dNeo. Unprocessed original western blot scans are shown in Supplemental Figure 28. (**C**–**E**) Y27632 (10 μM, 1-hour pretreatment) suppressed nuclear FOXC2 protein induction in response to alkyl-OMPT (10 μM, 5 hours) but not TNF-α (50 ng/ml, 5 hours) in serum-starved HMVECs-dNeo. Representative confocal images (**C**) and corresponding quantification of FOXC2 (**D**) and RelA (**E**) intracellular localization (*n* = 76–95 cells). Scale bars, 100 μm. ****$P < 0.0001$, one-way ANOVA followed by Tukey's multiple comparisons test for $N = C$ ratio. NS, not significant.

SUPPLEMENTAL FIGURE 19

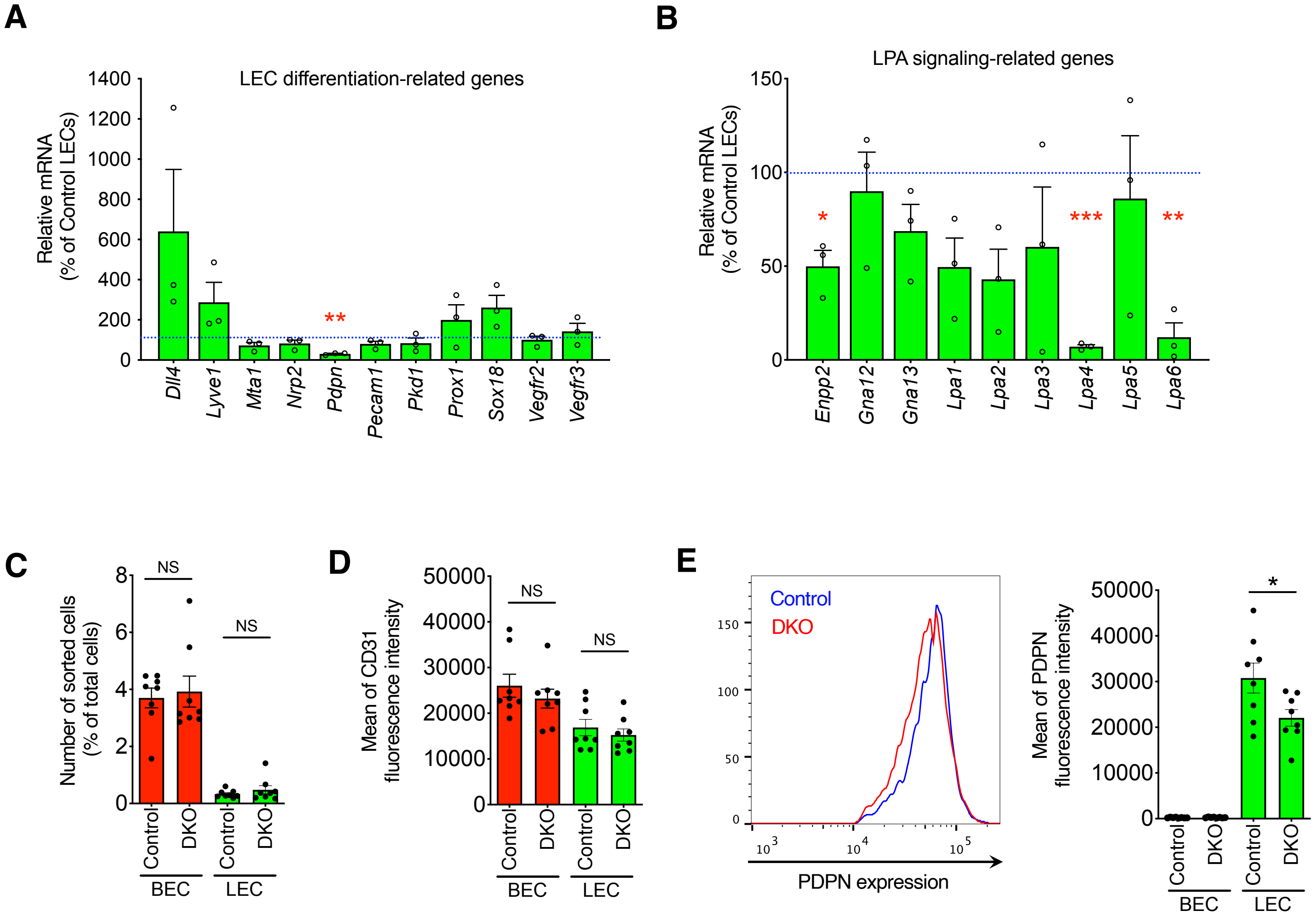

Supplemental Figure 19. *Lpa4*/*Lpa6* deletion reduces PDPN expression in mouse LECs. (**A** and **B**) mRNA expression of (**A**) LEC differentiation- and LPA signaling- (**B**) related genes in lung LECs isolated from control and *Lpa4*;*Lpa6*$^{\Delta EC}$ mice detected via qRT-PCR. Each gene expression level in LECs from *Lpa4*;*Lpa6*$^{\Delta EC}$ mice is normalized to that of control mice prepared in parallel. Data are presented as mean ± SEM (n = 3 sets of independent LECs prepared in parallel from control and *Lpa4*;*Lpa6*$^{\Delta EC}$ mice). **P* < 0.05, ***P* < 0.01, ****P* < 0.001, Welch's *t*-test. (**C**–**E**) Quantification of cell number (**C**) and expression levels of CD31 (**D**) and PDPN (**E**) in blood ECs (BECs; CD31$^+$ LYVE1$^-$ cells) and LECs (CD31$^+$ LYVE1$^+$ cells). Cells were isolated from the skin of control and *Lpa4*;*Lpa6*$^{\Delta EC}$ neonatal mice at P3 and subjected to flow cytometry (n = 8 mice). Representative histograms of PDPN expression levels in LECs isolated from control (*blue*) and *Lpa4*;*Lpa6*$^{\Delta EC}$ (*red*) mice are shown on the left in (**E**). Data are presented as mean ± SEM. **P* < 0.05, two-tailed unpaired Student's *t*-test. NS, not significant.

SUPPLEMENTAL FIGURE 20

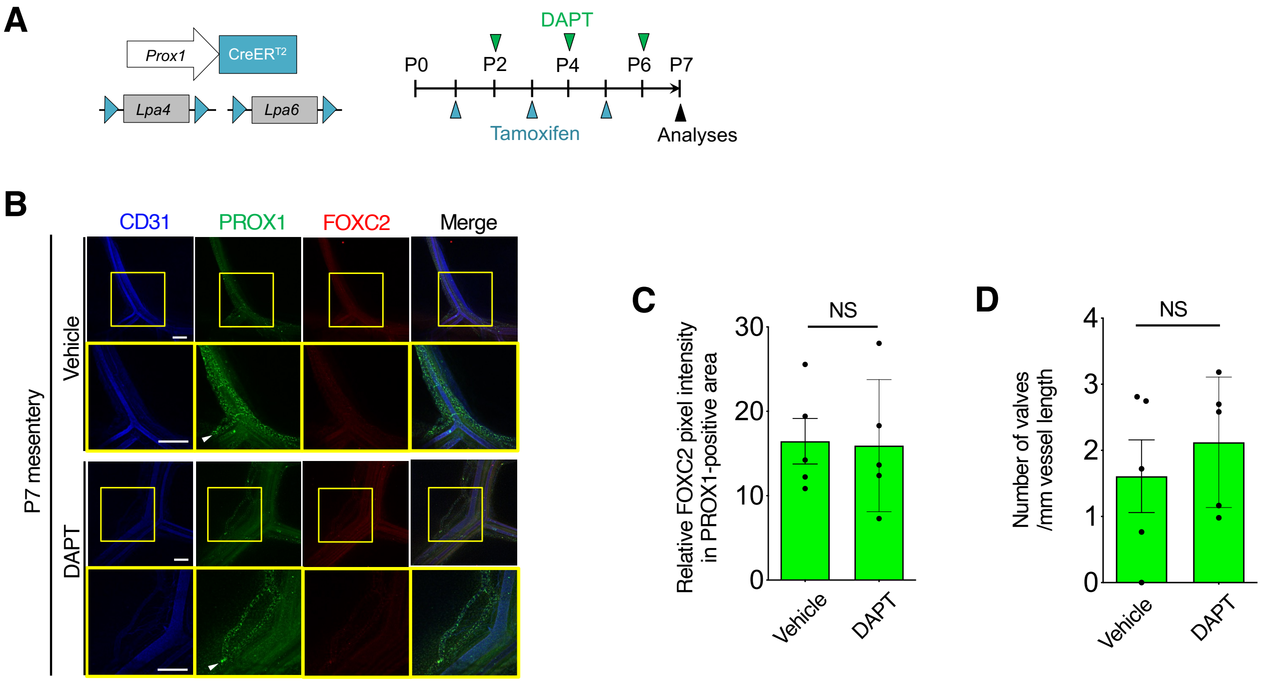

Supplemental Figure 20. The Notch signal inhibitor DAPT does not affect lymphatic valve number in mesenteric lymphatic vessels of *Lpa4*;*Lpa6*$^{i\Delta LEC}$ mice (**A**) Schematic diagram of DAPT (50 mg/kg) subcutaneously administration, and tamoxifen injection procedure at P7. (**B**) Representative confocal images of mesenteric lymphatic vessels in *Lpa4*;*Lpa6*$^{i\Delta LEC}$ mice treated with DAPT. Triple immunostaining for CD31, PROX1, and FOXC2 is shown. Areas marked by yellow line boxes are magnified at the bottom. White arrowheads indicate lymphatic valves. Scale bars, 200 µm. (**C** and **D**) Quantification of FOXC2 expression in the PROX1-positive area (**C**) and lymphatic valve number (**D**) (*n* = 5 mice). Two-tailed unpaired Student's *t*-test. NS, not significant.

SUPPLEMENTAL FIGURE 21

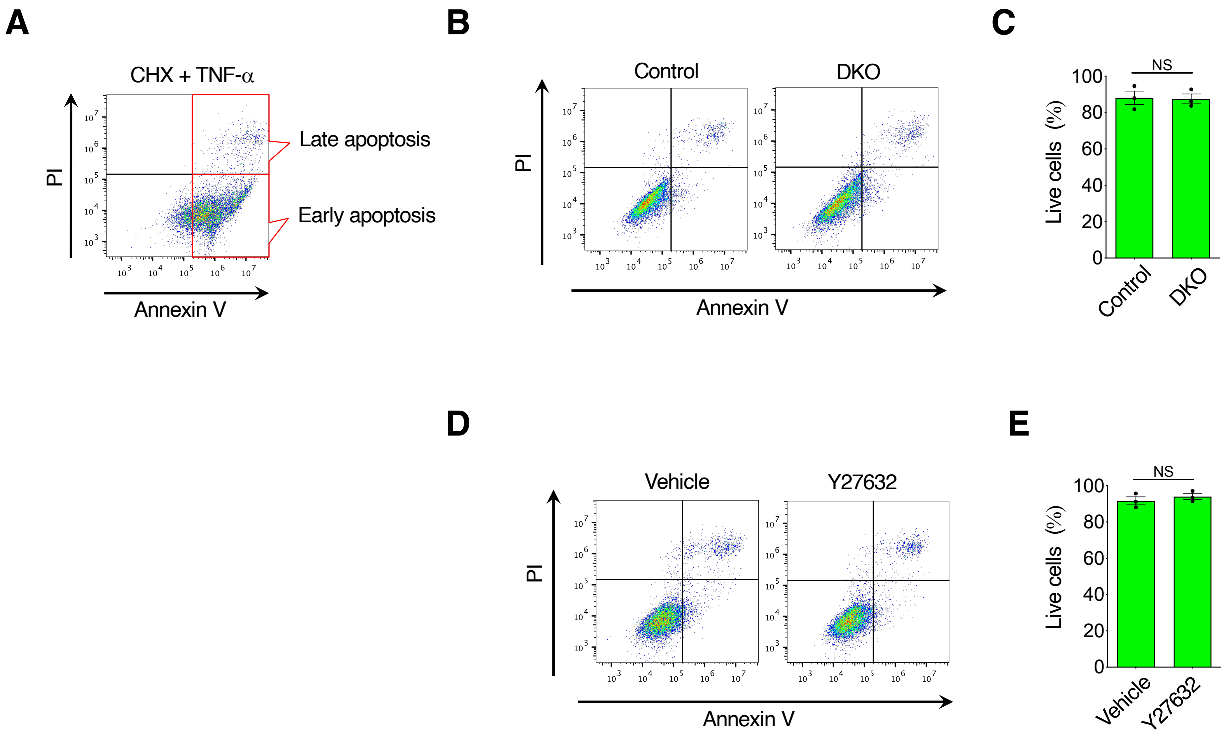

Supplemental Figure 21. *Lpa4*/*Lpa6* deletion does not promote LEC apoptosis. (**A**) A representative flow cytometry plot showing apoptosis in HMVECs-dNeo induced by cycloheximide (10 μg/ml) and TNF-α (50 ng/ml) treatment for 6 hours (*n* = 3). HMVEC-dNeo populations in early and late apoptotic stages were detected using propidium iodide (PI) and annexin V. (**B**) Effects of *Lpa4*/*Lpa6* deletion on apoptosis in mouse lung LECs. Control and DKO LECs were cultured for 24 hours. Representative flow cytometry plots are shown. (**C**) Quantification of live cell ratios. Data are presented as mean ± SEM (*n* = 3 sets of independent LECs prepared in parallel from control and *Lpa4*;$Lpa6^{\Delta EC}$ mice). Two-tailed unpaired Student's *t*-test. NS, not significant. (**D**) Effects of ROCK inhibition on apoptosis in HMVEC-dNeo. Cells were cultured with or without Y27632 (10 μM) for 24 hours. Representative flow cytometry plots are shown. (**E**) Quantification of live cell ratios. Data are presented as mean ± SEM (*n* = 3 independent experiments). Two-tailed unpaired Student's *t*-test. NS, not significant.

SUPPLEMENTAL FIGURE 22

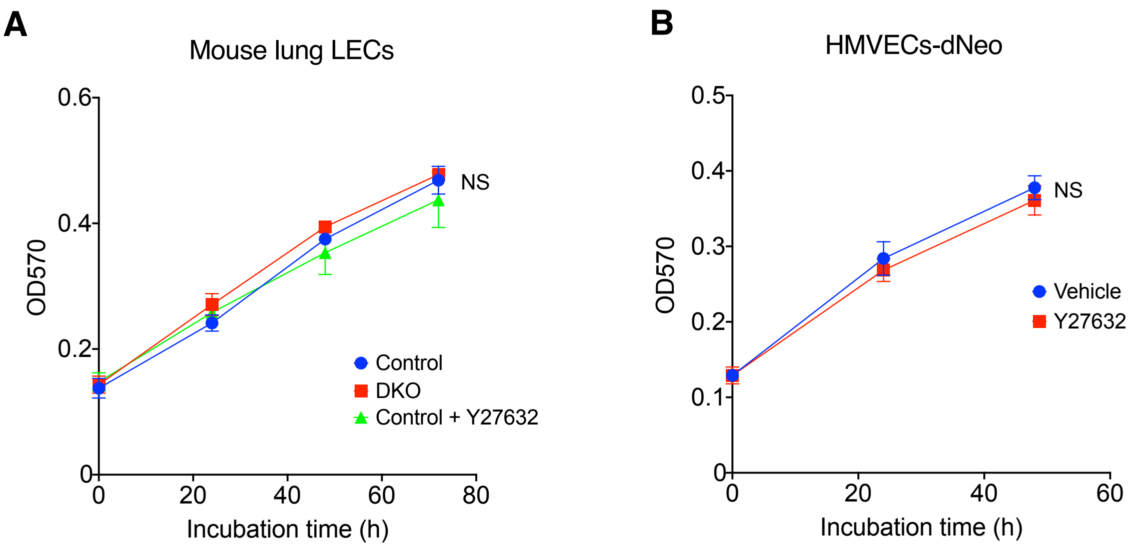

Supplemental Figure 22. *Lpa4*/*Lpa6* deletion does not affect LEC proliferation. (**A** and **B**) Effect of *Lpa4*/*Lpa6* deletion or Y27632 (10 μM) treatment on mouse lung LECs (**A**) and HMVECs-dNeo (**B**) proliferation. Data are presented as mean ± SEM of triplicates. Two-way ANOVA followed by Bonferroni's multiple comparison test. NS, not significant.

SUPPLEMENTAL FIGURE 23

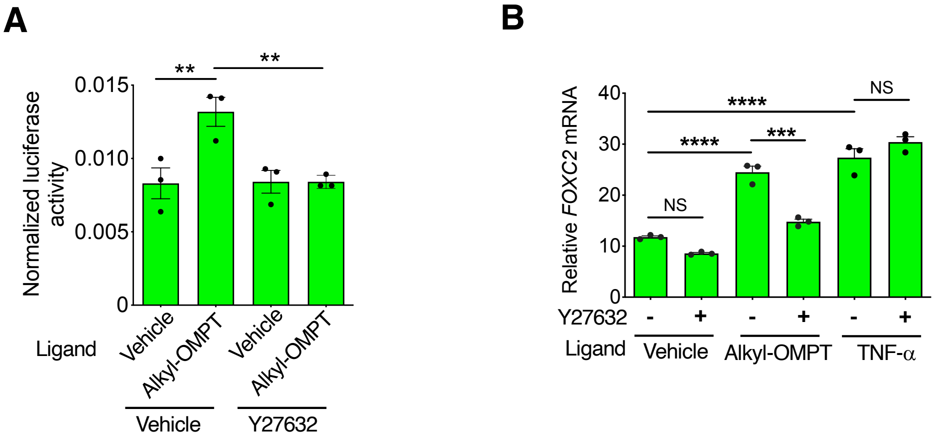

Supplemental Figure 23. LPA4/LPA6 signaling promotes FOXC2 expression by NF-κB activation through ROCK in HUVECs. (**A**) Increased NF-κB reporter activity in response to alkyl-OMPT (10 μM, 6 hours) was attenuated by Y27632 (10 μM, 1-hour pretreatment) in serum-starved HUVECs. Data are presented as mean ± SEM of triplicates. $^{**}P < 0.01$, one-way ANOVA followed by Tukey's multiple comparisons test. (**B**) Y27632 (10 μM, 1-hour pretreatment) suppressed *FOXC2* mRNA induction in response to alkyl-OMPT (10 μM, 3 hours) but not TNF-α (50 ng/ml, 3 hours) in serum-starved HUVECs. Data are presented as mean ± SEM of triplicates. $^{***}P < 0.001$, $^{****}P < 0.0001$, one-way ANOVA followed by Tukey's multiple comparisons test. NS, not significant.

SUPPLEMENTAL FIGURE 24

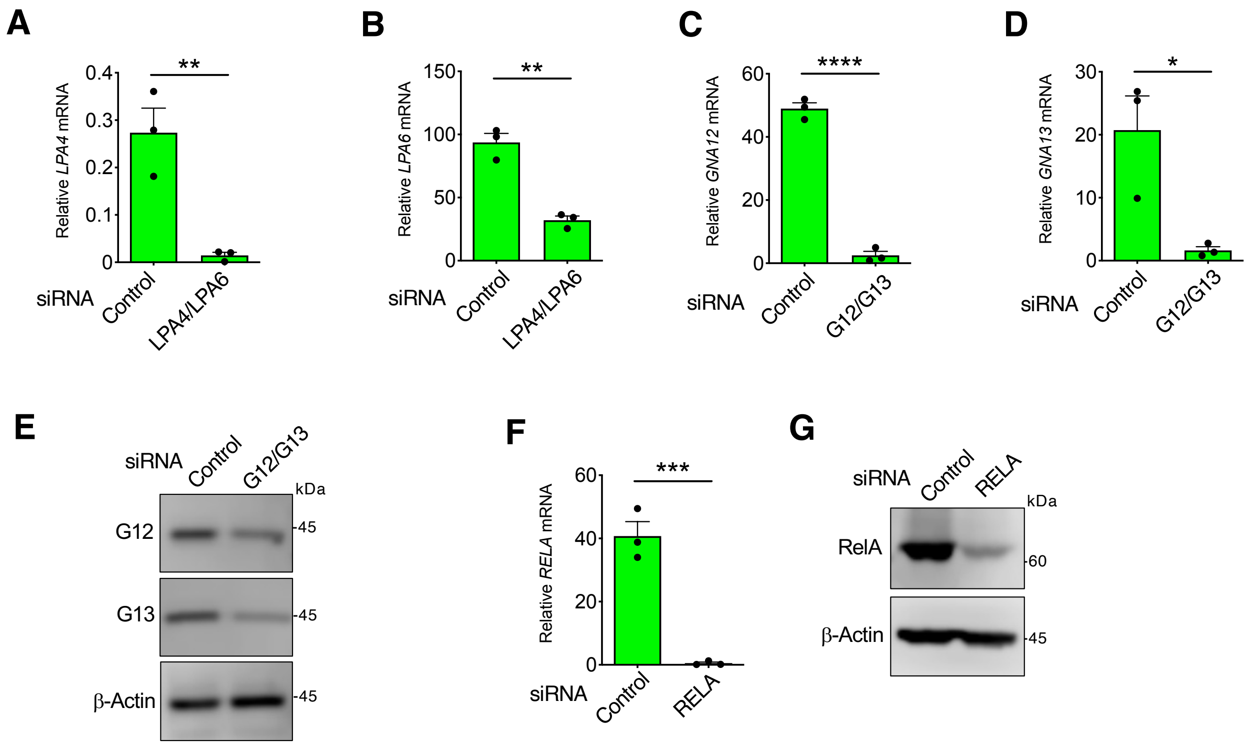

Supplemental Figure 24. Confirmation of mRNA and protein reduction by siRNAs in this study. mRNA and protein expression suppressed by siRNAs (48 hours) for LPA4/LPA6 (**A** and **B**), Gα12/Gα13 (**C**–**E**), and RelA (**F** and **G)** in HMVECs-dNeo. Data are presented as mean ± SEM of triplicate. **P* < 0.05, ***P* < 0.01, ****P* < 0.001, *****P* < 0.0001, two-tailed unpaired Student's *t*-test. Unprocessed original western blot scans are shown in Supplemental Figure 28.

SUPPLEMENTAL FIGURE 25

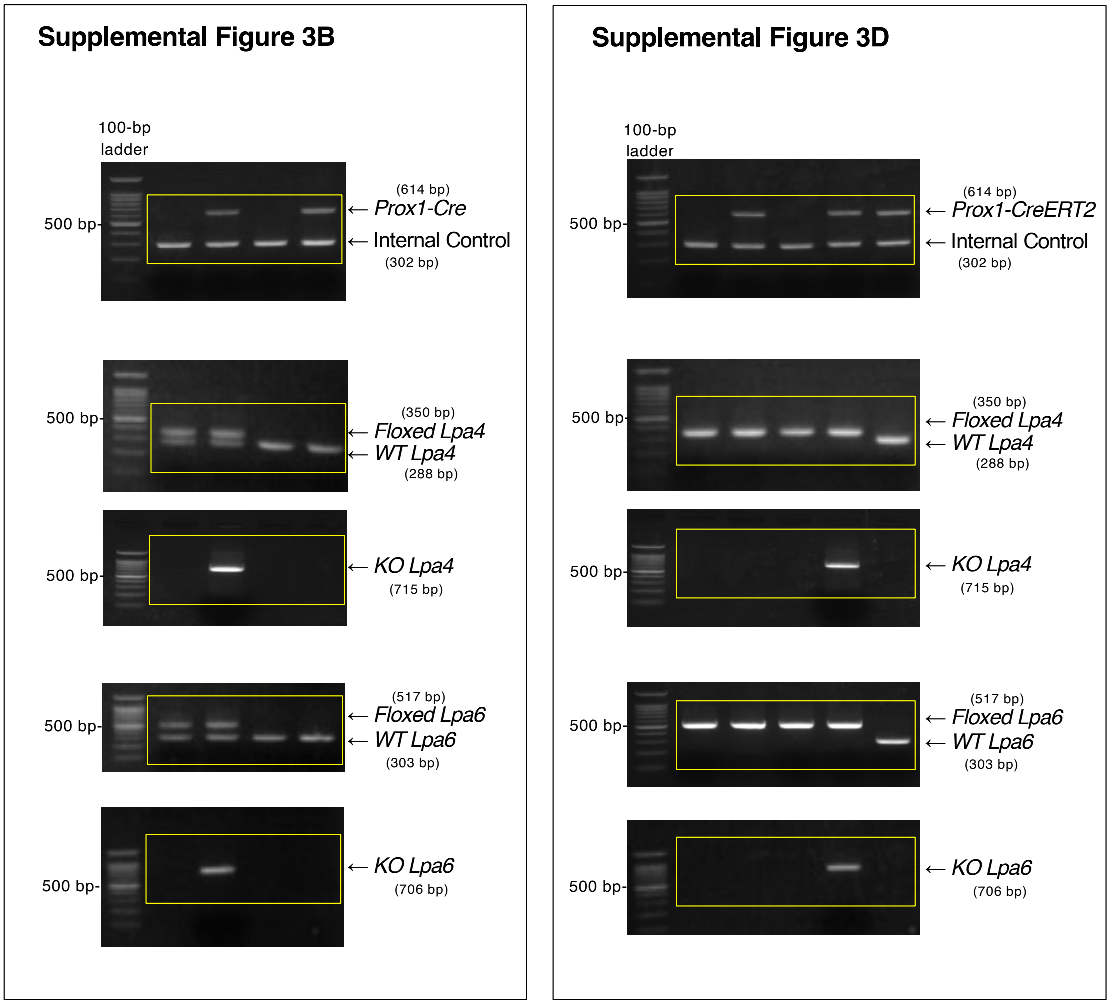

Supplemental Figure 25. Unprocessed original scans of PCR genotyping shown in Supplemental Figure 3B and 3D.

SUPPLEMENTAL FIGURE 26

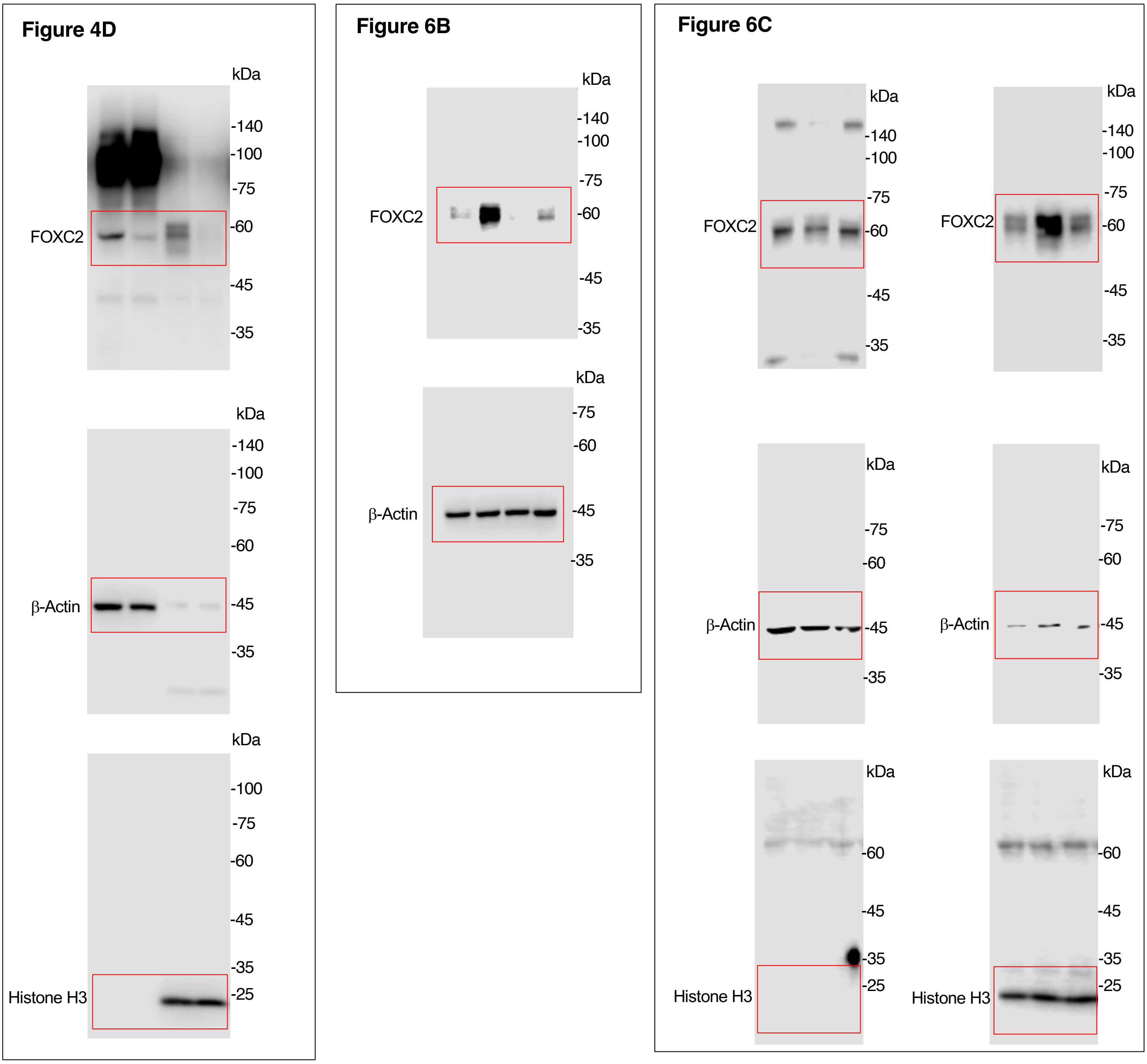

Supplemental Figure 26. Unprocessed original scans of western blots shown in Figure 4D, 6B, and 6C.

SUPPLEMENTAL FIGURE 27

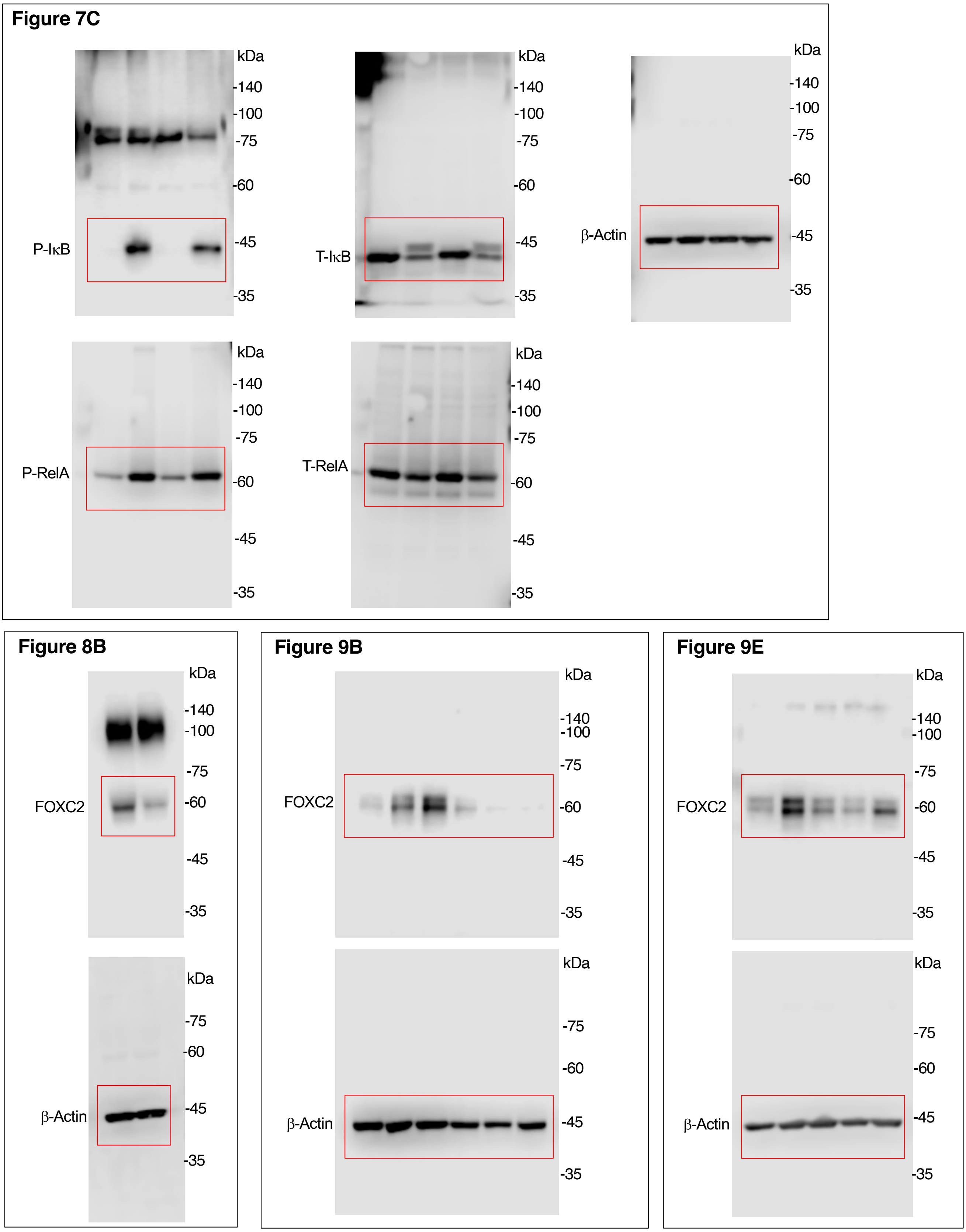

Supplemental Figure 27. Unprocessed original scans of western blots shown in Figure 7C, 8B, 9B, and 9E.

SUPPLEMENTAL FIGURE 28

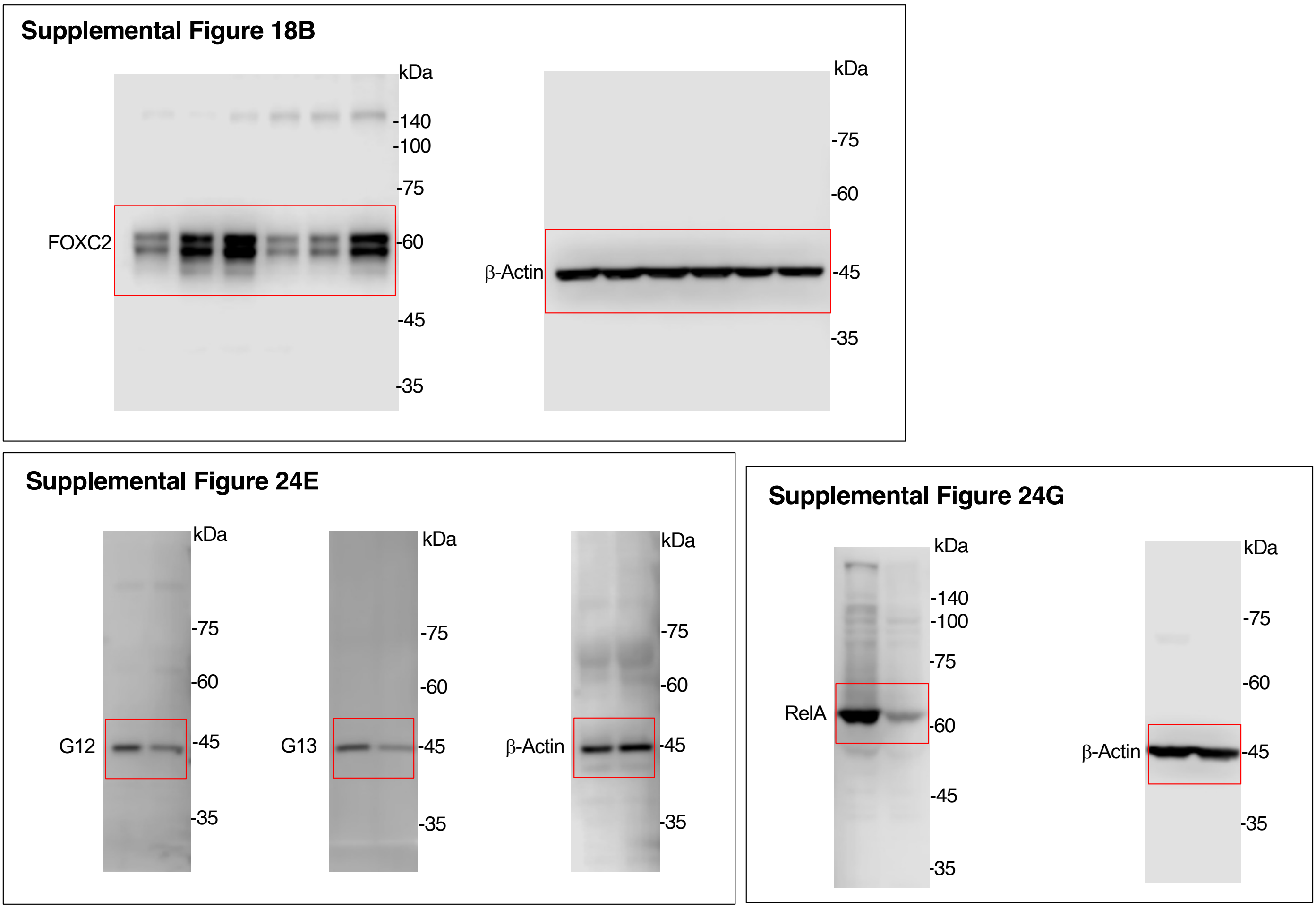

Supplemental Figure 28. Unprocessed original scans of western blots shown in Supplemental Figure 18B, 24E, and 24G.

SUPPLEMENTAL FIGURE 29

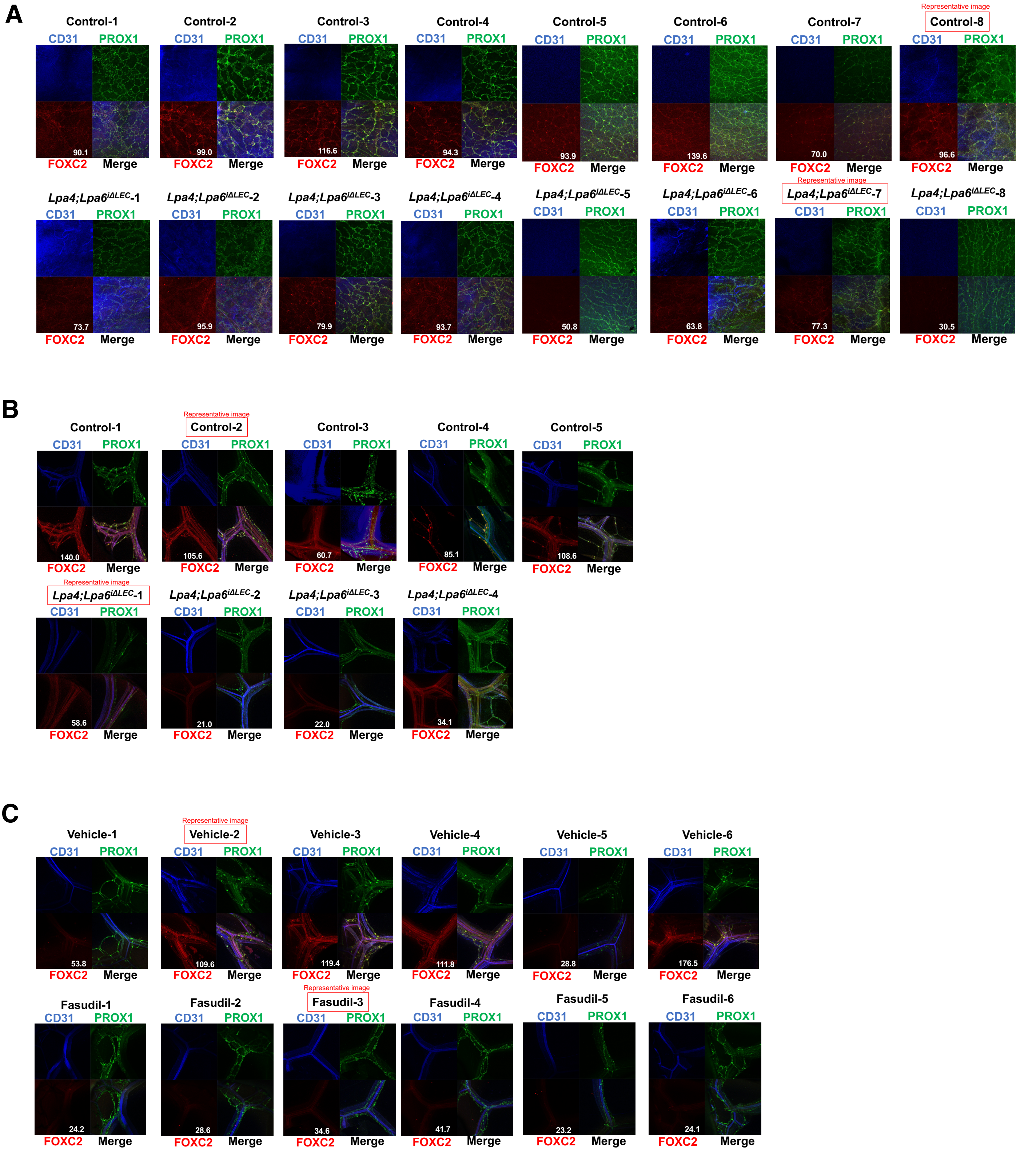

Supplemental Figure 29. Original images of all samples in Figure 5. (**A**) Confocal images of all samples in Figure 5B. (**B**) Confocal images of all samples in Figure 5D. (**C**) Confocal images of all samples in Figure 5G. White values indicate the relative FOXC2 pixel intensity in PROX1-positive regions.

Supplemental Table 1

The genomic sequence around the last exon of mouse *Prox1* and the sequences inserted into it to construct *pCre-Prox1* vector.

The last exon consisting of the CDS and 3'UTR is highlighted in yellow. The sequences used to construct *pCre-Prox1* vector (i.e., 5' arm region, 3' arm region, CRISPR target sites, GSGP2A, nls, Cre, and rGpA) are described as follows: 5' arm region, 3' arm region, CRISPR Target, GSGP2A, nls, Cre, and rGpA.

CCTAAATTACCCGACCCCCTCAAAAGCTCATGCTGTTGTAGAGTTATCTAGACAAATTCTGCTTCTGACA
GCTTCCACAAGGCCCTCCTCCCTGGCCCTGACTTCTTGGGGATAGGCTAGGCCCAGGTGGAGTTGG
GTGTGTCCTTGGAGTGGGTGGGACATCTCCCTGGCCCTGGCTGCCTGTGGATAGGCTAGGCCCAGG
TGGGGTTGGGCGTGTCCTTGGAGTGGGTGGGGCATCTGACTTACCCGCCCTTTGTAGCACTCACACC
TGTGTGTTTCCGAGCTCTCTGTCTCCCGTAGCTCTAGGTTTGTTGTTGAAGGAAGACAGGTCCCCATG
GAGGTTCTGTTGGCAGCAGCCAGCGAGAGAGCACACAGTTTGTACCGCTGATCTTTGTACGTGTTAGC
ATTTGGCTGCAGACCTACAGCCAGCCACATGGCCTCTCTCAGCAAGGCCCGTTTGGTATGACTTGTCA
TTGGGTGGGGACAGGATGCTCCCTCCAGGAAATCCGCATGTCTGCAGGTGCTTCCATGGAATCTCTG
TGGCTTCTCAGTAGACTGGTGTTTGTCCAGTAAGGGTGAAGGGGCCCTTGCTACACTTCCTGTCAGCT
CTGAGCTCTGTCTGGACAGGCCAACATAAACGCTTCGGGGAGCGATTGCTCAATAACCTCCTCTTTAC
TTCTGAGACGCAGGGTCTCAGTATAGCTCTGACTAGCCTGGAACTCAGATCCAAGTGCTCAGATTGAA
GATGTGGGCCATTGTGCCTGTTCTAGACTTGGTGTTGGGCCCTTTTCTTCCCTTTCCCTTTTTTCCTCC
TTTCCCTTTTTATTTATTTATTGATTTATTTTTCTGGAAAGTTACTAGATTTCCCTAAAGAGCAATTGACTAG
CTACTGGAAGAAATGAGAACTTCAGGGACATCCCGTGATTGTTTTAGTTTTCTATGTTGCCTATCACTCA
TTCATGTGTATGTATCTATGTATATGTATATCCATGTACATGCCCACTATACCTGCTGAGAAAAGGCAGAG
AGGGAGACACTGCACTTGTTCGTGGACAATTATCTCCCAGTAACTGGCATTTCTGAGCTGCAGTGAAG
GTCTTTCATGTTATATCTGTGAGGATAGAAGTGTCTACACATACACTGCCATGCATTCATGTCCTCCTAC
ATCGGAACTTGATGTAAAAATAAAACCTCACTAATGTGTACTGGGAAAATTATTAATGTGCAGTTATTTTAT
GAATCCATCACGCTGATATGAGCACTGAGAGGCCATGCTTTACTGTAAGTTGCTTCTGAAGAGTGCCAA
GTATTCTTCTCACAGTCAAAAGGTCACACTATTTAATCAGGAGAAGACAAAAGAAGACAGACAGAAAGA
CAGACAGACAGGTCTCATACATTAATTGCTATGGGAGCAAGGACTTGACAAATAGAAATTCCGAAAGGC
TTATTTGGAGAATGACAATCTTGGAGTAAAATGGCCCTGAATGAGTCCTTCTGATCTCTCAGTGCCTCT
GTTTTTTTTTTTCCCCCAAGGTATCATTGAGACAGTAACACCTTACACAGAGGTTGGAAAAATGAAATGA
CATTCACTATGTAATGTAACATGGTATCCGACACAGTAAACACTCAGTAAGTGTTAGCTTTTGTAACAATG
ATCAATTACACAAACTGCCATCTAAGAAACTATTCTAATGCCAAAGCAAAAGGTAGCAATTCAGGGTTCA

GCGTAAGTTTCATAGCTCAGGATTTTGTACTGTTATCTTACCCCTTTATAGTACTGGCTATTTTAATAAACA
ATTGCGCGTCCAGGCAACAGTTCTACAGCACAAACAGGAAACCGATTTTAACGTTTCTTCATTGACGTT
AGGGATTTAGTTCATGTAGAAGCCCAACATGTATTATATTCTGAAAATACATTATTTTTTCATAATGGGAGT
CCAAAGGAGAAAGAAATCAAATTCTGAAAAGGGAGATGGCAAGTTTCTTCTCGAACGGACCTGCTTAA
CCACGTGAGGACGTGGGTCTCTCTTCTACTGCAGCGTTCCACAGTGAAACGCTGGTGTTGGCGGGTC
CCATGTATCCTCTGCGCATGTCTCCTCTGAAACCCACGGAGCAGCCGCCGTCTATCACAATGCTGCTA
TATTTAACAACTCCACACAAGAGAATAGAGGCCGGCCATTTTTAGAAAATGCAGGTGCCGTCTAAAGCC
CCCTTCTGAGAGTGTGGACTTAGCTCAGTTTCCTTTGAAGAACTACAGACTTGAAACTGAAAACTTTAT
TAATACCATTGTCTCCTTGTATCAGCAGGTTCCAGAGAGATTCCTGGAAGTTGCGCAGATCACGTTACG
GGAGTTTTTCAATGCCATCATCGCGGGCAAAGATGTTGATCCTTCCTGGAAGAAGGCCATTTACAAGGT
CATCTGCAAGCTGGATAGTGAAGTTCCTGAGATTTTCAAATCCCCTAACTGCCTACAAGAACTCCTTCA
CGAGGGCCGCAAGCTTGGAAGCGGAGCCACCAACTTCTCCCTGCTGAAGCAGGCCGGCGACGTGG
AGGAGAACCCCGGCCCCATGGCACCCAAGAAGAAGAGAAAGGTTTCGAATTTACTGACCGTACACCA
AAATTTGCCTGCATTACCGGTCGATGCAACGAGTGATGAGGTTCGCAAGAACCTGATGGACATGTTCA
GGGATCGCCAGGCGTTTTCTGAGCATACCTGGAAAATGCTTCTGTCCGTTTGCCGGTCGTGGGCGGC
ATGGTGCAAGTTGAATAACCGGAAATGGTTTCCCGCAGAACCTGAAGATGTTCGCGATTATCTTCTAT
ATCTTCAGGCGCGCGGTCTGGCAGTAAAAACTATCCAGCAACATTTGGGCCAGCTAAACATGCTTCAT
CGTCGGTCCGGGCTGCCACGACCAAGTGACAGCAATGCTGTTTCACTGGTTATGCGGCGGATCCGA
AAAGAAAACGTTGATGCCGGTGAACGTGCAAAACAGGCTCTAGCGTTCGAACGCACTGATTTCGACC
AGGTTCGTTCACTCATGGAAAATAGCGATCGCTGCCAGGATATACGTAATCTGGCATTTCTGGGGATT
GCTTATAACACCCTGTTACGTATAGCCGAAATTGCCAGGATCAGGGTTAAAGATATCTCACGTACTGA
CGGTGGGAGAATGTTAATCCATATTGGCAGAACGAAAACGCTGGTTAGCACCGCAGGTGTAGAGAAG
GCACTTAGCCTGGGGGTAACTAAACTGGTCGAGCGATGGATTTCCGTCTCTGGTGTAGCTGATGATC
CGAATAACTACCTGTTTTGCCGGGTCAGAAAAAATGGTGTTGCCGCGCCATCTGCCACCAGCCAGCT
ATCAACTCGCGCCCTGGAAGGGATTTTTGAAGCAACTCATCGATTGATTTACGGCGCTAAGGATGACT
CTGGTCAGAGATACCTGGCCTGGTCTGGACACAGTGCCCGTGTCGGAGCCGCGCGAGATATGGCCC
GCGCTGGAGTTTCAATACCGGAGATCATGCAAGCTGGTGGCTGGACCAATGTAAATATTGTCATGAA
CTATATCCGTAACCTGGATAGTGAAACAGGGGCAATGGTGCGCCTGCTGGAAGATGGCGATTAGGAA
TTCACTCCTCAGGTGCAGGCTGCCTATCAGAAGGTGGTGGCTGGTGTGGCCAATGCCCTGGCTCACA
AATACCACTGAGATCTTTTTCCCTCTGCCAAAAATTATGGGGACATCATGAAGCCCCTTGAGCATCTGA
CTTCTGGCTAATAAAGGAAATTTATTTTCATTGCAATAGTGTGTTGGAATTTTTTGTGTCTCTCACTCGGA
AGGACATATGGGAGGGCAAATCATTTAAAACATCAGAATGAGTATTTGGTTTAGAGTTTGGCAACATATG
CCATATGCTGGCTGCCATGAACAAAGGTGGCTATAAAGAGGTCATCAGTATATGAAACAGCCCCCTGCT

GTCCATTCCTTATTCCATAGAAAAGCCTTGACTTGAGGTTAGATTTTTTTTATATTTTGTTTTGTGTTATTT
TTTTCTTTAACATCCCTAAAATTTTCCTTACATGTTTTACTAGCCAGATTTTTCCTCCTCTCCTGACTACTC
CCAGTCATAGCTGTCCCTCTTCTCTTATGAAGATCCCTCGACCTGCAGGGATCGGCCGCCACCGGCG
CGAGATTGCAACGCTCTTTTGAATGTATGGATAGAAGAATTCCCCCTTGGATGTCCAACTTCTCTGTGT
CTAGATTTTGGTTTTATATATTTGTGTGGGGGAGGCATGGATATGTTGTGATGTCAGCTGTTGGTTCCTG
CTCATCTCTGGGTTCCTTTTCTTTTCCTTCATCACAAGGGGATGGGATGGGCGTTTTCCTTTAGTTTACT
TTTGCCCAAGGCCCTTAATATTTGGAGACTTAAGGTAGGGTTGGTTTTCAGGAGAAGGGAATGTCAGC
CTGTGTAAAGTGCACGTGAGCTATGGAGTGAGCCAGTGACTGTGACACAGCTCACATGCATACCCCAT
GCTGTGTCTCCTGTTTCTCTAGGGTTAAGAAGTTCTGGAAGAACCCGAGGAAAACACTTCACACACTG
GAATACCTTGTTTGATTTGAGCCTGATAAGCAAGCTTTTCTTAAACATCCAAACCAATGCCCTCGGCAG
CTGGGTAGCTCACATCTCAAAAGTATCCTCAAAAGACAGAGTTTTAGAAAGATTGTCACCAAGTCCTTT
GTAAGAGAAATGGCATAAACCCCCAGACCCAAGTTTGTACAAAACCGACTCTCCTGAGACAAGGCTGG
GAACGCACGCAGGCCAACCTGCTGTCTCTGTGAACTTTAGAACAGGCTCGTGGTGAGATGTGAGCTT
GGCCGTGTGGACTTAGAGGAAACCCTGTGCTTTGTCACTAGTTGCTATTTACTTTGCTTCAGATATTAAA
AGGCATGAACTTTCCAAATGTATCCTTTGCTTGACTCTCTTTATACGCTTAGCATTGCTGTTGCTGTTTAA
CCTTCTTTCTTCTCCTAAAACACTAATTTCTAGATACCTTTCTACAGTAAGGAATTAAGAAGTCCCACTGA
CTACGAAACCCTTGCCCACTCTTGGAGCACGCTGGCAGCTGCTAGTGGACCTGATCTTTACTTCCTGT
GAGGACATCACCCAGGAATGGCTCATTTGCATATGGGTATGGAGGGTGGCTGTCTGTCTTTTCCCTCT
GGGTTGTAATATCTTAAAGCAGCTGACCAGGTCTCTTCTACTACAGAAGCTAATATTGACCCAAGTGTGT
ACAATAGGTTAGTTTGGGGTTACCCAAAAAGACACAACTCCAGCATACTGAGAAAGCCGACTTCTCTCT
CTCCCACTCTCAGATGGCTCTTTCCAAACACCCAGGTTTTGATAATGTACCACCTCCTACACGTGCAT
GCTAACTATCGCTCACGTTTGTTTTCTTAGCTGGATTTTTGTGTACGGGTGCAGTAAACAGCACTGTTC
ACTCTAAGAGTGTGATCCAGGCCCCTACTTCCCCTTAGACAATCTGATGTGCACACTGTAAAAATGACA
CAGCAACAGATCCAGTATTTAAAACATAAAGGACAGAAATGCCATTTCCCCTTAATTTCTTAAAAAATAAA
AATCAGCGTTTGACTTTGGAGGACTCGGGTCAGCCCTTGTGACTGTCTAGCTGGCTGCAACGCTGTAA
TTCAACATCATTTATAAATTCTGCTGATGGGCAGGGATGTATGAACACAATTATTGTCAGCACAAAGCCT
TAAAACCTGCTGACTTCAAGCTAAACTGTGAAGCCCTGCGAGGCCCAGCATCTTCCGGGAGACCAGA
AGGCCCCGGCAATACGGACAGAGTATGGATTAGCATGCTCAACACTGTCCACTCACTACAGTAGCATC
TCCTCGTCTCACGCACCCACTCCTGAATAGACAATTGTTAGAGTATATTTGTTATTTTTCTGGCTGTCTTT
AAAACAAAAAGGCCAAGGTAGTCAATCATACCAGTGAGATAAATGTTACAGATTAAACAGCAGAACCAG
GAACCCAATCAATACAAGTTATGGAAGTCTTTGACATGCTTCATTTGCAAAGCTGGGGCACTGTGGTTC
CTGCAAGGGTCCATCAGGGTCCTAGCTGTTCTGAGTCACATGCTAATCAGATCTCCCCCACGTTACCC
TTTGCAAGGTCAAAGGCAAAGCTGCAGTGTCGTCAGTTGGGTAGACAGATAGCTAGGTATCTTCGTAAT

CTGGGGCAAATAAATATTGACTCTAGACTTAATCTTGATTTTTAAAATATTGTTTTCCTCTGCCTTTCTGAT
TTAAATAGACAAATTTAGCAAAAAAAGAAAAATGTCTTTTCTACTGAATGTCAGTGCCCTTAGCTACCTAG
ACTCTCCTCCTGGTGTTCTCTGAGATGAACAGATGATCTCACTCTGTCAGCCTGAGGACAGACAGCTC
GATGACAGAAGCAGCTCTCCACAGCTTTGCACTGAGGTGGTTGCCCCTAGTCGAGCCACTGGATCAA
GCATCTCCCGGTGAGATAGTAGATGACAGAAGCCTGTCTTTCAATACCCCTACCACGCAGTTAGTTCTT
AAATAAAACAAAAACCACCAAATAAATAAATAAGAATAAAATAACCTAGTATTGTTCACATCTTAATTCAGT
TCTCACCAAGTGAAAACCGCTTTGTGCACAGGAGAAAGTTAACTTTTCGTGGTGGGAACCTGGGGAC
CATTTCTAAATGCTGTGTTGCAATTCCACATGGCCACTTCTTTTATGCTAATCCTGTATTATTTGTGTGTG
TGCACTCAAGGTGATAGTCTGTGCCTAGATCTAAACTACACCAGCGTGGGGATTTAAAAAAAAAATCCT
TCAAAACACCAGTTCTTTCCCCCCATAAGTACAAATGTCCTTGTGCCTTCTGTGGCTTTCTGTCTTCTTT
TCACTTTATTTCCAAGTACAGCTGCAATAAACACTAATTTCTTTTCCTGGCCGTTTGACATGATGTTGATA
GCTATGCATATTTTGCGTCTTTTTAAAACAAAGCGGGAGAATAATGTTTTTGAAGAAGAGAAATTTTTAGA
ACAGTTTGATACACCGCAAATTATTTTTTTTTCTCAATTGTCTGAGCAGCATTCGCGTTTTTGAGATTCTT
GTAGAAGCCGATTTTTTTGTAACTGTGGTGTAGCTCTCGTGTGTTATTAGCCTAATGAGAAGCACTATAG
AAGCAATATTTCATACCATGTGCAATGTGTGTGCAGAAGTGTGGGCGTGAGCACGCAGGCACACACAT
GTAAAAATATACATATGTATGCGTGTGAAGTGGAAAGCTTACCTTTTCTTATCTAGACTTTAAGAACCTATT
TTAGGCATTTGTTATGTTTTGTGTAAAGAATGTTCTATTTGCAACAACACGACCCTGACTTCTTACCGTC
TCTCTGGCAGTTCAATGGAGGTGTTTTGCATTAAATGGTAAAATTGCGTGGAAGATGTTAGGATCTAGTA
ATTATTTCAGTGAATGTTCATACCATATTCCTGAAGTTTGCTTTGTGCCTCTGAGTGTTACTTAATTGAAG
TATTTTATGTTTGAAGAATCCTTGTTACTGTGCTAGGAATATGGGTGACTATCATTTTTAAAATATTTTAAA
AACATCAAAACAAAAAAAAAACACTAAAGCAAAGGGGAACTTTTATAAGGAAATGTAAATATTTAACCTCA
TGGCCGTTGTTACGTAAGACAGACATGAGATCTTAATAAATAGCTACAGTCTCACAGCATCTGTTGAATT
TATTAGGGACATTACACTGACTGTACAGGCAGTTGAACGCATCTCAAAAGTCCTGCTCTTGCAAAAGAT
ACCAGTTTCTCTCTATCAGAGGGCAAGGGCAAGGATACAGGCGACTCTGTAAATCTATGACATTTCTTT
TCTGTGTACATGAAGACATTTAGTAAGTAATCCCCCAAGTCCCCACACATGCAGGTTAATATCAATCAAT
TAATTAAAGAGTTGGGGGTTAACCATAGGCTGCTCCCCAAAAGCAACCATAAATTAATCAAAGCCAGAG
TGGTTTACAGATATCCCCCCAAAAGCAGCAATGTGTGTAAGGACATGAACAATCCCTTTGCCATGTGTA
TTATAGAGAATTCATTGGTGTGAACAGCACAACCGTTTCCTACTGGTGTAAACTCCGTGTCTGCAGATT
TACAGCGTTGTTTTCAAAGGCTCCCTTCGGAACGTGTAACGGTTGGTTTGTGTCGCTGTGTCACACTT
ACACGCAAGGAAGCTGAGTTTTGCCTCTGTAGATCTGTTGGTTTTAAACCGTCTAAAAGACTAAAGCTA
AAATGCTCCTCTTCAGAGCTGAGGTCCAAGCCAGTGTTTAATCTTTGCATCCTTTTGAGTGATCCTTCT
AAGACTAAGTGGAAGCATGTGGACTTTGAGTCTTTTCAACCGAGCGCTCTCTCAAATCAGACACCCCC
TAATGTGCAGACACGGGAATGAGCAGGCAAATGGTTGGCCTGTCTTTGGGAAAACGGATTCACTTTTT

TAAATAACCAATTCCTCTGCATATGGGGGGGAATAAAGCAGTTTAAAGTTGTATATTGCCACTTCAAATTG

AGGGGAAAAATTTAATGTATTTGGAGTTGGTCTCAGATCTCTAAAAGAGTCAAAGGTCACAGCTTCCCC

CAACATTGTCCCAGCCATTTCTCATATGTATATAGTATAAACCGTGACAAACACTGCCTTTATATTATTTAG

CAATATGTTGTAAATAGCATTATGAAGCTCTTTTTTTGTAATAAAGACCCTATGATTTGAATATAGTACAATA

ACTGAGCTGATAAAGTCAATTTTTGAATTTTTTTTTAGCTAGAGGCAATTTCAATTGTGCATTTTTGTTATT

GTCTATTGTTCTGAAGACTGCATAATTTATTGGTTTAATTTATCCTAATTTATTTGATGAAGGTGTACAATTT

TGTATTACCAAGGATGTACTGTAATATTAATTGATAGGATTAAAAACAGTGAGACTCCCTGTCCATATTCA

AAAGAAAACAAAAAGGTGCCGTAGACAATTGATTTTAAAGTAAAAAGTAAAAACAATTTAGTTTGGCAGC

TACTAAATTTTAAAACAAGGAAAAAAAAAAAAAAACCTAAAGGACAATGTTGTCGTGGGGGGGCGGGGA

GGGTTTCTGTTGTGTGTTTTAAGCTTTTCTATATTCTCCGAACTTTGACCGTTTGCTTTGTACCACTAAA

GGGTGCAGTAGTCCAACTGCCTTGTGTGCCTTCCATCTTCTCCTAAACTGAATGTATGTGCAGTATATAT

GCAAGCTTGTGCAAAATAAAATATACATTACAAGCTCATGCCATTCTTTTGTTTTTTTTGTTTTTTTTGTTT

TTCTGAAAACCCGTGGCATTTTGCTCTTGGACCTATTAGGCTTTGATCTATAGGCCTGGAGATGTCTTTT

CTGTGTCTGGAGGCTTGCTTTCCGTGTAGAGTCTCTCATTCTGATTAACTTTGGTTGGCCTTCAGCTTG

AGGGGAGAGAAAGGTGGTTCCCGCTTGAGGAGATGCATCCTGGGTAAAGCACTTGTGATGTGAGGGT

TGGGACCTGGACCGACATAAAGACAGGTTTAGGAGCATGCATCTTCAGTCCCAGAACTTTTACAATAAG

ACAGGA

Supplemental Table 2

The genomic sequence around the last exon of mouse *Prox1* and the sequences inserted into it to construct *pCreER^{T2}-Prox1* vector.

The last exon consisting of the CDS and 3'UTR is highlighted in yellow. The sequences used to construct *pCreER^{T2}-Prox1* vector (i.e., 5' arm region, 3' arm region, CRISPR target sites, GSGP2A, CreER^{T2}, and rGpA) are described as follows: 5' arm region, 3' arm region, CRISPR Target, GSGP2A, CreER^{T2}, and rGpA.

CCTAAATTACCCGACCCCCTCAAAAGCTCATGCTGTTGTAGAGTTATCTAGACAAATTCTGCTTCTGACA
GCTTCCACAAGGCCCTCCTCCCTGGCCCTGACTTCTTGGGGATAGGCTAGGCCCAGGTGGAGTTGG
GTGTGTCCTTGGAGTGGGTGGGACATCTCCCTGGCCCTGGCTGCCTGTGGATAGGCTAGGCCCAGG
TGGGGTTGGGCGTGTCCTTGGAGTGGGTGGGGCATCTGACTTACCCGCCCTTTGTAGCACTCACACC
TGTGTGTTTCCGAGCTCTCTGTCTCCCGTAGCTCTAGGTTTGTTGTTGAAGGAAGACAGGTCCCCATG
GAGGTTCTGTTGGCAGCAGCCAGCGAGAGAGCACACAGTTTGTACCGCTGATCTTTGTACGTGTTAGC
ATTTGGCTGCAGACCTACAGCCAGCCACATGGCCTCTCTCAGCAAGGCCCGTTTGGTATGACTTGTCA
TTGGGTGGGGACAGGATGCTCCCTCCAGGAAATCCGCATGTCTGCAGGTGCTTCCATGGAATCTCTG
TGGCTTCTCAGTAGACTGGTGTTTGTCCAGTAAGGGTGAAGGGGCCCTTGCTACACTTCCTGTCAGCT
CTGAGCTCTGTCTGGACAGGCCAACATAAACGCTTCGGGGAGCGATTGCTCAATAACCTCCTCTTTAC
TTCTGAGACGCAGGGTCTCAGTATAGCTCTGACTAGCCTGGAACTCAGATCCAAGTGCTCAGATTGAA
GATGTGGGCCATTGTGCCTGTTCTAGACTTGGTGTTGGGCCCTTTTCTTCCCTTTCCCTTTTTTCCTCC
TTTCCCTTTTTATTTATTTATTGATTTATTTTTCTGGAAAGTTACTAGATTTCCCTAAAGAGCAATTGACTAG
CTACTGGAAGAAATGAGAACTTCAGGGACATCCCGTGATTGTTTTAGTTTTCTATGTTGCCTATCACTCA
TTCATGTGTATGTATCTATGTATATGTATATCCATGTACATGCCCACTATACCTGCTGAGAAAAGGCAGAG
AGGGAGACACTGCACTTGTTCGTGGACAATTATCTCCCAGTAACTGGCATTTCTGAGCTGCAGTGAAG
GTCTTTCATGTTATATCTGTGAGGATAGAAGTGTCTACACATACACTGCCATGCATTCATGTCCTCCTAC
ATCGGAACTTGATGTAAAAATAAAACCTCACTAATGTGTACTGGGAAAATTATTAATGTGCAGTTATTTTAT
GAATCCATCACGCTGATATGAGCACTGAGAGGCCATGCTTTACTGTAAGTTGCTTCTGAAGAGTGCCAA
GTATTCTTCTCACAGTCAAAAGGTCACACTATTTAATCAGGAGAAGACAAAAGAAGACAGACAGAAAGA
CAGACAGACAGGTCTCATACATTAATTGCTATGGGAGCAAGGACTTGACAAATAGAAATTCCGAAAGGC
TTATTTGGAGAATGACAATCTTGGAGTAAAATGGCCCTGAATGAGTCCTTCTGATCTCTCAGTGCCTCT
GTTTTTTTTTTTCCCCCAAGGTATCATTGAGACAGTAACACCTTACACAGAGGTTGGAAAAATGAAATGA
CATTCACTATGTAATGTAACATGGTATCCGACACAGTAAACACTCAGTAAGTGTTAGCTTTTGTAACAATG
ATCAATTACACAAACTGCCATCTAAGAAACTATTCTAATGCCAAAGCAAAAGGTAGCAATTCAGGGTTCA

GCGTAAGTTTCATAGCTCAGGATTTTGTACTGTTATCTTACCCCTTTATAGTACTGGCTATTTTAATAAACA
ATTGCGCGTCCAGGCAACAGTTCTACAGCACAAACAGGAAACCGATTTTAACGTTTCTTCATTGACGTT
AGGGATTTAGTTCATGTAGAAGCCCAACATGTATTATATTCTGAAAATACATTATTTTTTCATAATGGGAGT
CCAAAGGAGAAAGAAATCAAATTCTGAAAAGGGAGATGGCAAGTTTCTTCTCGAACGGACCTGCTTAA
CCACGTGAGGACGTGGGTCTCTCTTCTACTGCAGCGTTCCACAGTGAAACGCTGGTGTTGGCGGGTC
CCATGTATCCTCTGCGCATGTCTCCTCTGAAACCCACGGAGCAGCCGCCGTCTATCACAATGCTGCTA
TATTTAACAACTCCACACAAGAGAATAGAGGCCGGCCATTTTTAGAAAATGCAGGTGCCGTCTAAAGCC
CCCTTCTGAGAGTGTGGACTTAGCTCAGTTTCCTTTGAAGAACTACAGACTTGAAACTGAAAACTTTAT
TAATACCATTGTCTCCTTGTATCAGCAGGTTCCAGAGAGATTCCTGGAAGTTGCGCAGATCACGTTACG
GGAGTTTTTCAATGCCATCATCGCGGGCAAAGATGTTGATCCTTCCTGGAAGAAGGCCATTTACAAGGT
CATCTGCAAGCTGGATAGTGAAGTTCCTGAGATTTTCAAATCCCCTAACTGCCTACAAGAACTCCTTCA
CGAGGGCCGCAAGCTTGGAAGCGGAGCCACCAACTTCTCCCTGCTGAAGCAGGCCGGCGACGTGG
AGGAGAACCCCGGCCCCATGTCCAATTTACTGACCGTACACCAAAATTTGCCTGCATTACCGGTCGAT
GCAACGAGTGATGAGGTTCGCAAGAACCTGATGGACATGTTCAGGGATCGCCAGGCGTTTTCTGAGC
ATACCTGGAAAATGCTTCTGTCCGTTTGCCGGTCGTGGGCGGCATGGTGCAAGTTGAATAACCGGAAA
TGGTTTCCCGCAGAACCTGAAGATGTTCGCGATTATCTTCTATATCTTCAGGCGCGCGGTCTGGCAGTA
AAAACTATCCAGCAACATTTGGGCCAGCTAAACATGCTTCATCGTCGGTCCGGGCTGCCACGACCAAG
TGACAGCAATGCTGTTTCACTGGTTATGCGGCGGATCCGAAAAGAAAACGTTGATGCCGGTGAACGTG
CAAAACAGGCTCTAGCGTTCGAACGCACTGATTTCGACCAGGTTCGTTCACTCATGGAAAATAGCGAT
CGCTGCCAGGATATACGTAATCTGGCATTTCTGGGGATTGCTTATAACACCCTGTTACGTATAGCCGAAA
TTGCCAGGATCAGGGTTAAAGATATCTCACGTACTGACGGTGGGAGAATGTTAATCCATATTGGCAGAA
CGAAAACGCTGGTTAGCACCGCAGGTGTAGAGAAGGCACTTAGCCTGGGGGTAACTAAACTGGTCGA
GCGATGGATTTCCGTCTCTGGTGTAGCTGATGATCCGAATAACTACCTGTTTTGCCGGGTCAGAAAAA
TGGTGTTGCCGCGCCATCTGCCACCAGCCAGCTATCAACTCGCGCCCTGGAAGGGATTTTTGAAGCA
ACTCATCGATTGATTTACGGCGCTAAGGATGACTCTGGTCAGAGATACCTGGCCTGGTCTGGACACAG
TGCCCGTGTCGGAGCCGCGCGAGATATGGCCCGCGCTGGAGTTTCAATACCGGAGATCATGCAAGCT
GGTGGCTGGACCAATGTAAATATTGTCATGAACTATATCCGTAACCTGGATAGTGAAACAGGGGCAATG
GTGCGCCTGCTGGAAGATGGCGATCTCGAGCCATCTGCTGGAGACATGAGAGCTGCCAACCTTTGGC
CAAGCCCGCTCATGATCAAACGCTCTAAGAAGAACAGCCTGGCCTTGTCCCTGACGGCCGACCAGAT
GGTCAGTGCCTTGTTGGATGCTGAGCCCCCCATACTCTATTCCGAGTATGATCCTACCAGACCCTTCAG
TGAAGCTTCGATGATGGGCTTACTGACCAACCTGGCAGACAGGGAGCTGGTTCACATGATCAACTGG
GCGAAGAGGGTGCCAGGCTTTGTGGATTTGACCCTCCATGATCAGGTCCACCTTCTAGAATGTGCCTG
GCTAGAGATCCTGATGATTGGTCTCGTCTGGCGCTCCATGGAGCACCCAGTGAAGCTACTGTTTGCTC

CTAACTTGCTCTTGGACAGGAACCAGGGAAAATGTGTAGAGGGCATGGTGGAGATCTTCGACATGCTG
CTGGCTACATCATCTCGGTTCCGCATGATGAATCTGCAGGGAGAGGAGTTTGTGTGCCTCAAATCTATT
ATTTTGCTTAATTCTGGAGTGTACACATTTCTGTCCAGCACCCTGAAGTCTCTGGAAGAGAAGGACCAT
ATCCACCGAGTCCTGGACAAGATCACAGACACTTTGATCCACCTGATGGCCAAGGCAGGCCTGACCC
TGCAGCAGCAGCACCAGCGGCTGGCCCAGCTCCTCCTCATCCTCTCCCACATCAGGCACATGAGTAA
CAAAGGCATGGAGCATCTGTACAGCATGAAGTGCAAGAACGTGGTGCCCCTCTATGACCTGCTGCTG
GAGGCGGCGGACGCCCACCGCCTACATGCGCCCACTAGCCGTGGAGGGGCATCCGTGGAGGAGAC
GGACCAAAGCCACTTGGCCACTGCGGGCTCTACTTCATCGCATTCCTTGCAAAAGTATTACATCACGG
GGGAGGCAGAGGGTTTCCCTGCCACAGCTTGAGAATTCACTCCTCAGGTGCAGGCTGCCTATCAGAA
GGTGGTGGCTGGTGTGGCCAATGCCCTGGCTCACAAATACCACTGAGATCTTTTTCCCTCTGCCAAAA
ATTATGGGGACATCATGAAGCCCCTTGAGCATCTGACTTCTGGCTAATAAAGGAAATTTATTTTCATTGC
AATAGTGTGTTGGAATTTTTTGTGTCTCTCACTCGGAAGGACATATGGGAGGGCAAATCATTTAAAACAT
CAGAATGAGTATTTGGTTTAGAGTTTGGCAACATATGCCATATGCTGGCTGCCATGAACAAAGGTGGCT
ATAAAGAGGTCATCAGTATATGAAACAGCCCCCTGCTGTCCATTCCTTATTCCATAGAAAAGCCTTGACT
TGAGGTTAGATTTTTTTTATATTTTGTTTTGTGTTATTTTTTTCTTTAACATCCCTAAAATTTTCCTTACATG
TTTTACTAGCCAGATTTTTCCTCCTCTCCTGACTACTCCCAGTCATAGCTGTCCCTCTTCTCTTATGAAG
ATCCCTCGACCTGCAGGGATCGGCCGCCACCGGCGCGAGATTGCAACGCTCTTTTGAATGTATGGAT
AGAAGAATTCCCCCTTGGATGTCCAACTTCTCTGTGTCTAGATTTTGGTTTTATATATTTGTGTGGGGGA
GGCATGGATATGTTGTGATGTCAGCTGTTGGTTCCTGCTCATCTCTGGGTTCCTTTTCTTTTCCTTCATC
ACAAGGGGATGGGATGGGCGTTTTCCTTTAGTTTACTTTTGCCCAAGGCCCTTAATATTTGGAGACTTA
AGGTAGGGTTGGTTTTCAGGAGAAGGGAATGTCAGCCTGTGTAAAGTGCACGTGAGCTATGGAGTGA
GCCAGTGACTGTGACACAGCTCACATGCATACCCCATGCTGTGTCTCCTGTTTCTCTAGGGTTAAGAA
GTTCTGGAAGAACCCGAGGAAAACACTTCACACACTGGAATACCTTGTTTGATTTGAGCCTGATAAGCA
AGCTTTTCTTAAACATCCAAACCAATGCCCTCGGCAGCTGGGTAGCTCACATCTCAAAAGTATCCTCAA
AAGACAGAGTTTTAGAAAGATTGTCACCAAGTCCTTTGTAAGAGAAATGGCATAAACCCCCAGACCCAA
GTTTGTACAAAACCGACTCTCCTGAGACAAGGCTGGGAACGCACGCAGGCCAACCTGCTGTCTCTGT
GAACTTTAGAACAGGCTCGTGGTGAGATGTGAGCTTGGCCGTGTGGACTTAGAGGAAACCCTGTGCT
TTGTCACTAGTTGCTATTTACTTTGCTTCAGATATTAAAAGGCATGAACTTTCCAAATGTATCCTTTGCTT
GACTCTCTTTATACGCTTAGCATTGCTGTTGCTGTTTAACCTTCTTTCTTCTCCTAAAACACTAATTTCTA
GATACCTTTCTACAGTAAGGAATTAAGAAGTCCCACTGACTACGAAACCCTTGCCCACTCTTGGAGCAC
GCTGGCAGCTGCTAGTGGACCTGATCTTTACTTCCTGTGAGGACATCACCCAGGAATGGCTCATTTGC
ATATGGGTATGGAGGGTGGCTGTCTGTCTTTTCCCTCTGGGTTGTAATATCTTAAAGCAGCTGACCAGG
TCTCTTCTACTACAGAAGCTAATATTGACCCAAGTGTGTACAATAGGTTAGTTTGGGGTTACCCAAAAAG

ACACAACTCCAGCATACTGAGAAAGCCGACTTCTCTCTCTCCCACTCTCAGATGGCTCTTTCCAAACA
CCCAGGTTTTTGATAATGTACCACCTCCTACACGTGCATGCTAACTATCGCTCACGTTTGTTTTCTTAGC
TGGATTTTTGTGTACGGGTGCAGTAAACAGCACTGTTCACTCTAAGAGTGTGATCCAGGCCCCTACTTC
CCCTTAGACAATCTGATGTGCACACTGTAAAAATGACACAGCAACAGATCCAGTATTTAAAACATAAAGG
ACAGAAATGCCATTTCCCCTTAATTTCTTAAAAAATAAAAATCAGCGTTTGACTTTGGAGGACTCGGGTC
AGCCCTTGTGACTGTCTAGCTGGCTGCAACGCTGTAATTCAACATCATTTATAAATTCTGCTGATGGGC
AGGGATGTATGAACACAATTATTGTCAGCACAAAGCCTTAAAACCTGCTGACTTCAAGCTAAACTGTGA
AGCCCTGCGAGGCCCAGCATCTTCCGGGAGACCAGAAGGCCCCGGCAATACGGACAGAGTATGGATT
AGCATGCTCAACACTGTCCACTCACTACAGTAGCATCTCCTCGTCTCACGCACCCACTCCTGAATAGA
CAATTGTTAGAGTATATTTGTTATTTTTCTGGCTGTCTTTAAAACAAAAAGGCCAAGGTAGTCAATCATAC
CAGTGAGATAAATGTTACAGATTAAACAGCAGAACCAGGAACCCAATCAATACAAGTTATGGAAGTCTTT
GACATGCTTCATTTGCAAAGCTGGGGCACTGTGGTTCCTGCAAGGGTCCATCAGGGTCCTAGCTGTTC
TGAGTCACATGCTAATCAGATCTCCCCCACGTTACCCTTTGCAAGGTCAAAGGCAAAGCTGCAGTGTC
GTCAGTTGGGTAGACAGATAGCTAGGTATCTTCGTAATCTGGGGCAAATAAATATTGACTCTAGACTTAA
TCTTGATTTTTAAAATATTGTTTTCCTCTGCCTTTCTGATTTAAATAGACAAATTTAGCAAAAAAAGAAAAA
TGTCTTTTCTACTGAATGTCAGTGCCCTTAGCTACCTAGACTCTCCTCCTGGTGTTCTCTGAGATGAAC
AGATGATCTCACTCTGTCAGCCTGAGGACAGACAGCTCGATGACAGAAGCAGCTCTCCACAGCTTTG
CACTGAGGTGGTTGCCCCTAGTCGAGCCACTGGATCAAGCATCTCCCGGTGAGATAGTAGATGACAGA
AGCCTGTCTTTCAATACCCCTACCACGCAGTTAGTTCTTAAATAAAACAAAAACCACCAAATAAATAAATA
AGAATAAAATAACCTAGTATTGTTCACATCTTAATTCAGTTCTCACCAAGTGAAAACCGCTTTGTGCACA
GGAGAAAGTTAACTTTTCGTGGTGGGAACCTGGGGACCATTTCTAAATGCTGTGTTGCAATTCCACATG
GCCACTTCTTTTATGCTAATCCTGTATTATTTGTGTGTGTGCACTCAAGGTGATAGTCTGTGCCTAGATC
TAAACTACACCAGCGTGGGGATTTAAAAAAAAAATCCTTCAAAACACCAGTTCTTTCCCCCCATAAGTAC
AAATGTCCTTGTGCCTTCTGTGGCTTTCTGTCTTCTTTTCACTTTATTTCCAAGTACAGCTGCAATAAAC
ACTAATTTCTTTTCCTGGCCGTTTGACATGATGTTGATAGCTATGCATATTTTGCGTCTTTTTAAAACAAA
GCGGGAGAATAATGTTTTTGAAGAAGAGAAATTTTTAGAACAGTTTGATACACCGCAAATTATTTTTTTTT
CTCAATTGTCTGAGCAGCATTCGCGTTTTTGAGATTCTTGTAGAAGCCGATTTTTTTGTAACTGTGGTGT
AGCTCTCGTGTGTTATTAGCCTAATGAGAAGCACTATAGAAGCAATATTTCATACCATGTGCAATGTGTG
TGCAGAAGTGTGGGCGTGAGCACGCAGGCACACACATGTAAAAATATACATATGTATGCGTGTGAAGT
GGAAAGCTTACCTTTTCTTATCTAGACTTTAAGAACCTATTTTAGGCATTTGTTATGTTTTGTGTAAAGAAT
GTTCTATTTGCAACAACACGACCCTGACTTCTTACCGTCTCTCTGGCAGTTCAATGGAGGTGTTTTGCA
TTAAATGGTAAAATTGCGTGGAAGATGTTAGGATCTAGTAATTATTTCAGTGAATGTTCATACCATATTCCT
GAAGTTTGCTTTGTGCCTCTGAGTGTTACTTAATTGAAGTATTTTATGTTTGAAGAATCCTTGTTACTGTG

CTAGGAATATGGGTGACTATCATTTTTAAAATATTTTAAAAACATCAAAACAAAAAAAAAACACTAAAGCA
AAGGGGAACTTTTATAAGGAAATGTAAATATTTAACCTCATGGCCGTTGTTACGTAAGACAGACATGAGA
TCTTAATAAATAGCTACAGTCTCACAGCATCTGTTGAATTTATTAGGGACATTACACTGACTGTACAGGCA
GTTGAACGCATCTCAAAAGTCCTGCTCTTGCAAAAGATACCAGTTTCTCTCTATCAGAGGGCAAGGGC
AAGGATACAGGCGACTCTGTAAATCTATGACATTTCTTTTCTGTGTACATGAAGACATTTAGTAAGTAATC
CCCCAAGTCCCCACACATGCAGGTTAATATCAATCAATTAATTAAAGAGTTGGGGGTTAACCATAGGCTG
CTCCCCAAAAGCAACCATAAATTAATCAAAGCCAGAGTGGTTTACAGATATCCCCCCAAAAGCAGCAAT
GTGTGTAAGGACATGAACAATCCCTTTGCCATGTGTATTATAGAGAATTCATTGGTGTGAACAGCACAAC
CGTTTCCTACTGGTGTAAACTCCGTGTCTGCAGATTTACAGCGTTGTTTTCAAAGGCTCCCTTCGGAAC
GTGTAACGGTTGGTTTGTGTCGCTGTGTCACACTTACACGCAAGGAAGCTGAGTTTTGCCTCTGTAGA
TCTGTTGGTTTTAAACCGTCTAAAAGACTAAAGCTAAAATGCTCCTCTTCAGAGCTGAGGTCCAAGCCA
GTGTTTAATCTTTGCATCCTTTTGAGTGATCCTTCTAAGACTAAGTGGAAGCATGTGGACTTTGAGTCTT
TTCAACCGAGCGCTCTCTCAAATCAGACACCCCCTAATGTGCAGACACGGGAATGAGCAGGCAAATG
GTTGGCCTGTCTTTGGGAAAACGGATTCACTTTTTTAAATAACCAATTCCTCTGCATATGGGGGGGAATA
AAGCAGTTTAAAGTTGTATATTGCCACTTCAAATTGAGGGGAAAAATTTAATGTATTTGGAGTTGGTCTC
AGATCTCTAAAAGAGTCAAAGGTCACAGCTTCCCCCAACATTGTCCCAGCCATTTCTCATATGTATATAG
TATAAACCGTGACAAACACTGCCTTTATATTATTTAGCAATATGTTGTAAATAGCATTATGAAGCTCTTTTT
TTGTAATAAAGACCCTATGATTTGAATATAGTACAATAACTGAGCTGATAAAGTCAATTTTTGAATTTTTTT
TTAGCTAGAGGCAATTTCAATTGTGCATTTTTGTTATTGTCTATTGTTCTGAAGACTGCATAATTTATTGGT
TTAATTTATCCTAATTTATTTGATGAAGGTGTACAATTTTGTATTACCAAGGATGTACTGTAATATTAATTGA
TAGGATTAAAAACAGTGAGACTCCCTGTCCATATTCAAAAGAAAACAAAAAGGTGCCGTAGACAATTGA
TTTTAAAGTAAAAAGTAAAAACAATTTAGTTTGGCAGCTACTAAATTTTAAAACAAGGAAAAAAAAAAAAA
ACCTAAAGGACAATGTTGTCGTGGGGGGGCGGGGAGGGTTTCTGTTGTGTGTTTTAAGCTTTTCTATAT
TCTCCGAACTTTGACCGTTTGCTTTGTACCACTAAAGGGTGCAGTAGTCCAACTGCCTTGTGTGCCTT
CCATCTTCTCCTAAACTGAATGTATGTGCAGTATATATGCAAGCTTGTGCAAAATAAAATATACATTACAAG
CTCATGCCATTCTTTTGTTTTTTTTGTTTTTTTTGTTTTTCTGAAAACCCGTGGCATTTTGCTCTTGGACC
TATTAGGCTTTGATCTATAGGCCTGGAGATGTCTTTTCTGTGTCTGGAGGCTTGCTTTCCGTGTAGAGT
CTCTCATTCTGATTAACTTTGGTTGGCCTTCAGCTTGAGGGGAGAGAAAGGTGGTTCCCGCTTGAGGA
GATGCATCCTGGGTAAAGCACTTGTGATGTGAGGGTTGGGACCTGGACCGACATAAAGACAGGTTTAG
GAGCATGCATCTTCAGTCCCAGAACTTTTACAATAAGACAGGA

Supplemental Table 3

Primer sequences used for genotyping.

Allele	Size (bp)	Forward primer	Reverse primer
WT LPA4	288	CCCCTACACCTTAAACATGATTGGC	GTACTTTCTTCCCAGCACAACCTCA
Floxed LPA4	350	CCCCTACACCTTAAACATGATTGGC	GTACTTTCTTCCCAGCACAACCTCA
KO LPA4	715	GTGAAAACACATTACTGGCTATG	GTACTTTCTTCCCAGCACAACCTCA
WT LPA6	303	GTGACCACATCTGAATAGCAAAGG	AAAAATCCGAAATGGCAAAGTAAA
Floxed LPA6	517	GTGACCACATCTGAATAGCAAAGG	AAAAATCCGAAATGGCAAAGTAAA
KO LPA6	706	GTGACCACATCTGAATAGCAAAGG	TTGCAGCCAGAATATAAATC
Prox1-Cre	614	CAAATCCCCTAACTGCCTACAAGA	CAATCCCCAGAAATGCCAGA
Prox1-CreER^{T2}	614	CAAATCCCCTAACTGCCTACAAGA	CAATCCCCAGAAATGCCAGA
Internal Control	302	CAAATCCCCTAACTGCCTACAAGA	TCTCCTGAAAACCAACCCTACC
CAG-tdTomato	200	GGCATTAAAGCAGCGTATCC	CTGTTCCTGTACGGCATGG

Supplemental Table 4

Primary antibody information for immunohistochemistry, immunocytochemistry, and western blotting.

Name	Source	# Catalog	Dilution
Goat Anti-Prox1	R&D Systems	AF2727	IHC for LVV (1;1000)
Goat Anti-VEGFR3	R&D Systems	AF743	IHC (1;1000)<br>IHC for section (1;200)
Mouse Anti-FOXC2	Santa Cruz Biotechnology	sc-515234	ICC (1;100)
Mouse Anti-Gα12	Santa Cruz Biotechnology	sc-409	WB (1;1000)
Mouse Anti-Gα13	Santa Cruz Biotechnology	sc-410	WB (1;1000)
Mouse Anti-α-Smooth Muscle Actin	Sigma-Aldrich	A5228	IHC (1;200)
Rabbit Anti-Histone-H3	Proteintech	17168-1-AP	WB (1;1000)
Rabbit Anti-IκBα	Cell Signaling Technology	4814	WB (1;1000)
Rabbit Anti-LYVE-1	AngioBio	11-034	IHC (1;100)
Rabbit Anti-LYVE-1	ReliaTech	103-PA50AG	IHC for LVV (1;1000)
Rabbit Anti-Phospho-IκBα	Cell Signaling Technology	2859	WB (1;1000)
Rabbit Anti-Phospho-RelA	Cell Signaling Technology	3033	WB (1;1000)
Rabbit Anti-Prox1	Proteintech	11067-2-AP	IHC (1;200)<br>ICC (1;200)
Rabbit Anti-RelA	Proteintech	10745-1-AP	ICC (1;200)
Rabbit Anti-RelA	Cell Signaling Technology	8242	WB (1;1000)
Rabbit Anti-β-Actin	Cell Signaling Technology	4967	WB (1;1000)
Rat Anti-CD31	BD Pharmingen	553370	IHC (1;100)<br>IHC for LVV (1;1000)
Sheep Anti-FOXC2	R&D Systems	AF6989	IHC (1;100)<br>WB (1;1000)

Supplemental Table 5

Secondary antibody information for immunohistochemistry, immunocytochemistry, and western blotting.

Name	Source	# Catalog	Dilution
Alexa Fluor 488-conjugated Donkey Anti-Goat IgG	Jackson ImmunoResearch	705-546-147	IHC (1;100)<br>ICC (1;200)
Alexa Fluor 488-conjugated Donkey Anti-Rabbit IgG	Jackson ImmunoResearch	711-546-152	IHC (1;100)<br>ICC (1;200)
Alexa Fluor 488-conjugated Donkey Anti-Rat IgG	Jackson ImmunoResearch	712-545-153	IHC for LVV (1;1000)
Alexa Fluor 594-conjugated Donkey Anti-Mouse IgG	Jackson ImmunoResearch	715-586-150	IHC (1;100)<br>ICC (1;200)
Alexa Fluor 594-conjugated Donkey Anti-Sheep IgG	Jackson ImmunoResearch	713-586-147	IHC (1;100)
Cy3-conjugated Donkey Anti-Rabbit IgG	Jackson ImmunoResearch	711-165-152	IHC for LVV (1;1000)
Cy5-conjugated Donkey Anti-Goat IgG	Jackson ImmunoResearch	705-175-147	IHC for LVV (1;1000)
DyLight 405-conjugated Donkey Anti-Rat IgG	Jackson ImmunoResearch	712-476-150	IHC (1;100)
HRP-conjugated Donkey Anti-Sheep IgG	R&D Systems	HAF016	WB (1;1000)
HRP-conjugated Goat Anti-Rabbit IgG	Novus Biologicals	NB7187	WB (1;1000)
HRP-conjugated Rat Anti-Mouse IgG1	BD Pharmingen	559626	WB (1;1000)

Supplemental Table 6

Primer sequences used for qRT-PCR analysis for mouse genes.

Target gene	Forward primer	Reverse primer
Cdh5	CTCTGCATCCTCACCATCACAGT	GTGACCAACTGCTCGTGAATCTC
Cdk5	AACATCCTTGGTGAACGTCGTGC	ACAGAAGTCAGAGAAGTAGGGGT
Celsr1	GAAGACAAATGGAATCCGGCTGG	GTGTCCAACTCCTCACTGTCACT
Ctnnb1	GGATTACAAGAAGCGGCTTTCAG	GAAAAGAACGGTAGCTGGGATCA
Dchs1	TGTCGACCCTAACAATGGAACTC	GTCTCGGGCTTCCAGTACCACAT
Dll4	CCGGGAACCTTCTCACTCAAC	GCCAAATCTTACCCACAGCAA
Dot1l	TGAACAAGAAAGGGAGAAAGATGG	CTGGTAGAATGGGCTGTGAGGT
Enpp2	AATGGCTTACGTGACATTGAGGA	CAGGTCGGTGAGGAAGGATG
Ephb4	GCTGGATTGTTGGCAGAAGG	CCACAGAACCGAAAGCAGAATAG
Ephrinb2	GAAGCTGGTACAAATGGGAGAAGTT	GATGATGATGACGATGAAGATGATG
Fat4	TCGGAGTGAGAATGGCATTTTGA	GGATGATTCGCTCCACCTTCCCA
Foxc1	CAGCATACGGCACAACCTCTC	GCCCTTCTCCTCCTTGTCCTT
Foxc2	CAGGCCACCTCCTGGTATCT	TGACAGCTCGCATTGCTCAC
Foxo1	ATGGTGAAGAGCGTGCCCTACTT	TTCCAGTTCCTTCATTCTGCACT
Foxp2	GCCTGCACAAATGTTTTGTTCGA	CTCCATAGCCCAAACTTGTAGGT
Gapdh	AGCGAGACCCCACTAACATCAA	TCGTGGTTCACACCCATCAC
Gata2	TCAGACGACAACCACCACCTT	ATTCAGCCCCTTTCTTGCTCTT
Gja1	GCTTACTTCAATGGCTGCTCCTC	CTCGCTGGCTTGCTTGTTGT
Gja4	CGGGAGATAAAGGCACGAAG	GGATGAGAGCCCGTTGTAGGT
Gna12	AGTTCCGCGACACCATCTTC	CATCCCGTGCTTCTCGTTCT
Gna13	GCCCCACCATCTACAGCAAC	CGGGTATCAAATGCCATCAAC
Hdac3	GCTGAAGAGAGAGGTCCCGAGGA	TCTCCACATCACTTTCCTTGTCG
Itga9	TCCATCAACATCACAGCACCTC	CCGTCTCTCCAAACAACACAAAA
Lpa1	CGCCAGAGGACTATGAGGATGT	CAGCAGACAATAAAGGCACCAAG
Lpa2	CCGCTACCGAGAGACCACAC	ACTTACAGTCCAGGCCATCCA
Lpa3	TGCTCATTCTGCTGGTGTGG	TGATGAAGAAGGCCAGGAGGT
Lpa4	ACGGCTATTTTCATCACCAACCT	ATGGCTAGGAAACGATCCACAC

Lpa5	CTGGCTGTATATGGGTTGCTACG	GAAACCCTCGGCACTGAAGTAGT
Lpa6	GGTCATCTTCTGTTTCTGTTTTGTG	TGAGTTCTGAATTGTGTCTGAGGTG
Lyve1	GTGCTGGCTCTCCTCTTCTTTG	TCCTTCTGTTGATTCTTGGTTGTG
Mta1	ACAACGGCTCCCCTACCATC	AGACCCCTACTGGGCATCAA
Nfatc1	CTTCCGAGTTCACATCCCACA	CCAGACAGCACCATCTTCTTCC
Nos3	TGTCTGCGGCGATGTCACTATGG	GGTGCGCAATGTGAGTCCGAAAA
Notch1	CTGTGTGGATGAGGGAGATAAACA	AGGAAAAGCCGCCGAGATAG
Nrp1	CCACTGGAAAGAAGGACGTGTCT	TCTAGGTCTGTTGGTTTTGCACA
Nrp2	GGTTTCCAGAGAAGTATCCACACAA	TGCCAATCAGAGGTCCAACA
Pdpn	GTTTTGGGGAGCGTTTGGTT	GCACCTGTGGTTGTTATTTTGTCTT
Pecam1	CACAGATAAGCCCACCAGAGACA	TTCACAGAGCACCGAAGTACC
Piezo1	ACTTCCTCGAGTGGTGGGTCATC	GATGGAGACGTACAGCCCCACAA
Pkd1	CTGTTACACTGCGCCTCGAGTTC	TGCACCTCGGCCATGGAAAAGTA
Prox1	AAATGACTTTGAGGTTCCAGAGAGA	TCTTGTAGGCAGTTAGGGGATTTG
Rela	CTCAACTTCTGTCCCCAAGCCAG	GCCTGGTTTGAGATCTGCCCTGA
Sox18	AATCAGGGCGCTATGGCTTT	GTTCAGCTCCTTCCACGCTTT
Taz	CTCAGCAACATGGACGAGATGGA	GGCAGTCCAGGAAATCAGGGAAG
Tie1	CAGGGGAAGGAGGAGAAGGA	GGCACCGAGGGTAGAGACCA
Vangl2	TTCTGCATTACCCACGACATGAC	CTCGCTCACCAAGGTCCACTGTT
Vegfr2	AGGGTGAGGAAGGAGGATGG	CCGTAGGACAATGACAAGAAGGAG
Vegfr3	TTTTGGGAAGGTGGTGGAAG	TTGAGATGGTTGCCGATGTG
Yap1	TCCGAATGCAGTGTCTTCTCC	CCGCTGTCTGTGCTCTCATCT
Zmiz1	CCAGCCAGATGATCATGCCCAAT	CCATGACCCTGGTAGTTGTTTCC

Supplemental Table 7

Primer sequences used for qRT-PCR analysis for human genes.

Target gene	Forward primer	Reverse primer
F3	GGAACCCAAACCCGTCAATC	CTGCTTCACATCCTTCACAATCTC
FOXC2	GAGTCCCAGGTGAGTGGCAAT	ATTTCGTGCAGTCGTAGGAGTAGG
GAPDH	CAGGTGGTCTCCTCTGACTTCAA	ACCCTGTTGCTGTAGCCAAATTC
GNA12	AGTTCCGCGACACCATCTTC	AACATCCCATGCTTCTCATTTTC
GNA13	AGTTCCGCCCCACCATCTAC	ACCCTTGTTTCCACCATTCCTT
LPA1	GAATCGGGATACCATGATGAGTC	GCACACGTCTAGAAGTAACAAAACC
LPA2	CTGGTCAAGACTGTTGTCATCATCC	AGGACTCACAGCCTAAACCATCC
LPA3	TAGGGGCGTTTGTGGTATGCT	ATGGGGTTCACGACGGAGTT
LPA4	GCAAGCCTGCTACTCTGTCTCAA	TTGCAAATCTTTCCAAAAAGCAA
LPA5	CGTGTCCTGACTACCGACCTACC	CAGCGAGAGGGTGAAGAGCA
LPA6	TCATCTGCGTCCTCAAAGTCC	CCAATTCCGTGTTGTGAAGTAAAA
RELA	CACCGGATTGAGGAGAAACGTAA	ATAGTTGATGGTGCTCAGGGATG
VEGFR3	AGAGACTTTGAGCAGCCATTCATC	GTCATCCCACACCACCTCCT
