# Supplementary material for: Lysophosphatidic acid–mediated NF-κB activation promotes FOXC2 expression essential for lymphatic valve development
Source: J Clin Invest. 2026 Jan 13;136(5):e193364. doi: 10.1172/JCI193364 (PMC12948439; doi:10.1172/JCI193364)
Supplement: Unedited blot and gel images [file jci-136-193364-s309.pdf]

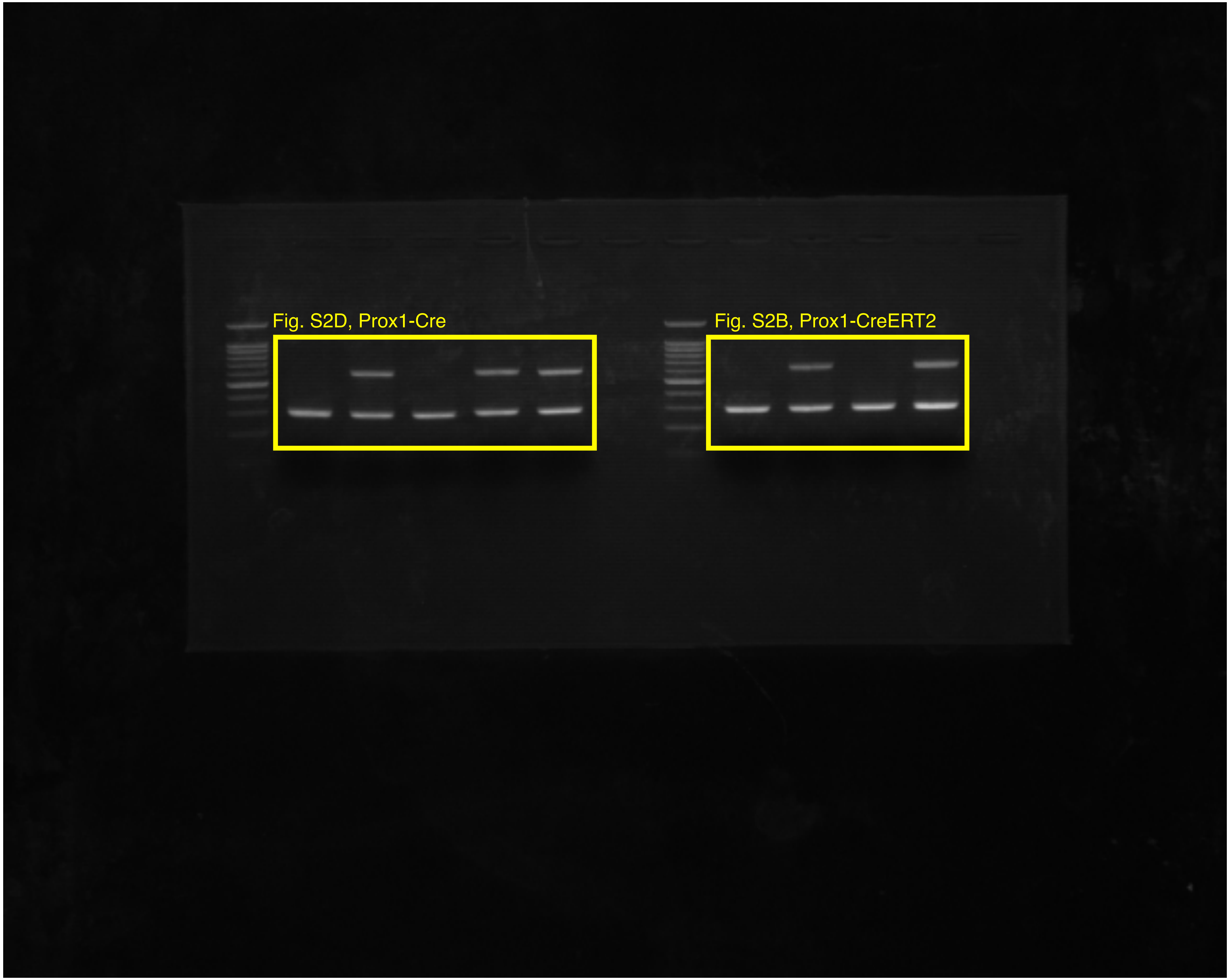
Fig. S2D, Prox1-Cre
Fig. S2B, Prox1-CreERT2

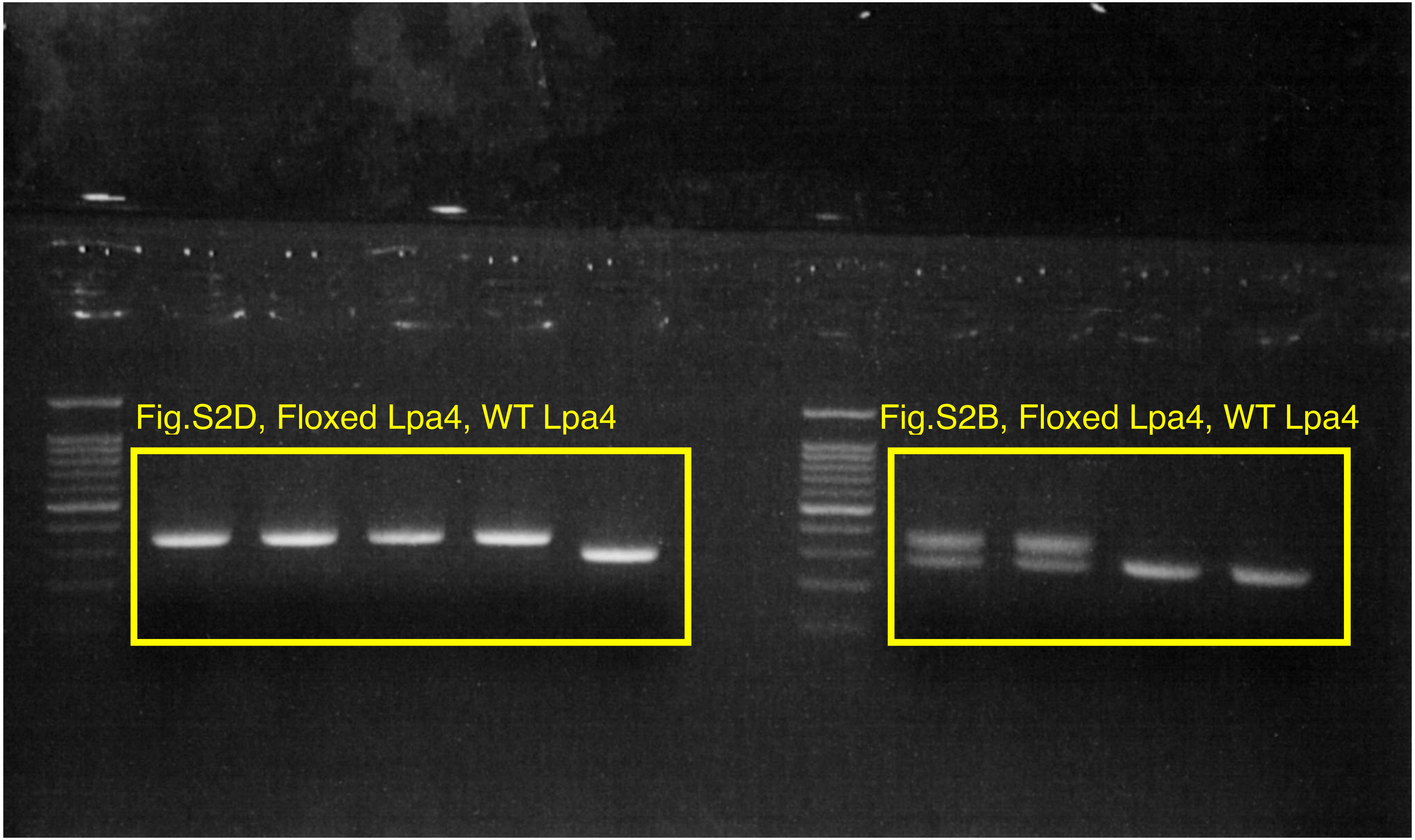
Fig.S2D, Floxed Lpa4, WT Lpa4
Fig.S2B, Floxed Lpa4, WT Lpa4

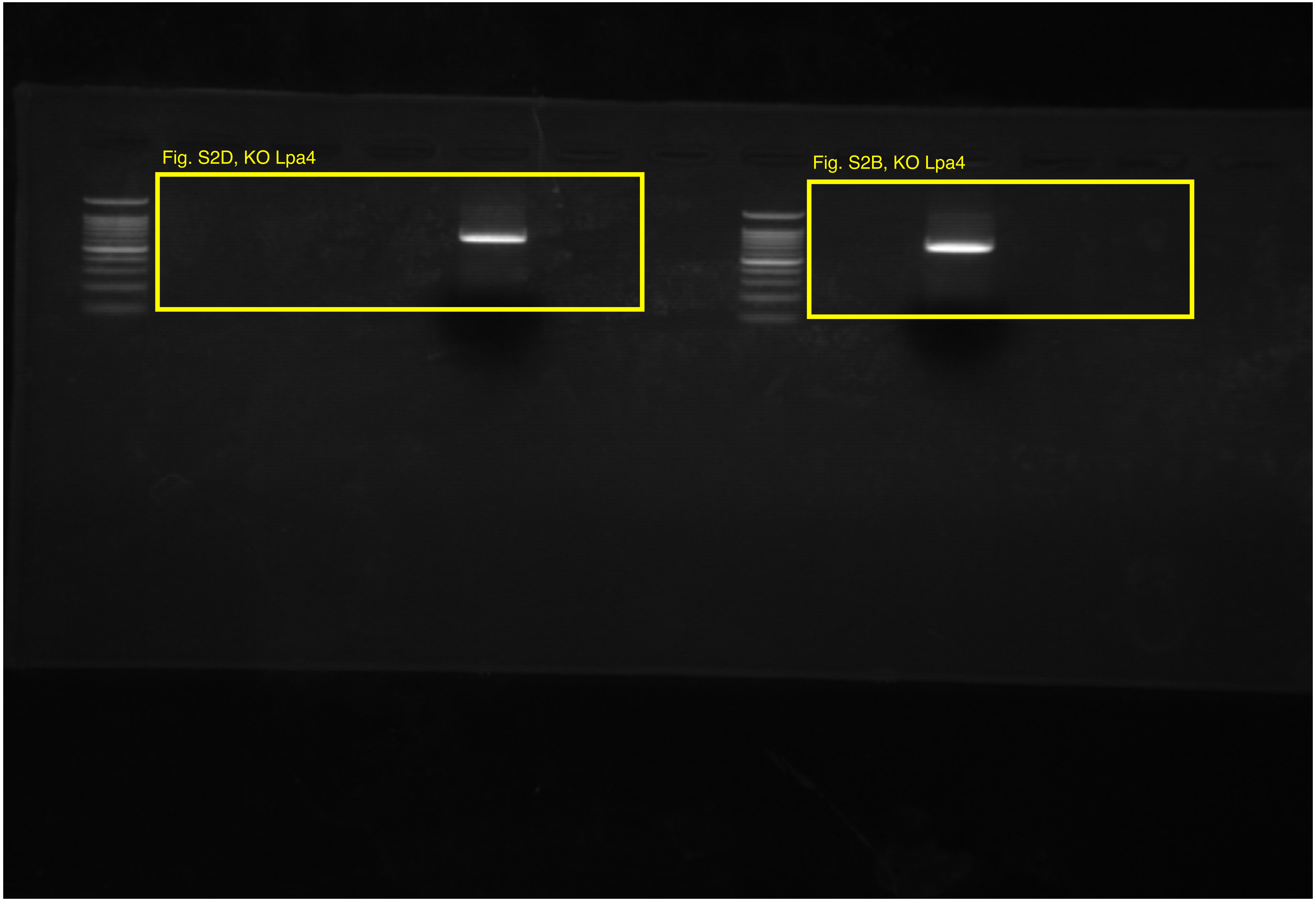
Fig. S2D, KO Lpa4
Fig. S2B, KO Lpa4

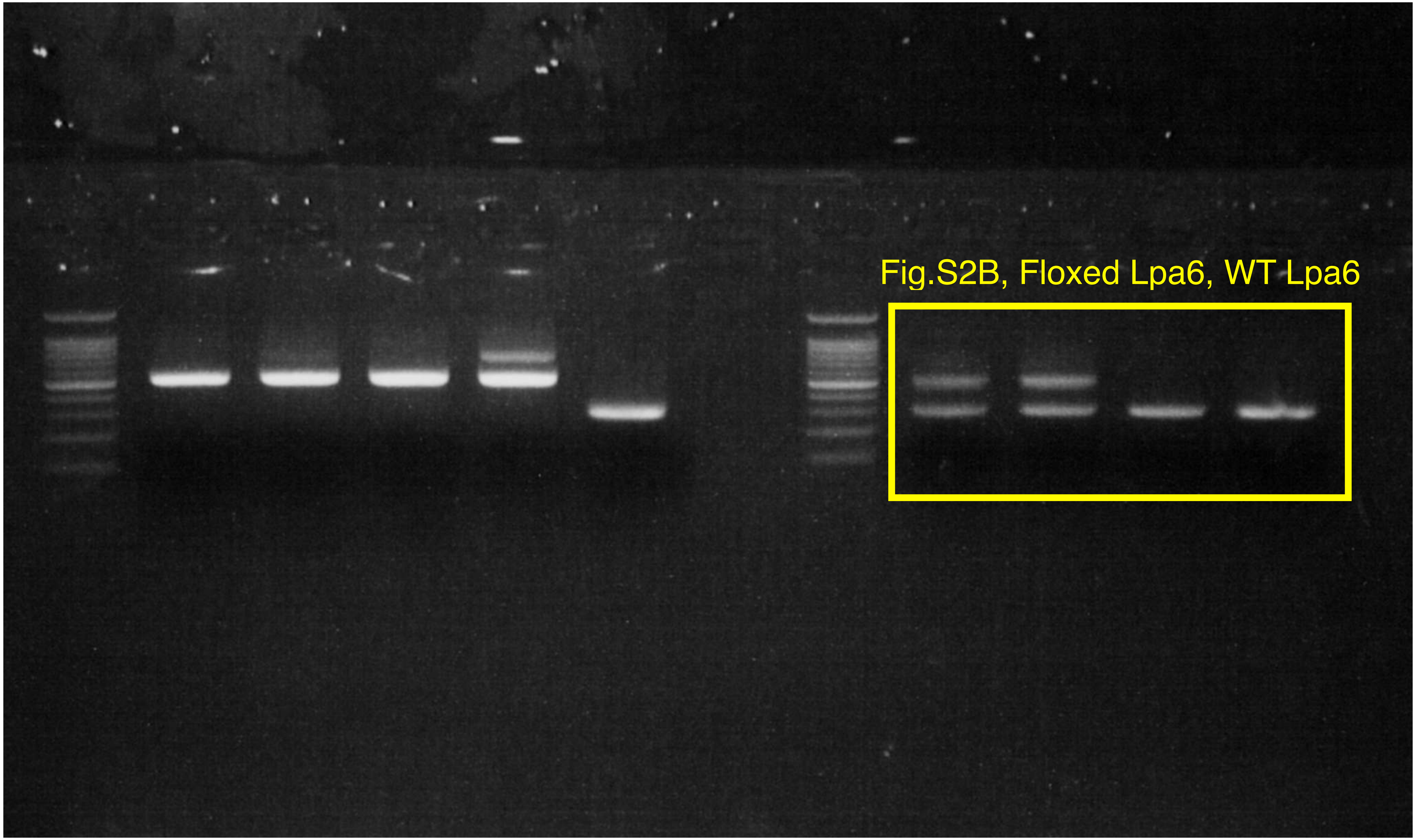
Fig.S2B, Floxed Lpa6, WT Lpa6

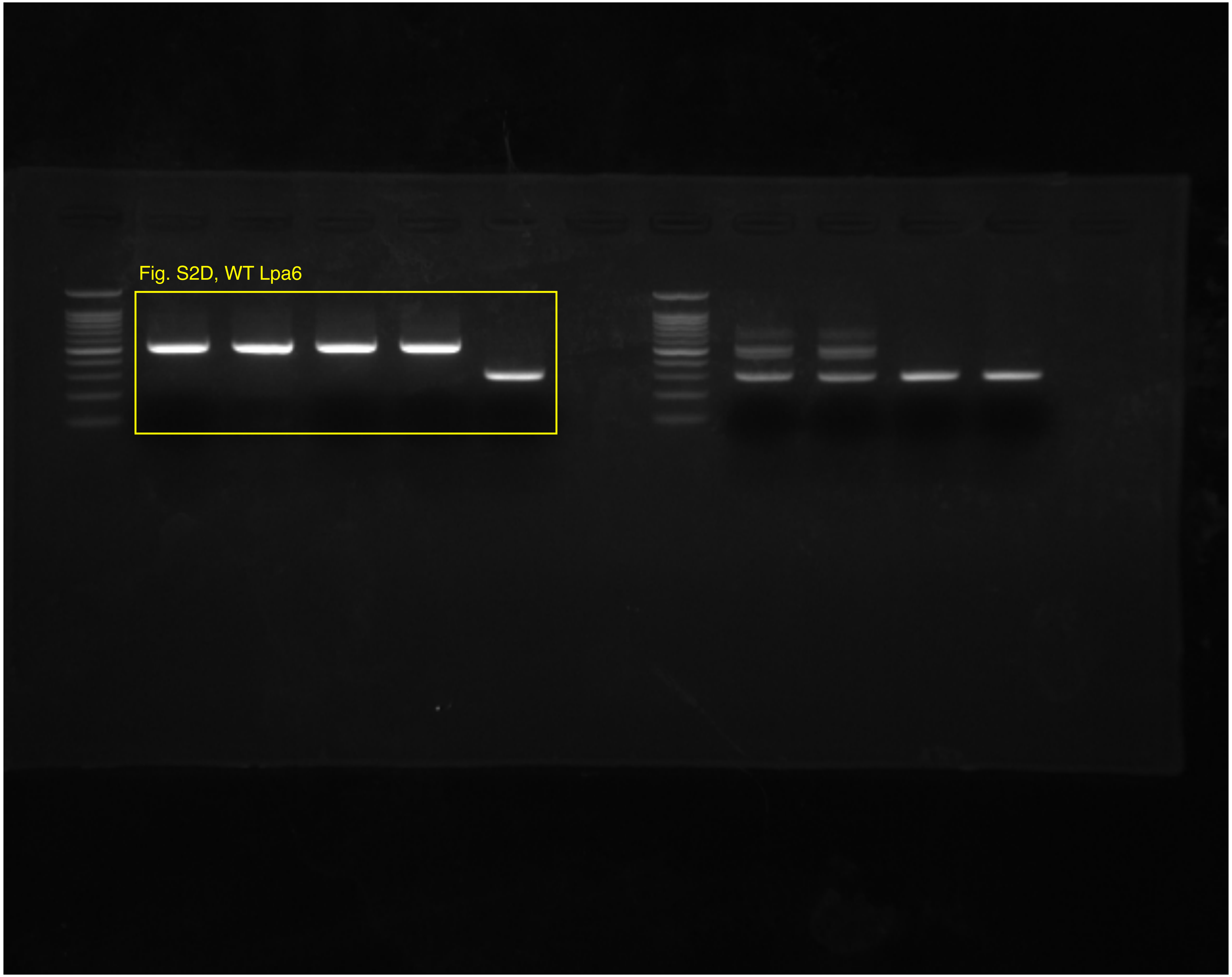
Fig. S2D, WT Lpa6

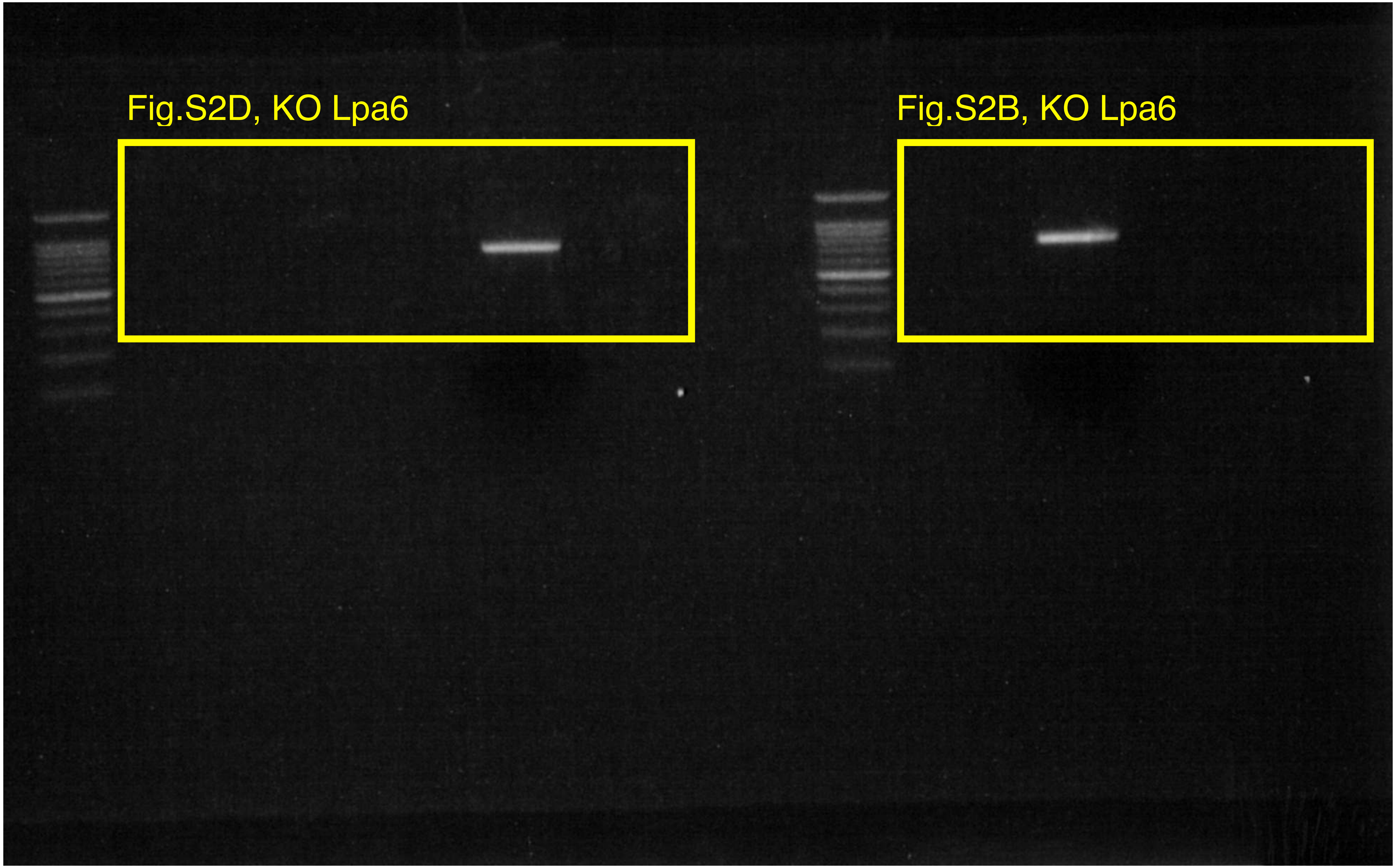
Fig.S2D, KO Lpa6
Fig.S2B, KO Lpa6

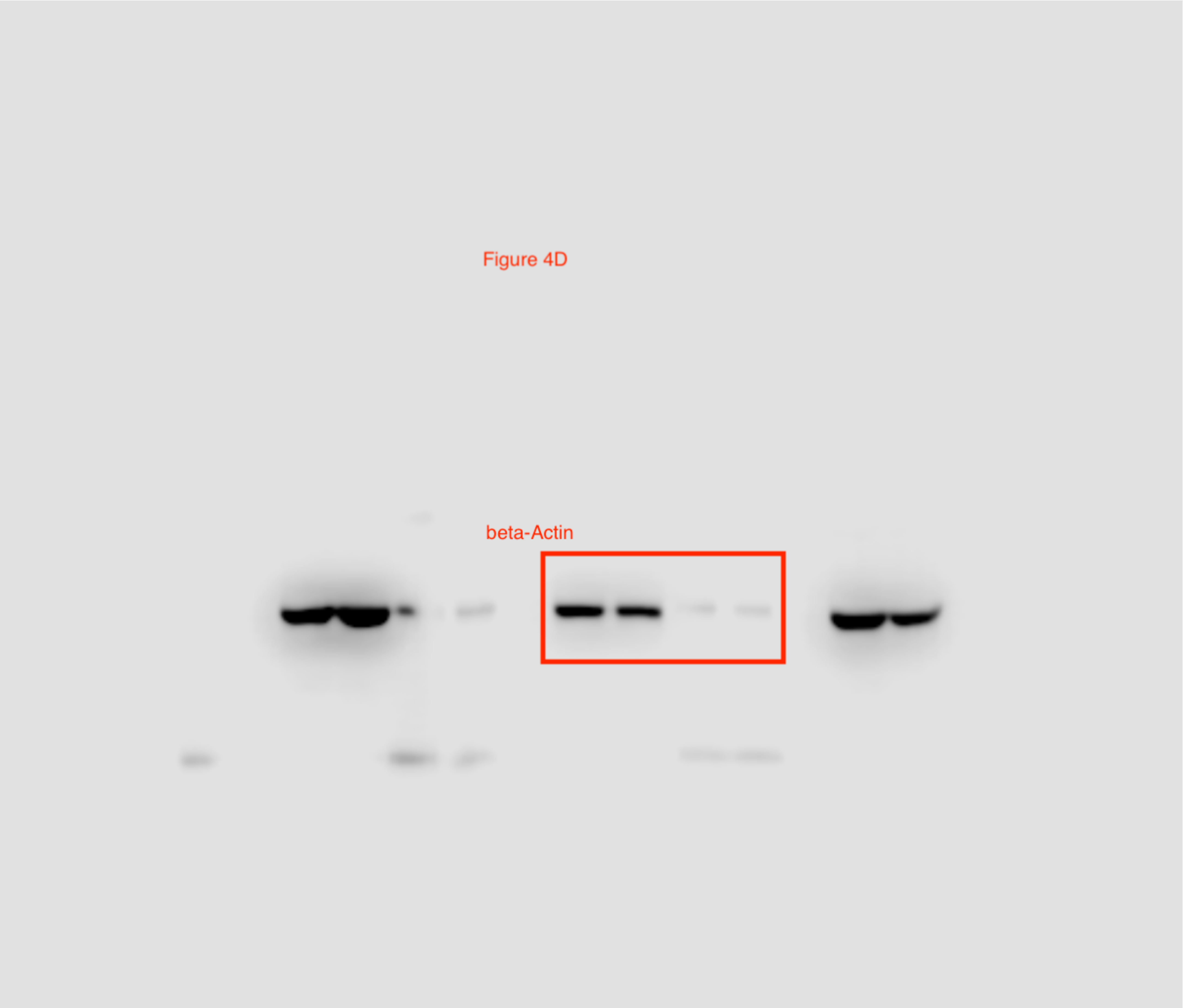
Figure 4D
beta-Actin

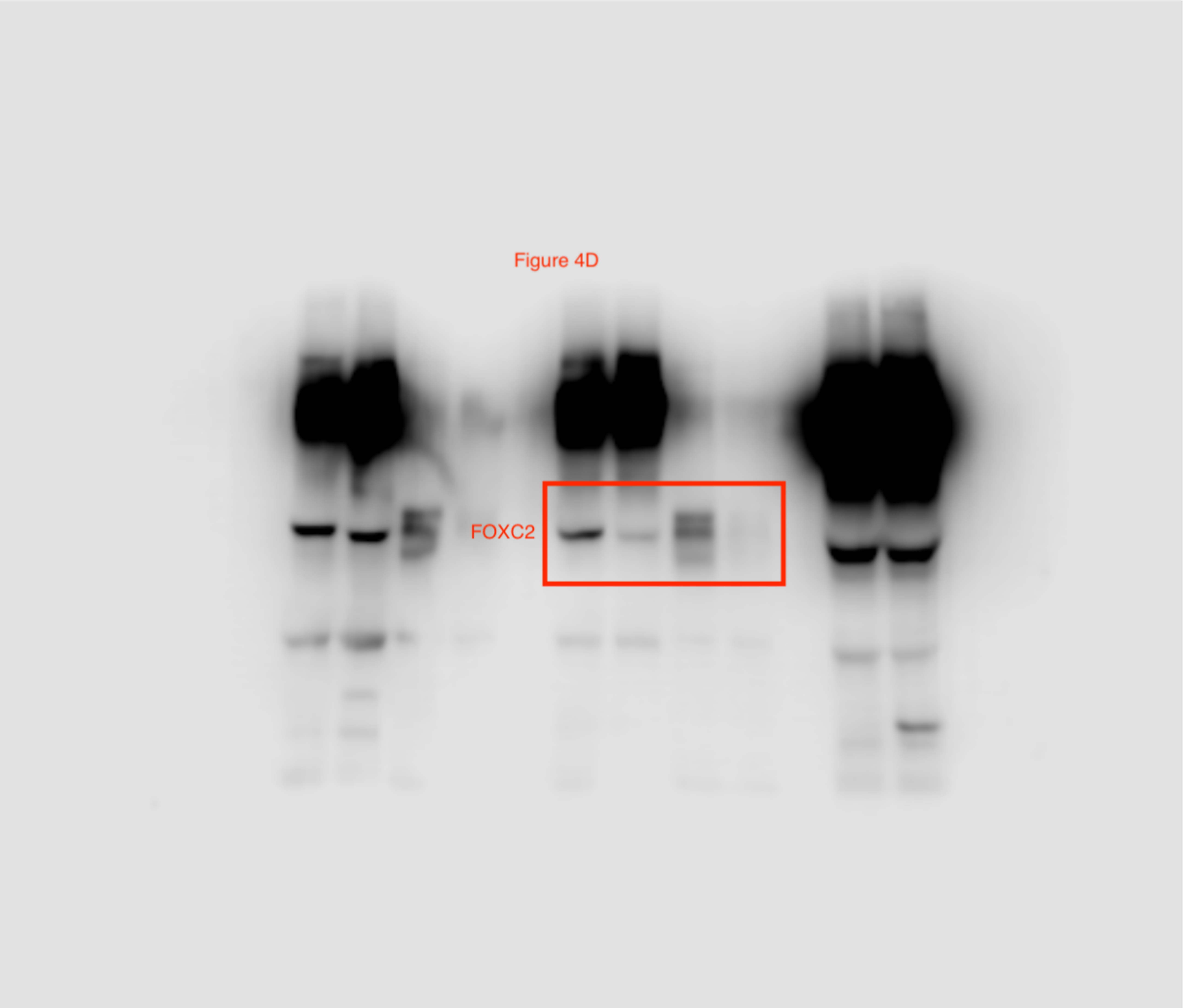

Figure 4D
FOXC2

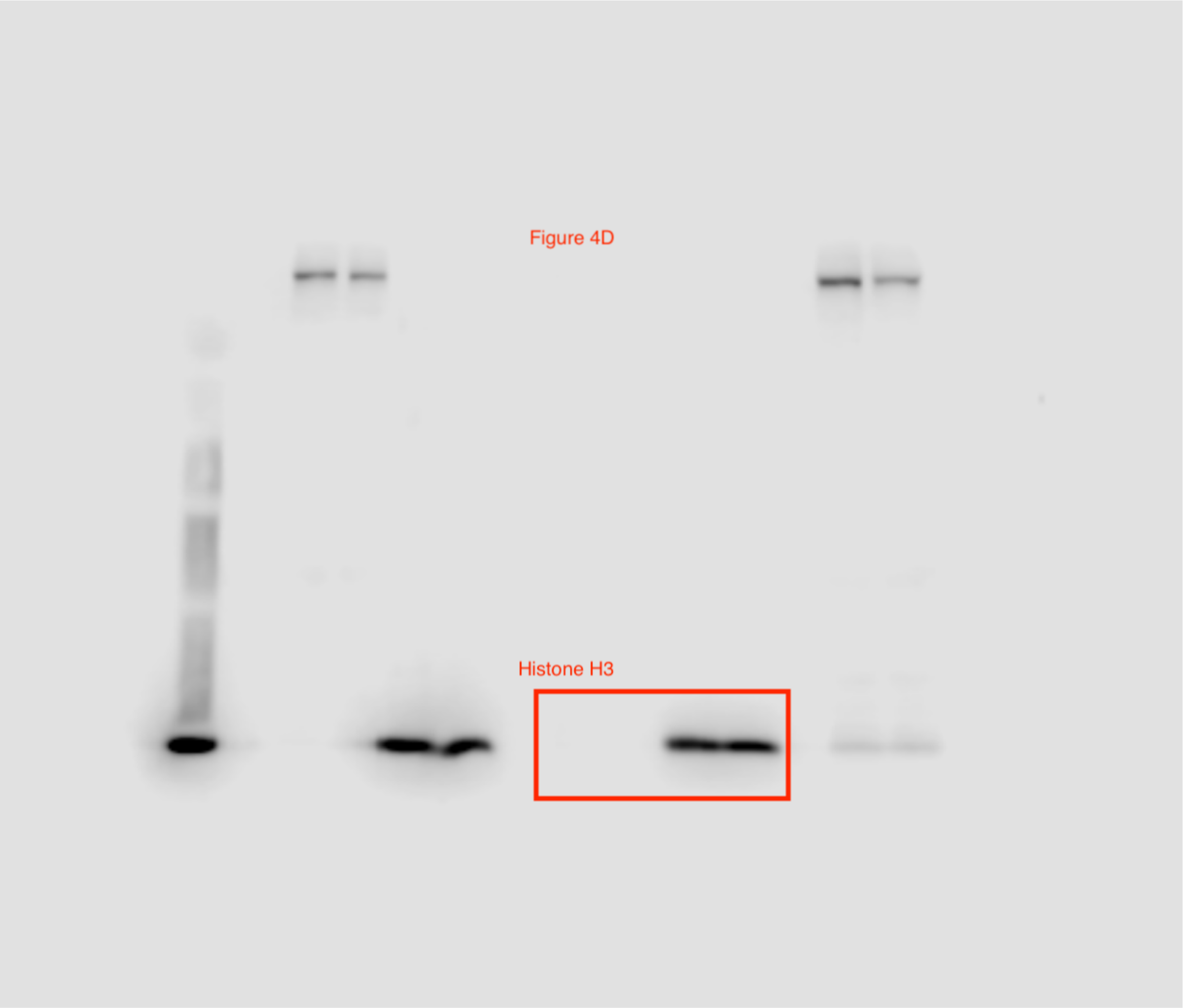
Figure 4D
Histone H3

Figure 6B

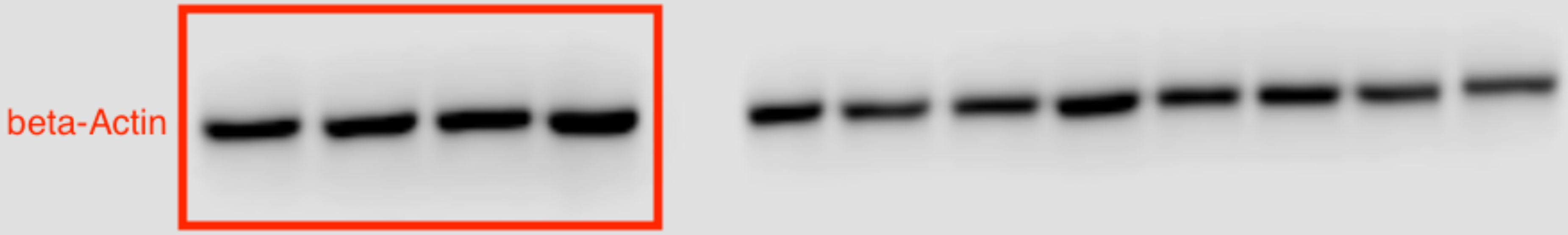

Figure 6B

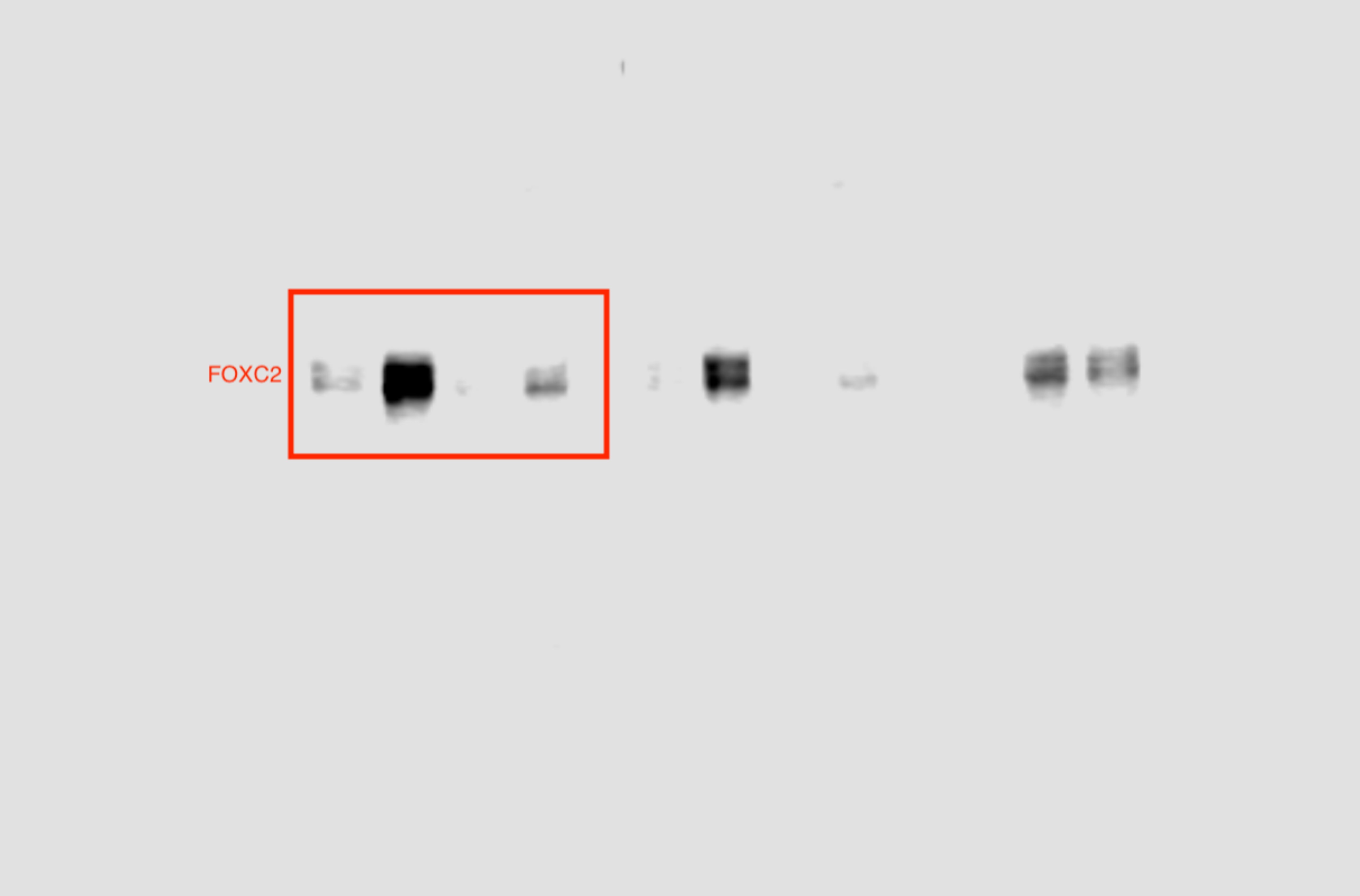

Figure 6C

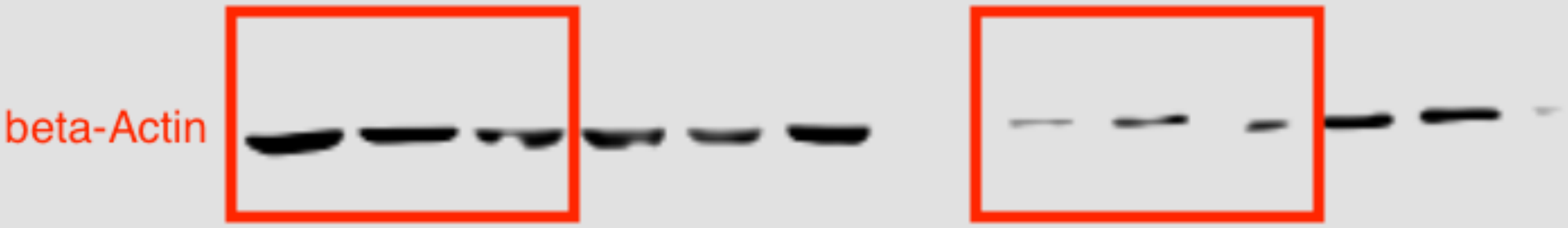

Figure 6C

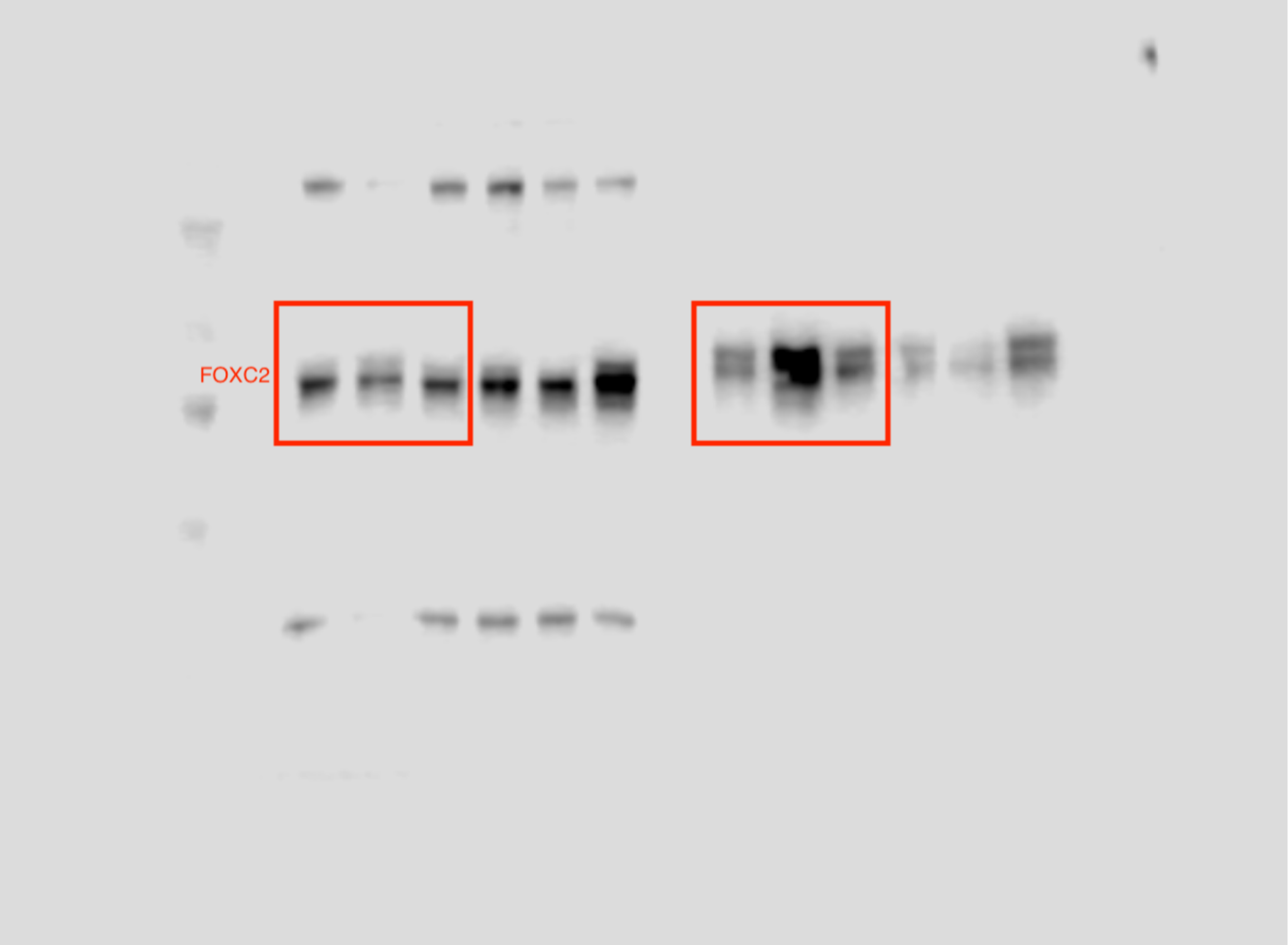

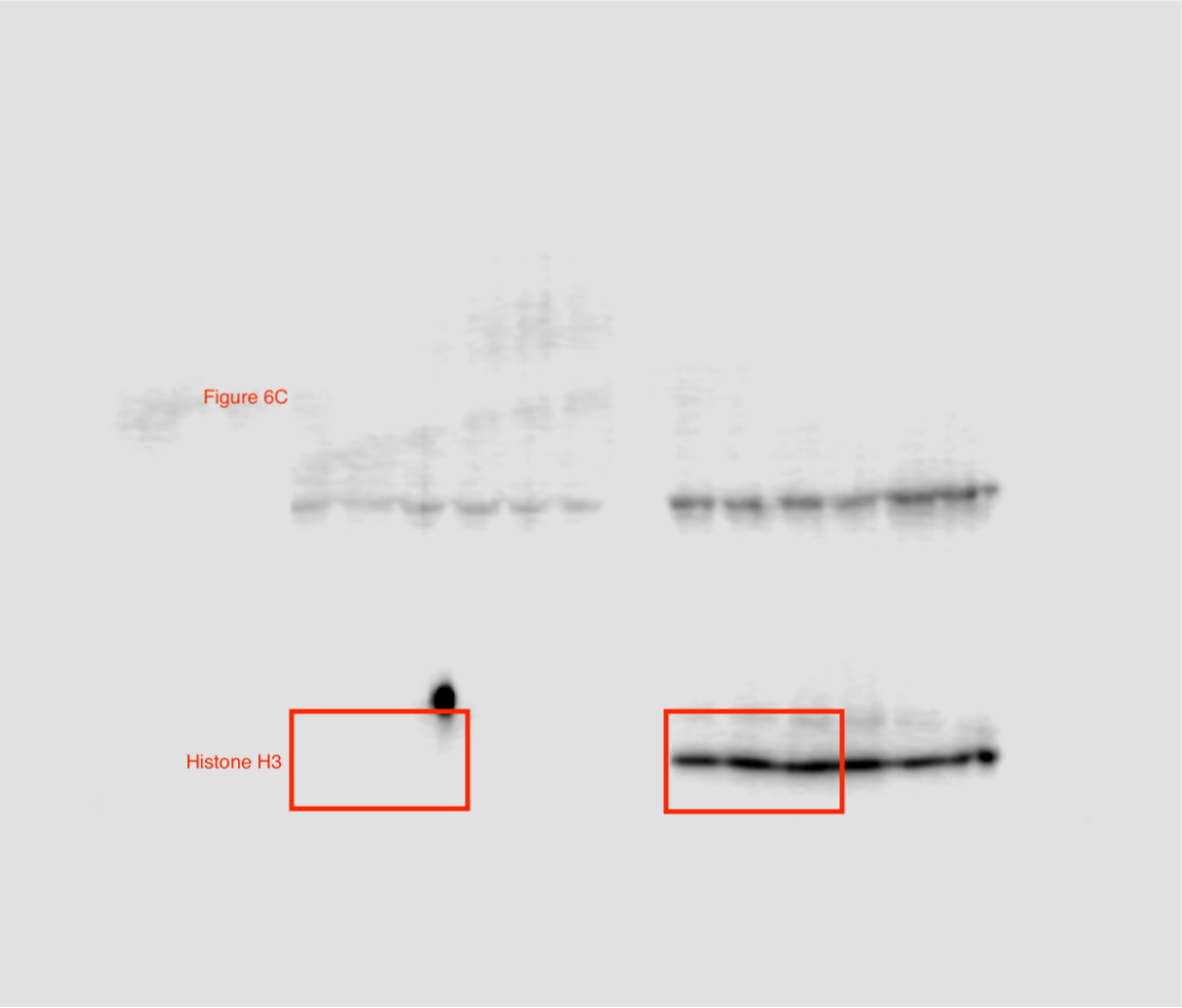
Figure 6C
Histone H3

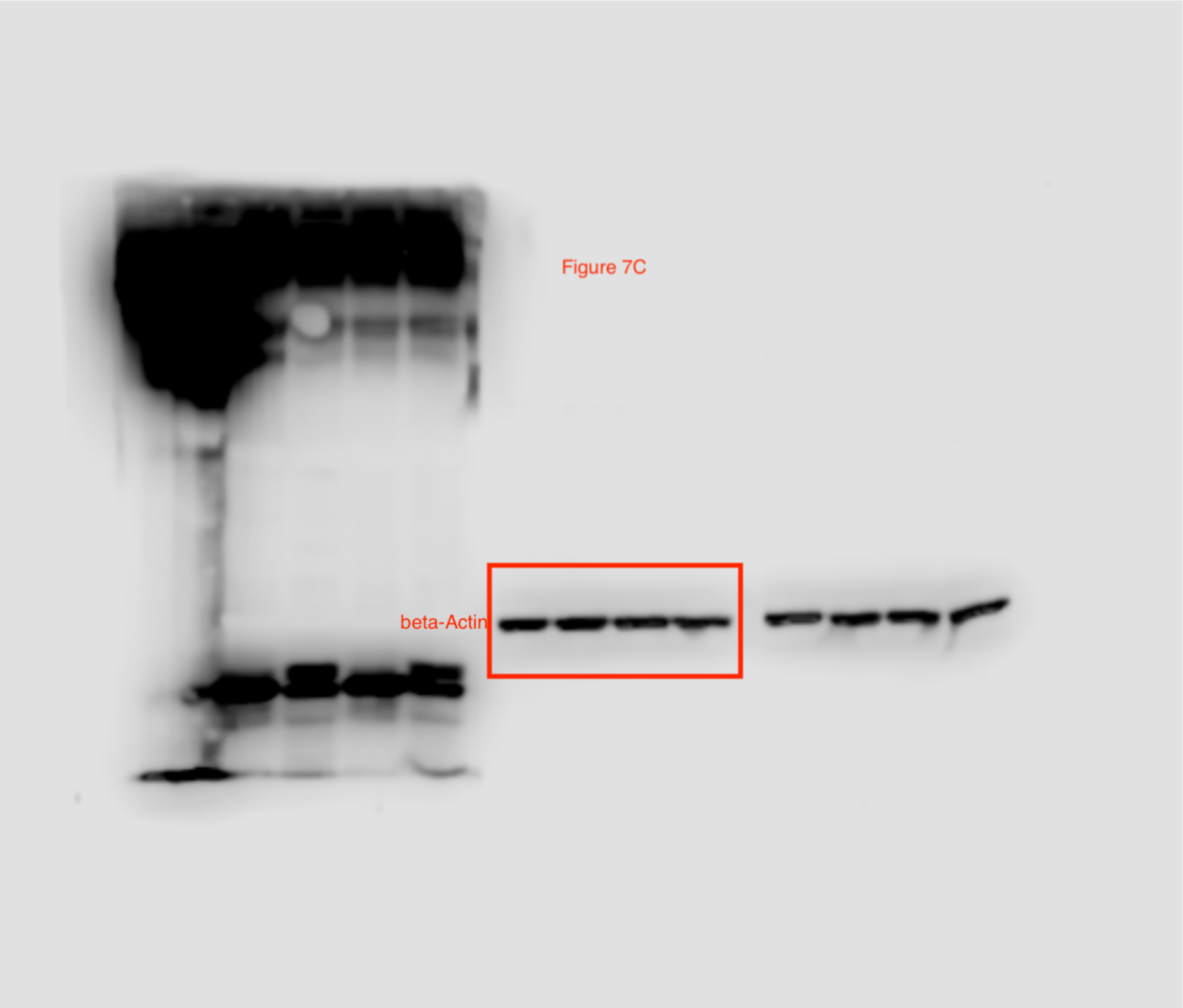
Figure 7C
beta-Actin

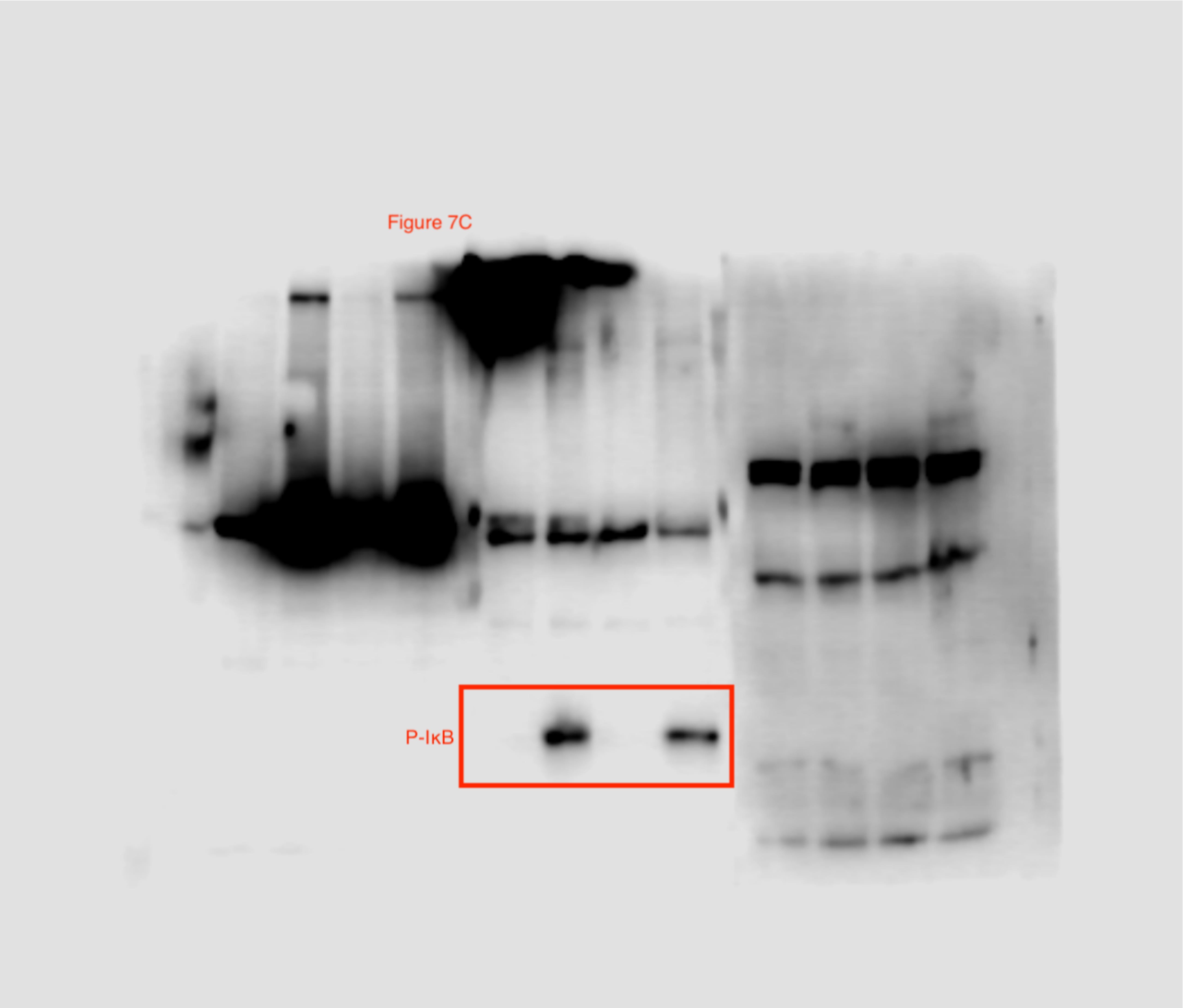
Figure 7C
P-IκB

Figure 7C

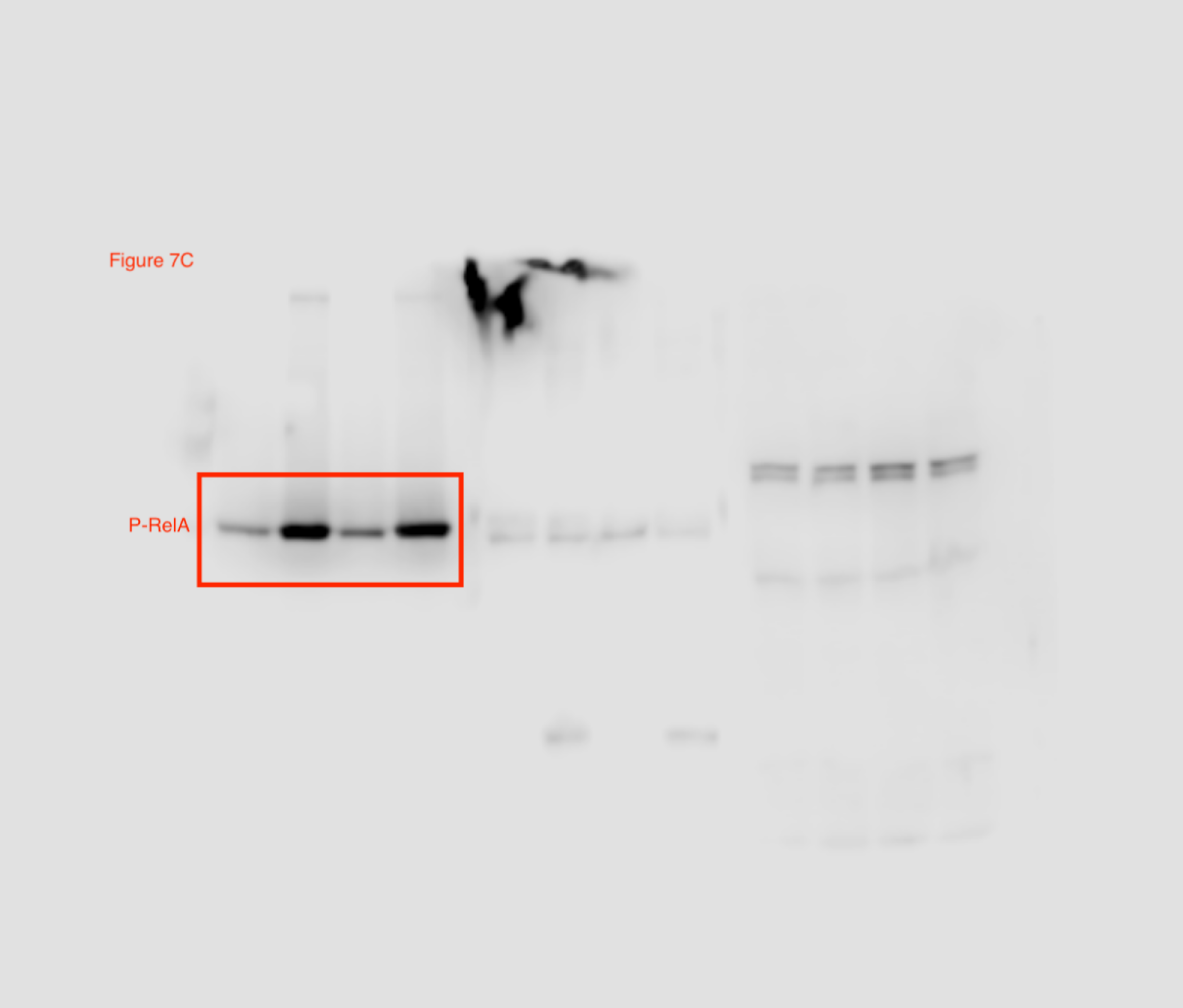

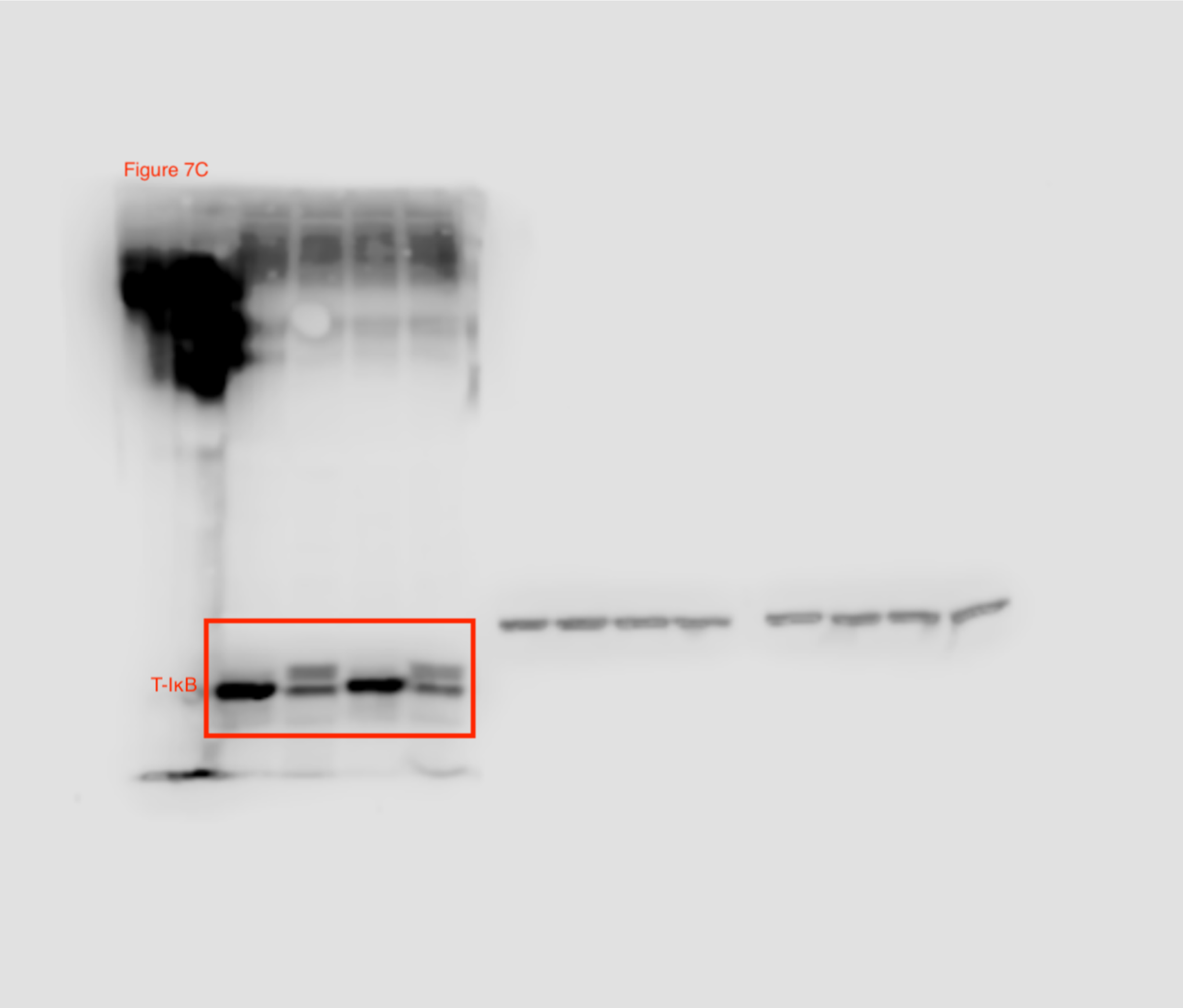
Figure 7C
T-IκB

Figure 7C

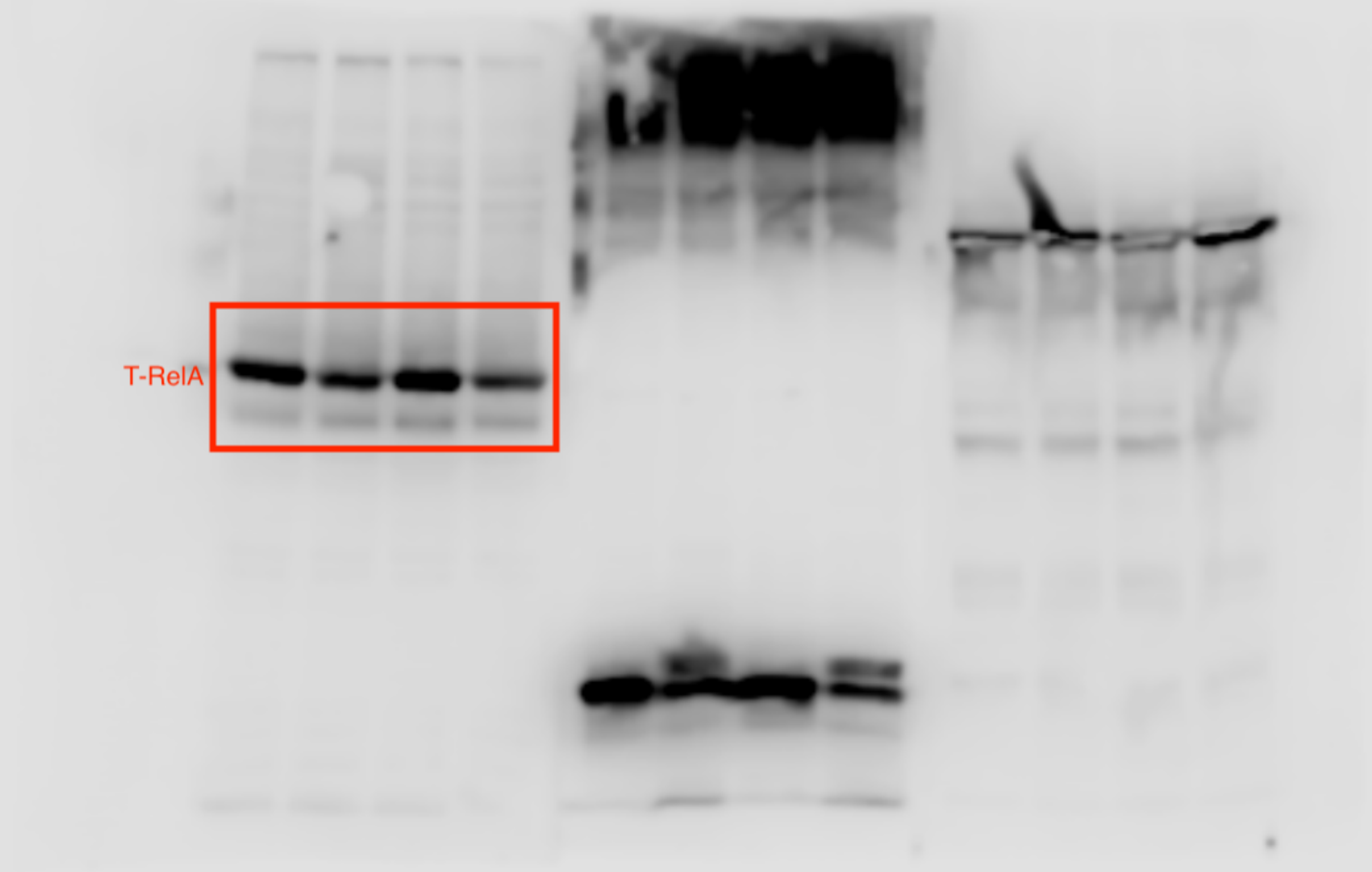

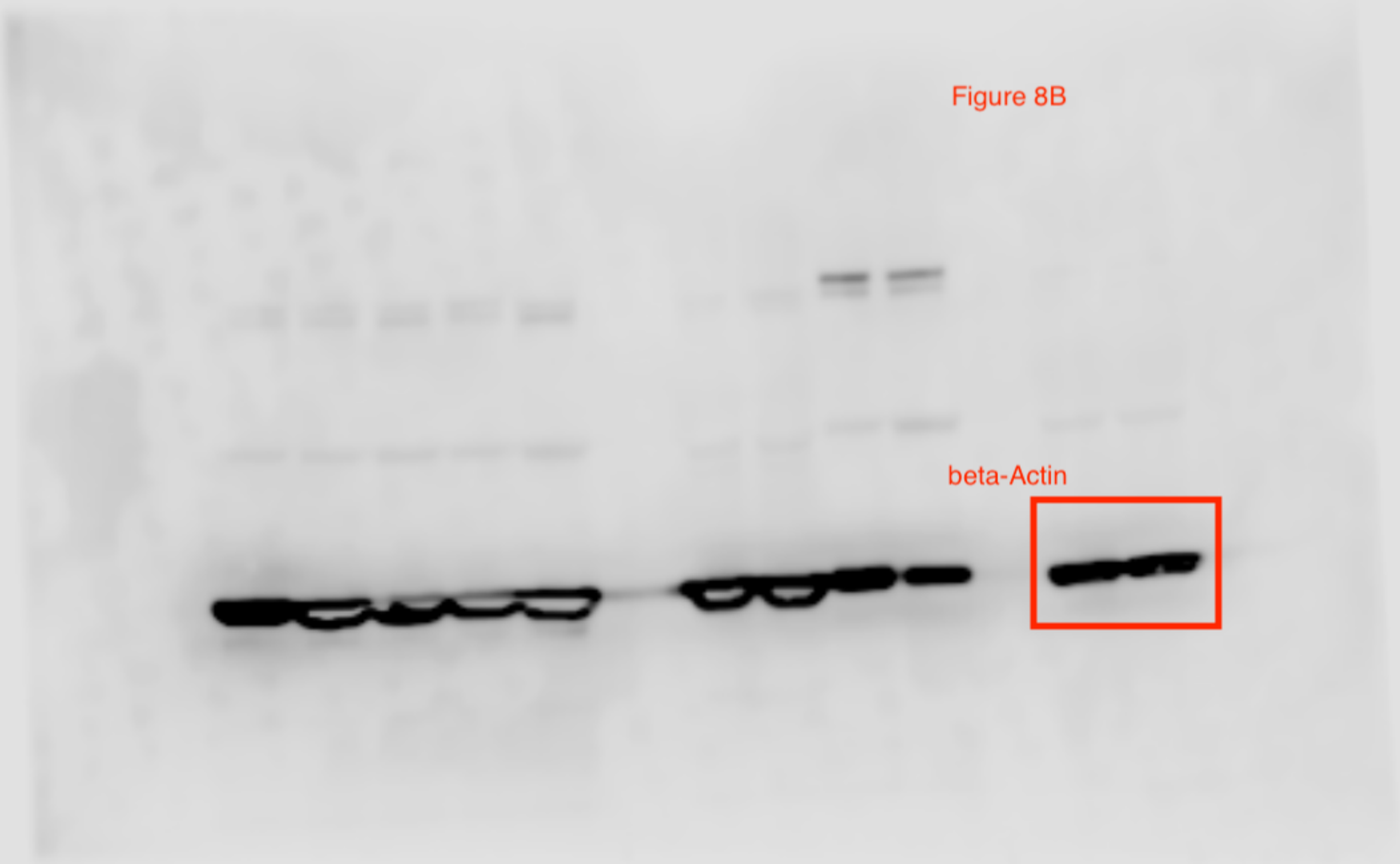
Figure 8B
beta-Actin

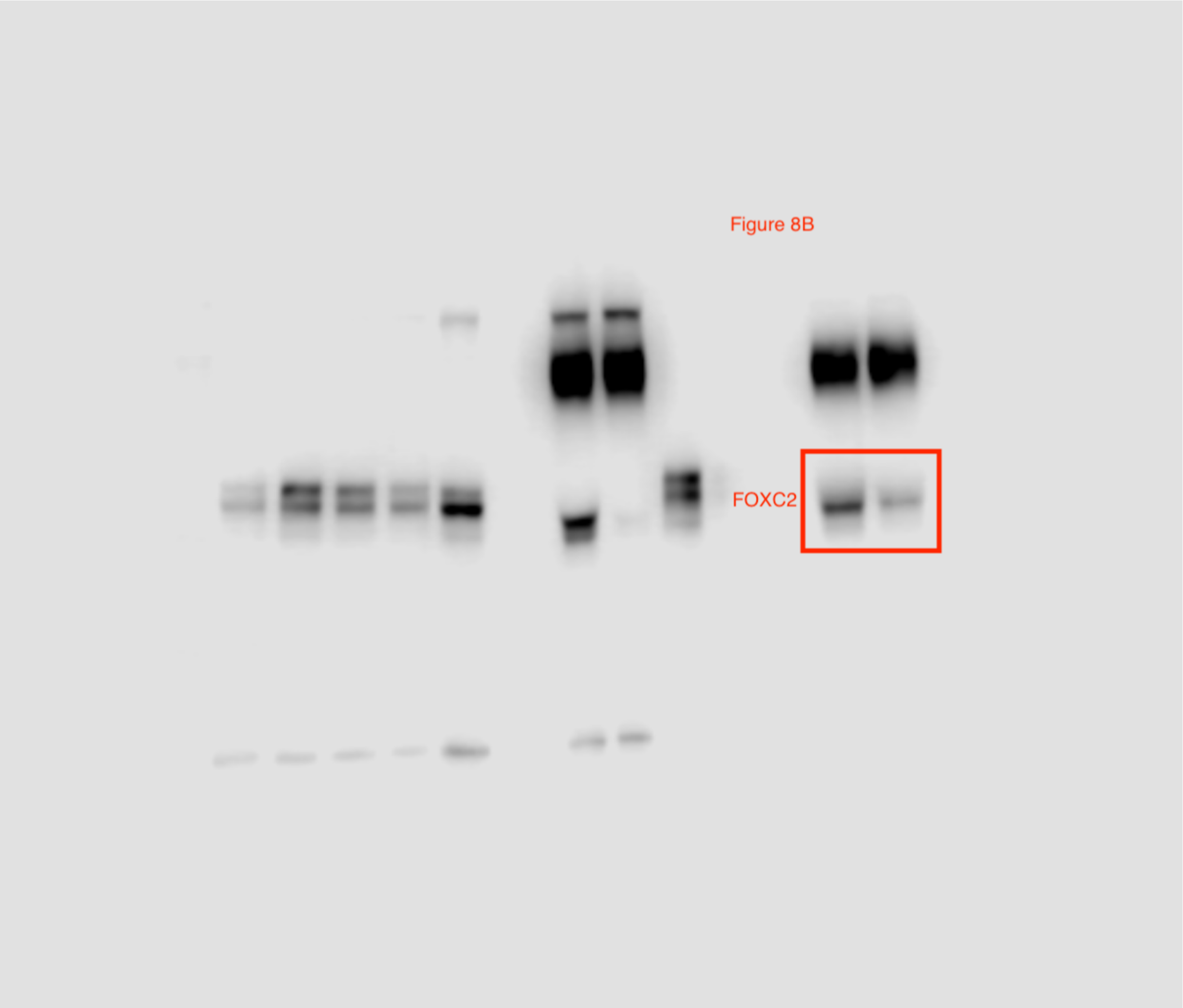
Figure 8B
FOXC2

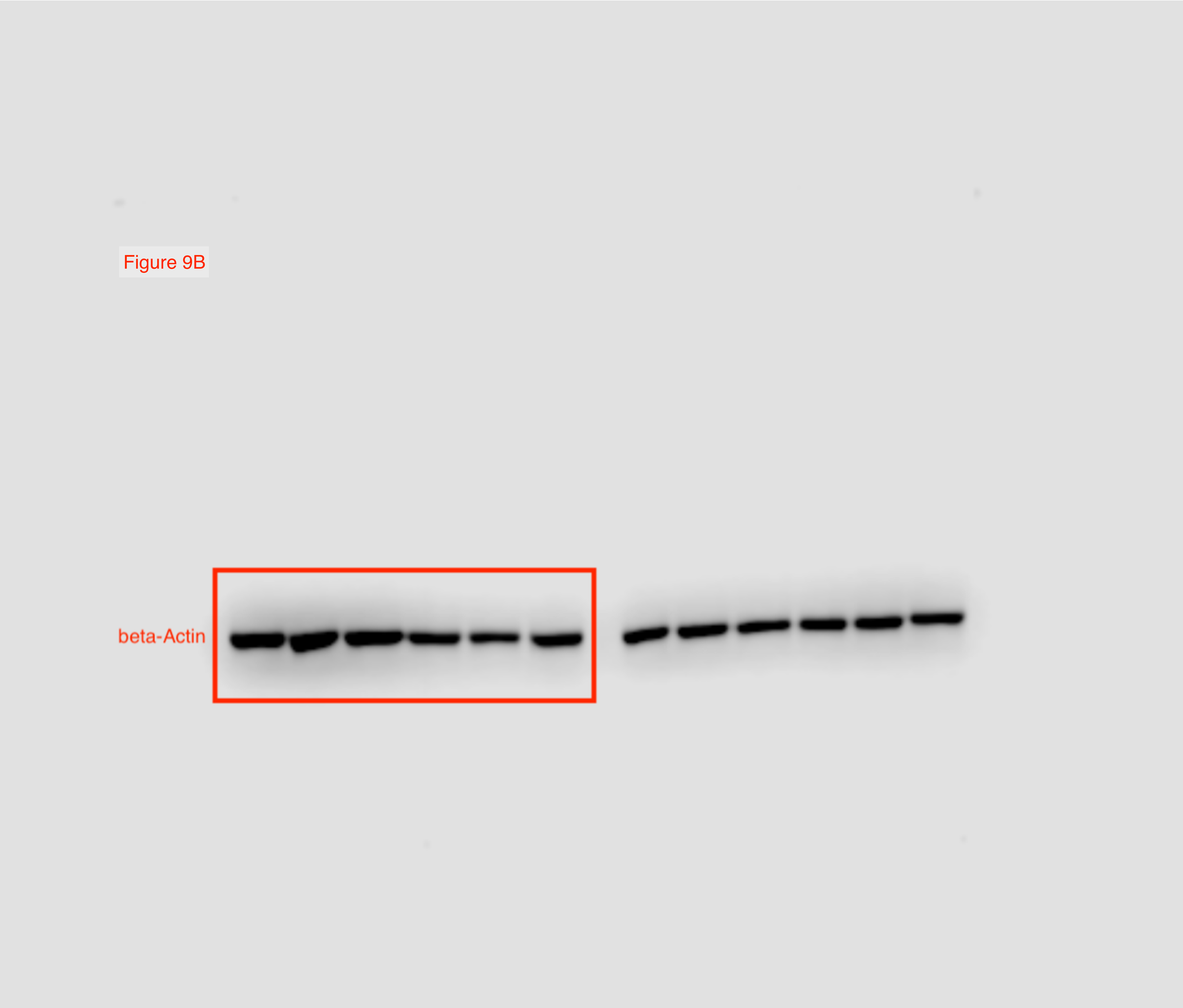
Figure 9B
beta-Actin

Figure 9B

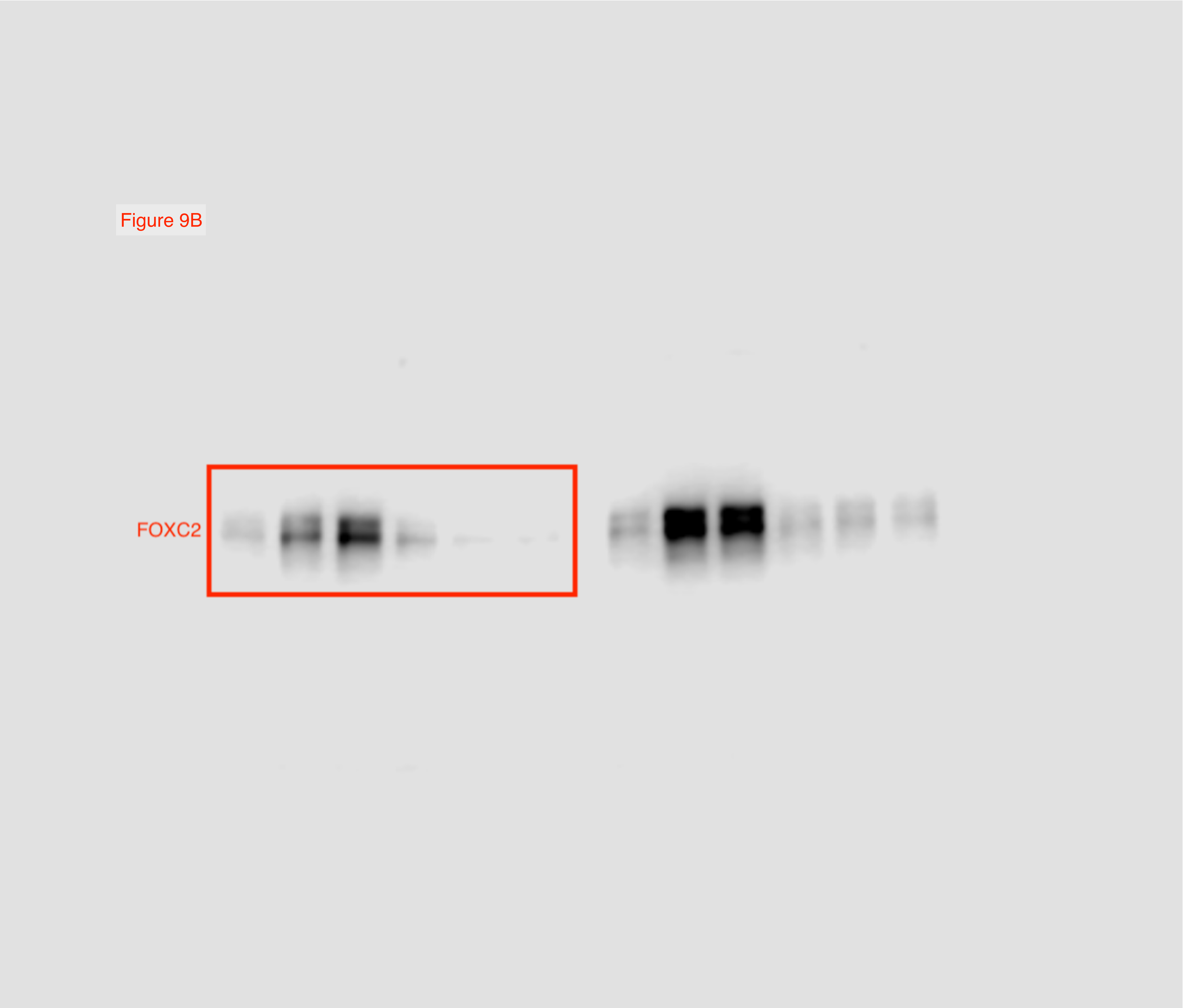

Figure 9E

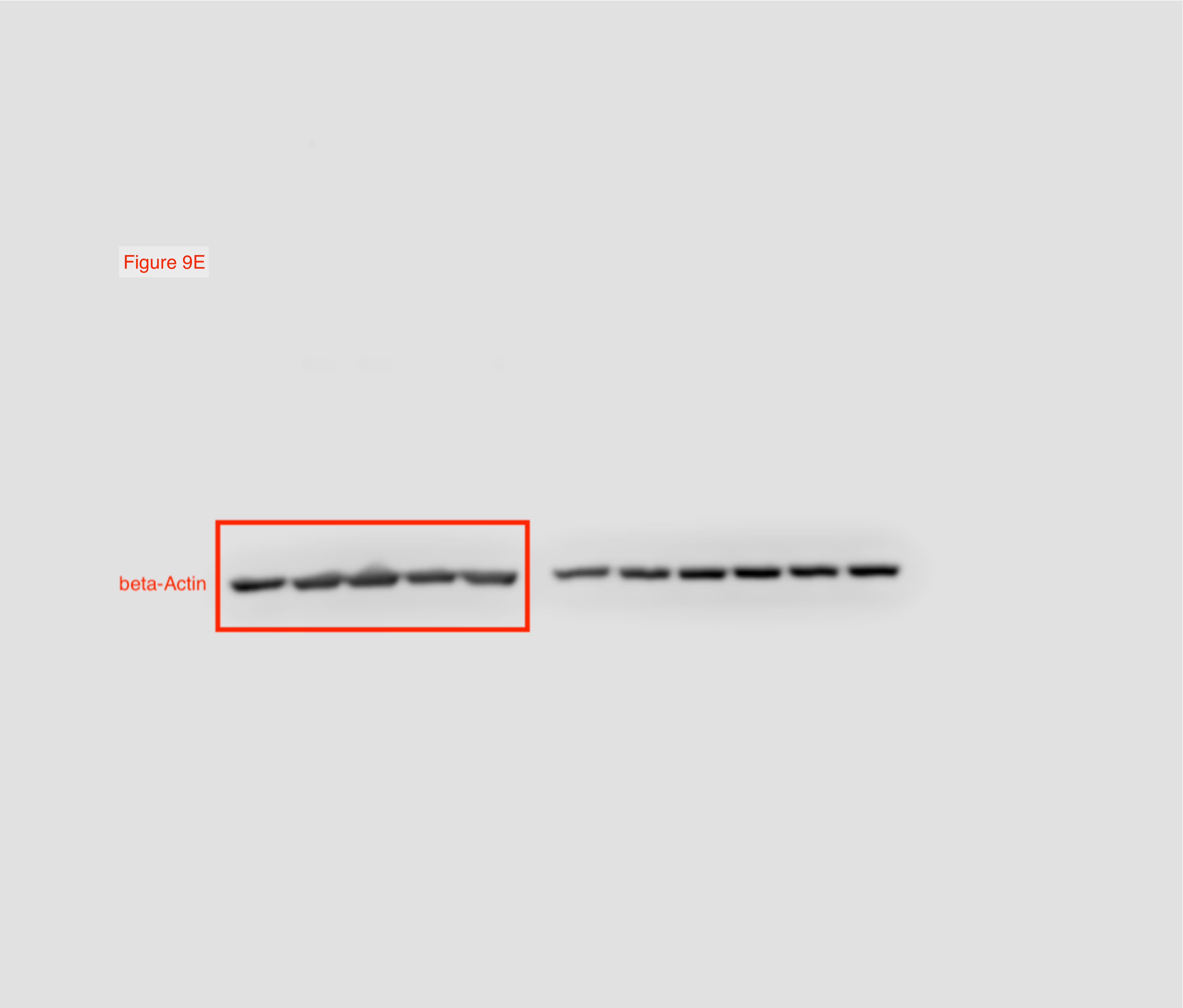

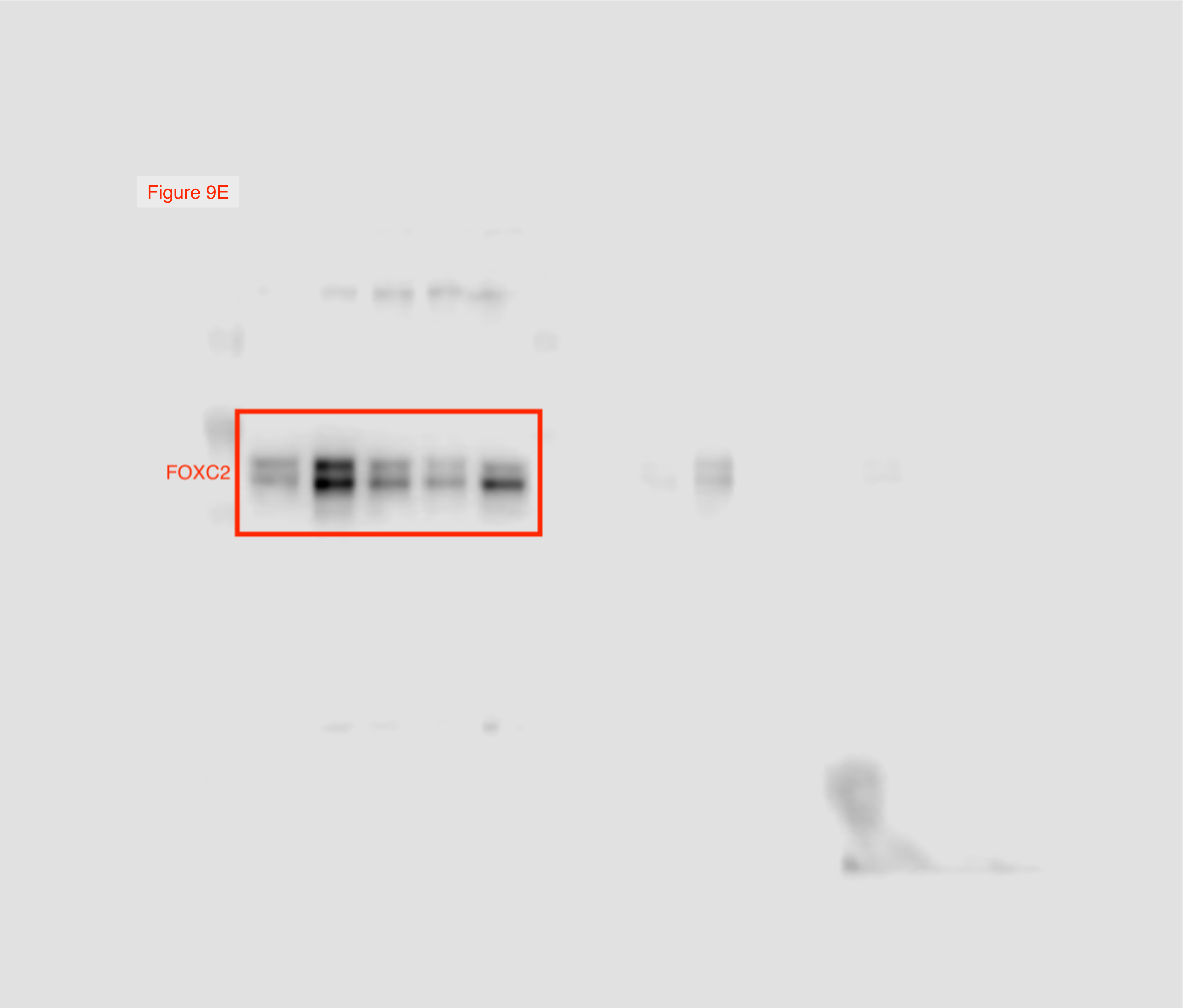
Figure 9E
FOXC2

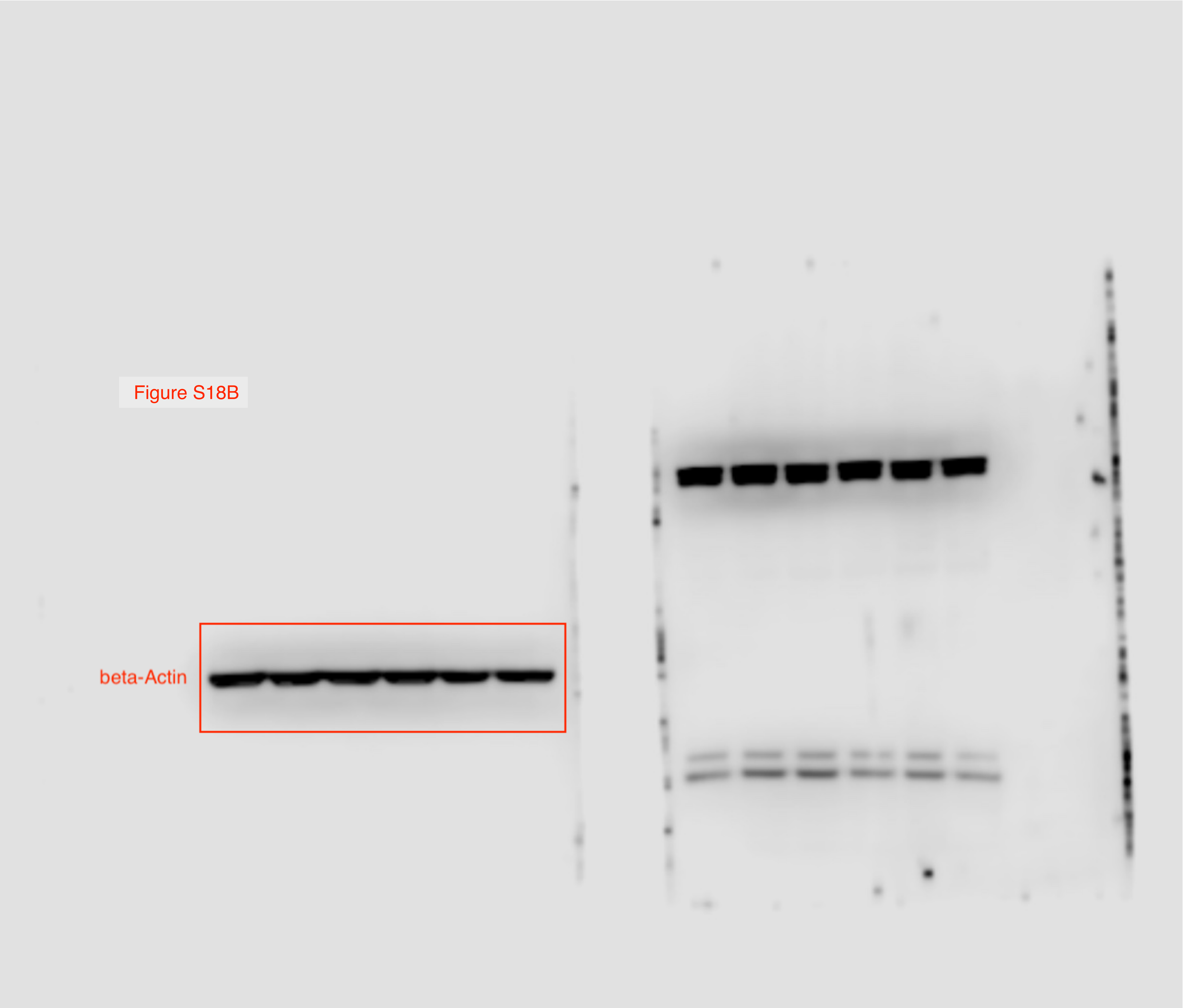
Figure S18B
beta-Actin

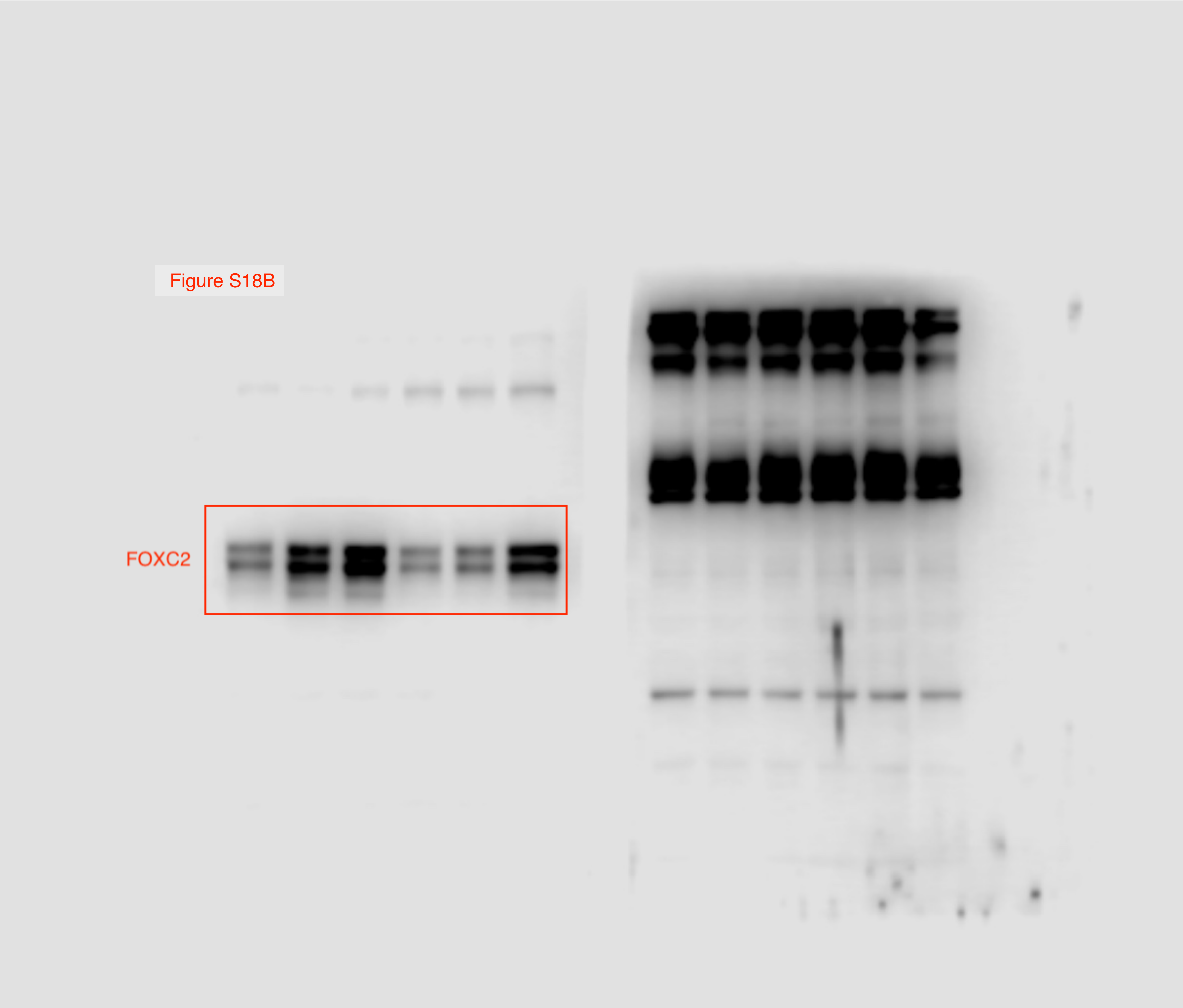
Figure S18B
FOXC2

Figure S24E

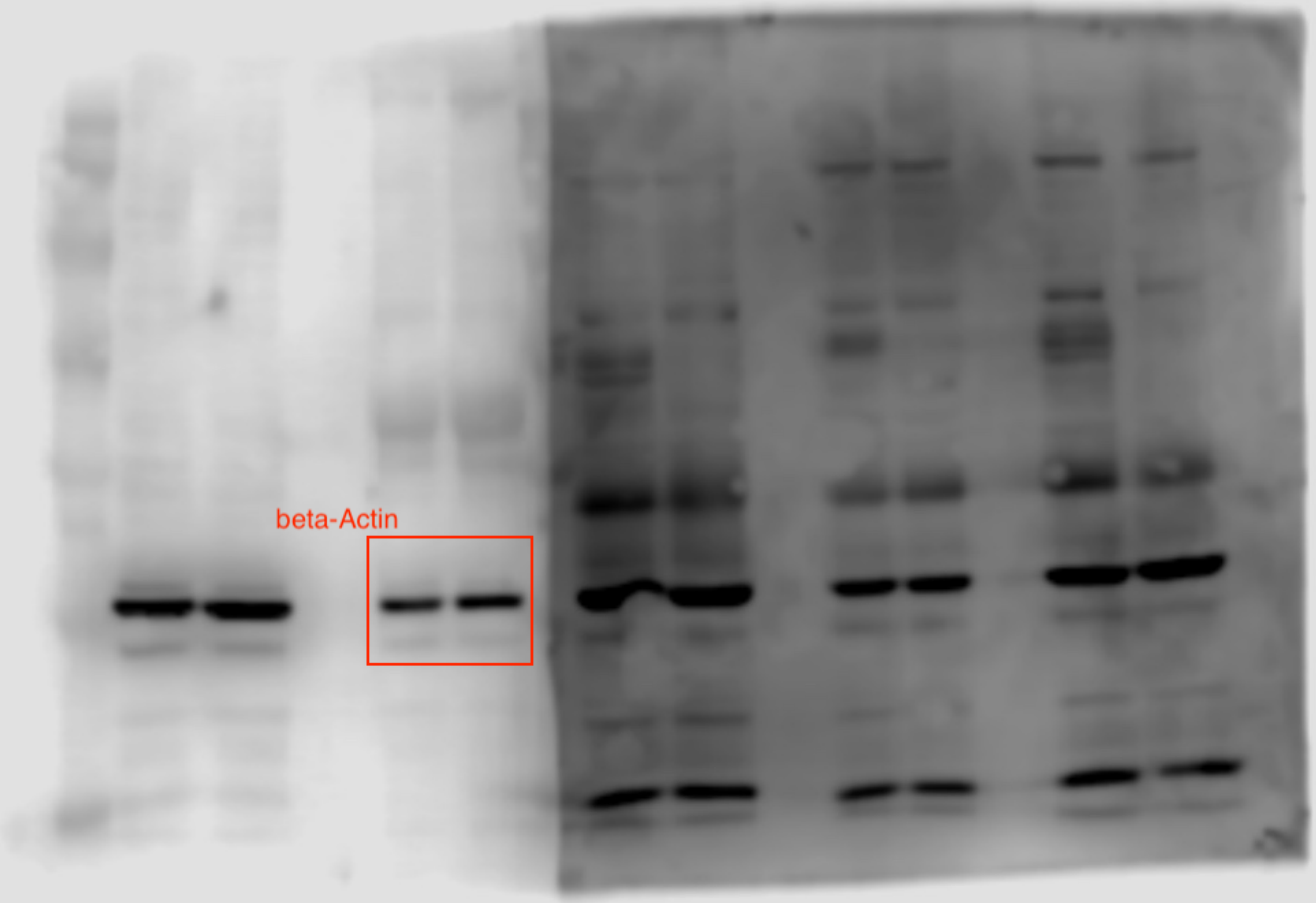

Figure S24E

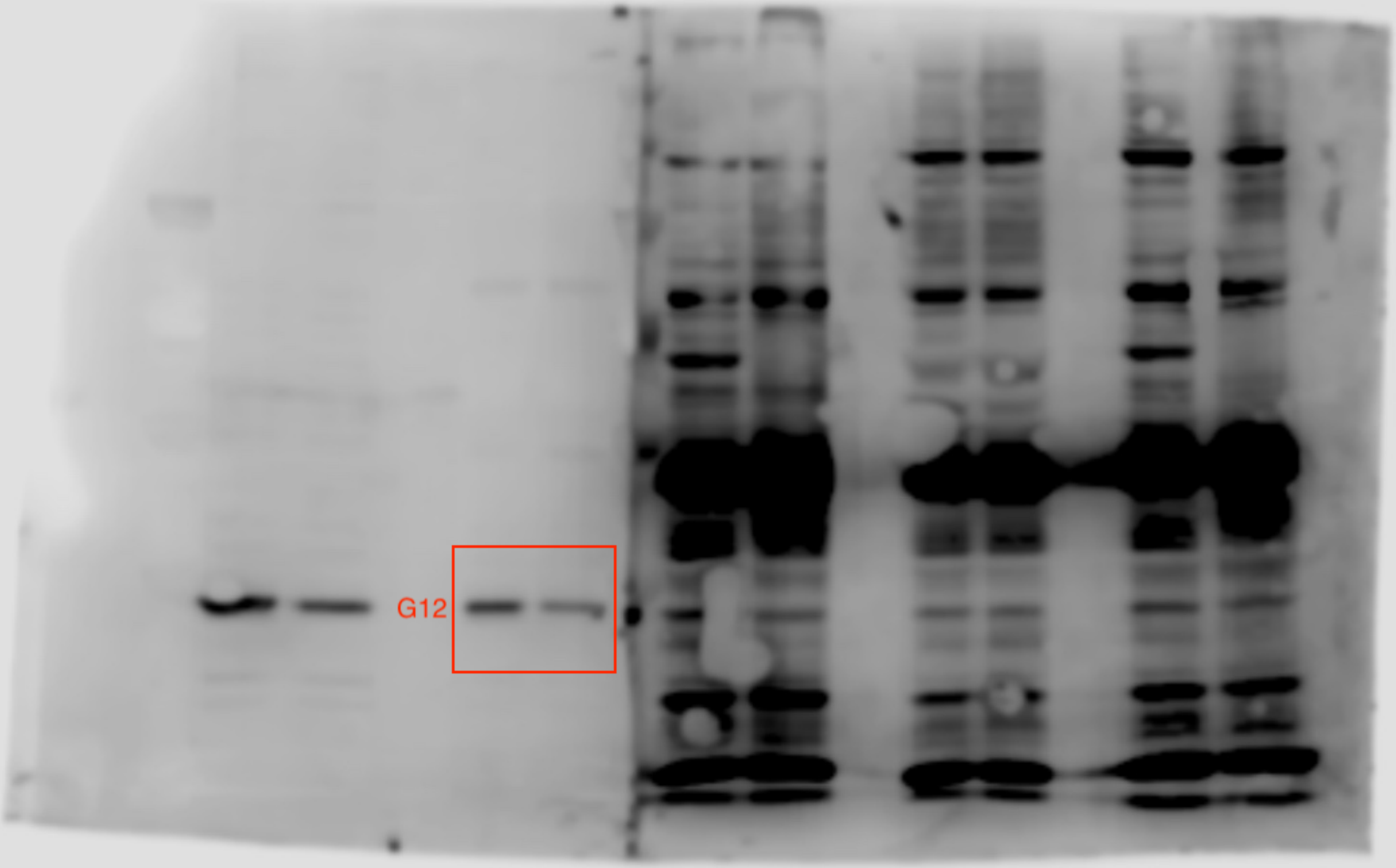

Figure S24E

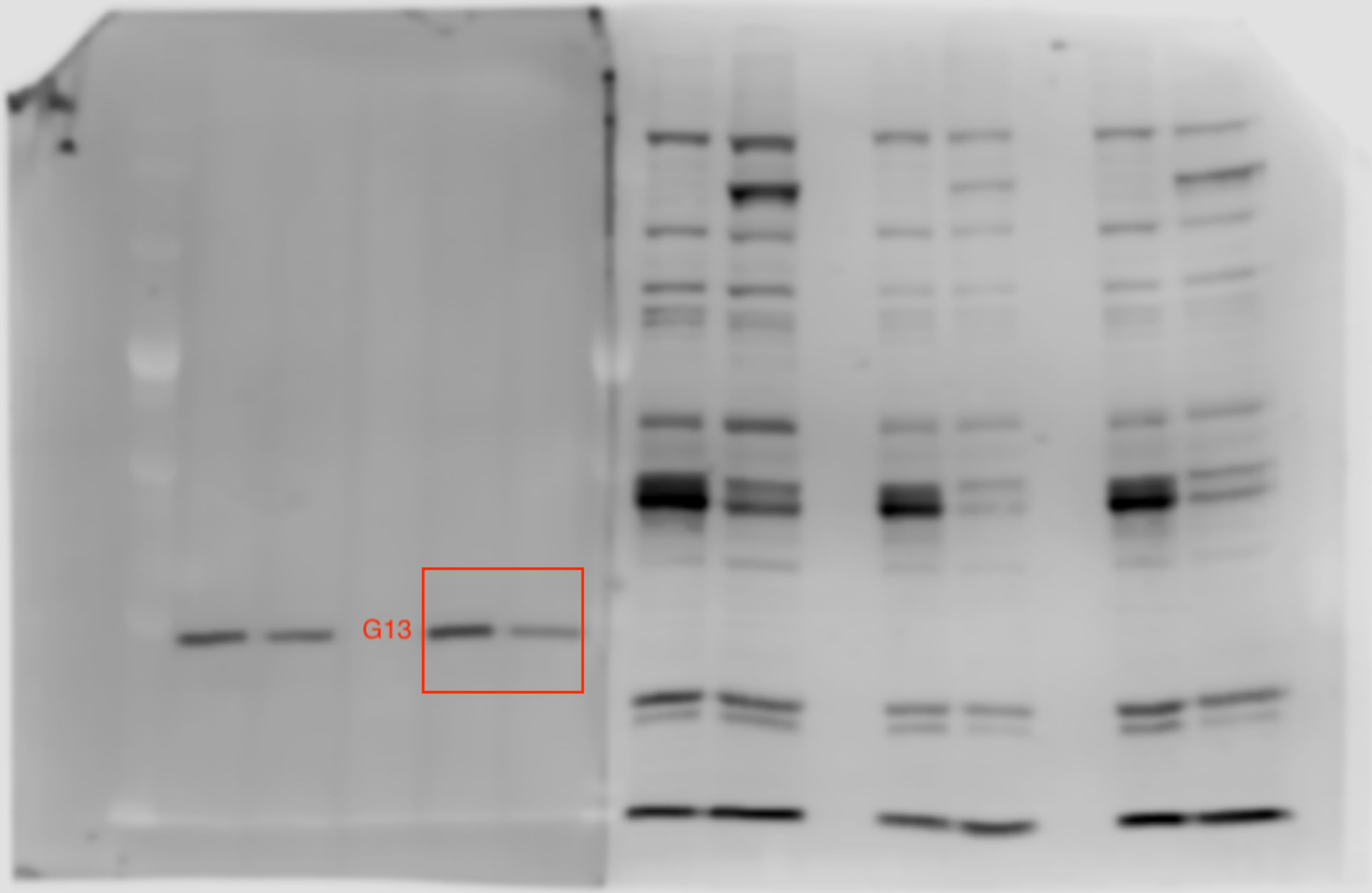

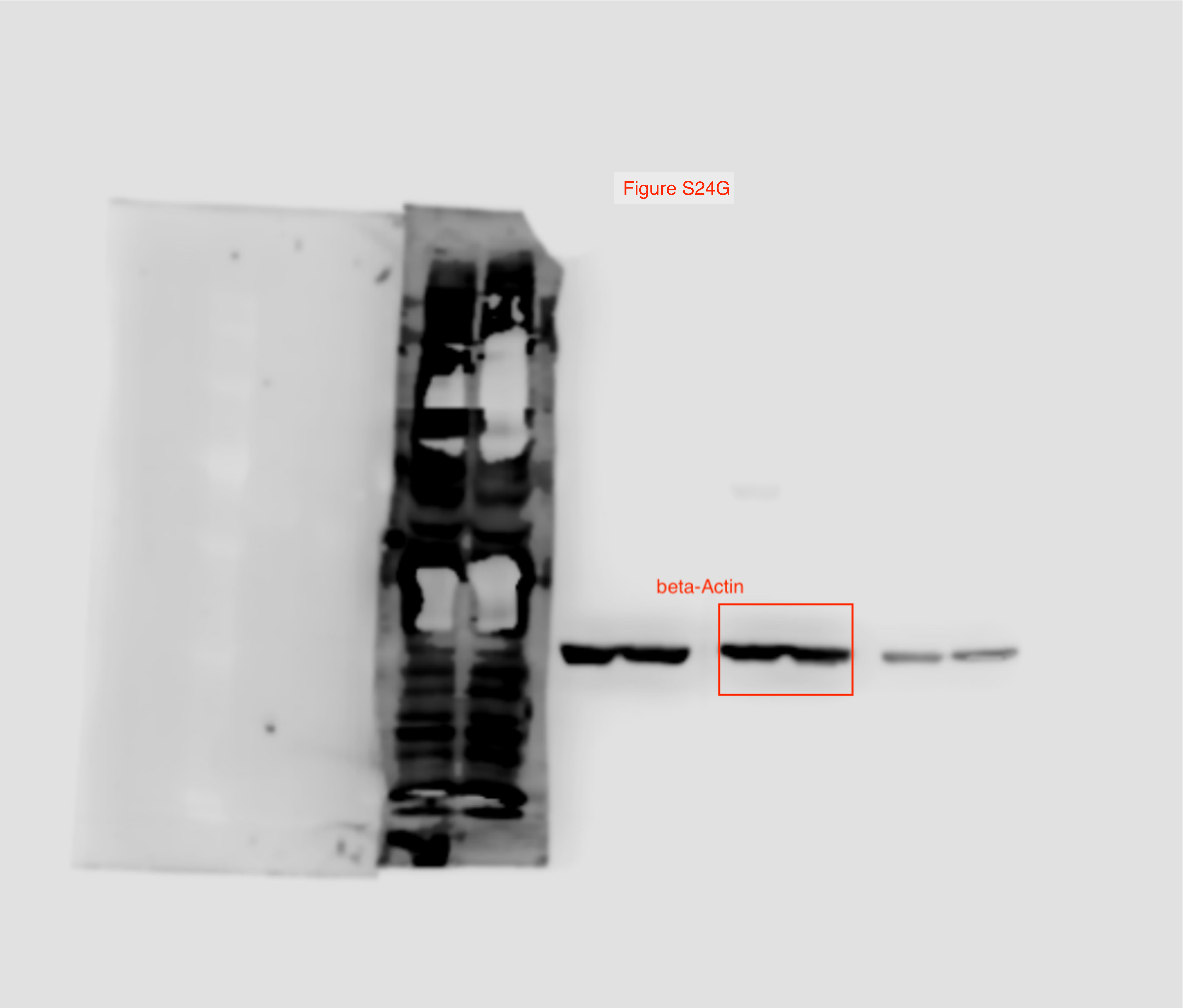
Figure S24G
beta-Actin

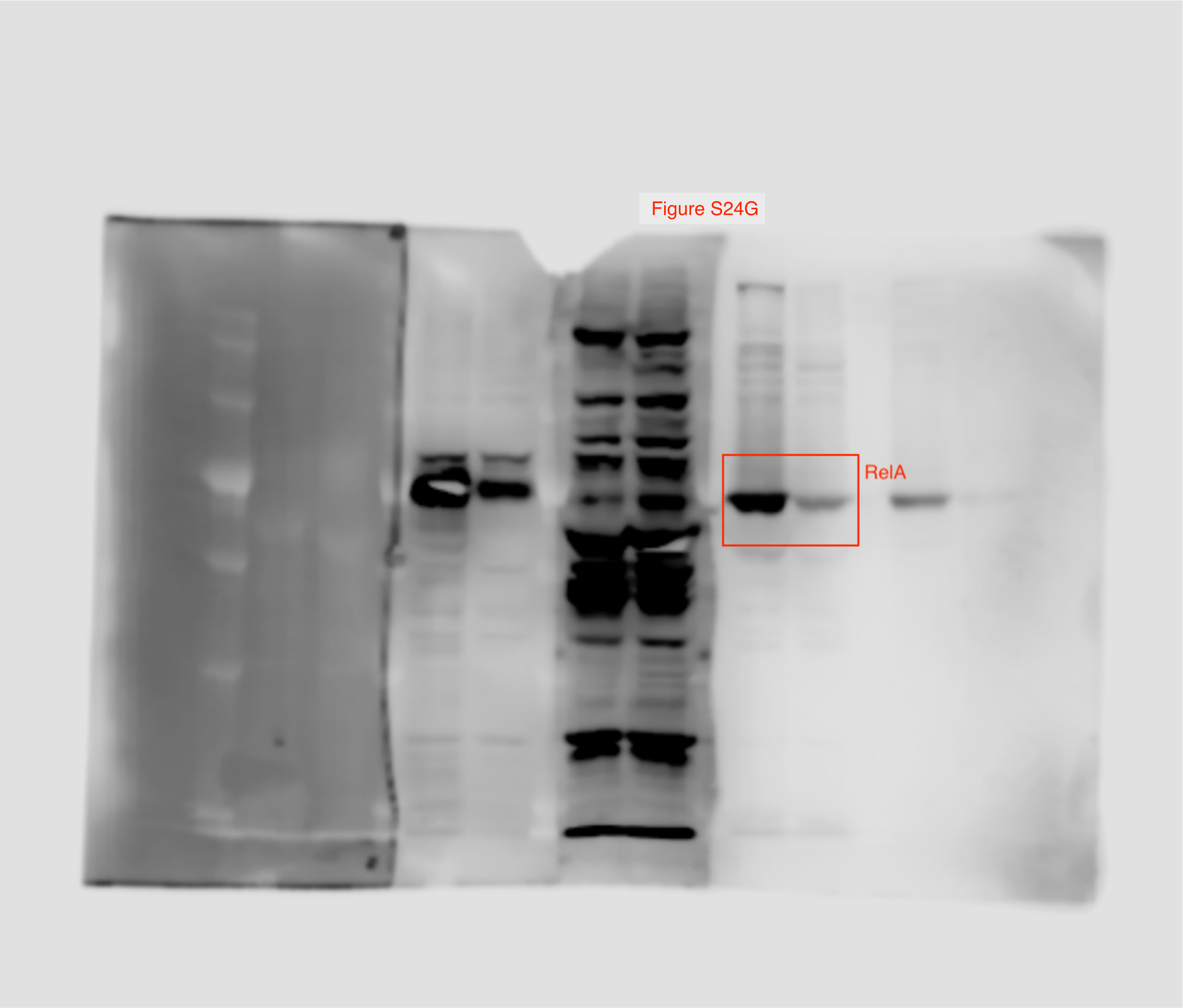
Figure S24G
RelA
